# Phylogenomics and comparative genomics of *Lactobacillus salivarius*, a mammalian gut commensal

**DOI:** 10.1099/mgen.0.000115

**Published:** 2017-06-13

**Authors:** Hugh M.B. Harris, Maxence J. B. Bourin, Marcus J. Claesson, Paul W. O'Toole

**Affiliations:** School of Microbiology, University College Cork, Cork, Munster, Ireland

**Keywords:** comparative genomics, *Lactobacillus salivarius*, bioinformatics

## Abstract

The genus *Lactobacillus* is a diverse group with a combined species count of over 200. They are the largest group within the lactic acid bacteria and one of the most important bacterial groups involved in food microbiology and human nutrition because of their fermentative and probiotic properties. *Lactobacillus salivarius*, a species commonly isolated from the gastrointestinal tract of humans and animals, has been described as having potential probiotic properties and results of previous studies have revealed considerable functional diversity existing on both the chromosomes and plasmids. Our study consists of comparative genomic analyses of the functional and phylogenomic diversity of 42 genomes of strains of *L. salivarius* using bioinformatic techniques. The main aim of the study was to describe intra-species diversity and to determine how this diversity is spread across the replicons. We found that multiple phylogenomic and non-phylogenomic methods used for reconstructing trees all converge on similar tree topologies, showing that different metrics largely agree on the evolutionary history of the species. The greatest genomic variation lies on the small plasmids, followed by the *repA*-type circular megaplasmid, with the chromosome varying least of all. Additionally, the presence of extra linear and circular megaplasmids is noted in several strains, while small plasmids are not always present. Glycosyl hydrolases, bacteriocins and proteases vary considerably on all replicons while two exopolysaccharide clusters and several clustered regularly interspaced short palindromic repeats-associated systems show a lot of variation on the chromosome. Overall, despite its reputation as a mammalian gastrointestinal tract specialist, the intra-specific variation of *L. salivarius* reveals potential strain-dependant effects on human health.

## Abbreviations

AH, alimentary health; ANI, average nucleotide identity; BLAST, Basic Local Alignment Search Tool; CCuG, Culture Collection, University of Gothenburg; CECT, Colección Española de Cultivos Tipo; CRISPR, clustered regularly interspaced short palindromic repeats; COG, clusters of orthologous groups; DSM, Deutsche Sammlung von Mikroorganismen; EPS, exopolysaccharide; GIT, gastro-intestinal tract; GH, glycosyl hydrolase; GT, glycosyl transferase; GTR, general time reversible; HGT, horizontal gene transfer; JCM, Japan Collection of Microorgansims; KEGG, Kyoto Encyclopaedia of Genes and Genomes; LAB, lactic acid bacteria; LMG, Laboratorium voor Microbiologie, Universiteit Gent; NCBI, National Center for Biotechnology Information; NCIMB, National Collection of Industrial, Food and Marine Bacteria; POCP, percentage of conserved proteins; RBB, reciprocal best BLAST; SNP, single nucleotide polymorphism; UCC, University College Cork.

## Data Summary

GenBank accession number for the whole-genome shotgun sequence of 01M14315: MSCR00000000.

GenBank accession number for the whole-genome shotgun sequence of AH4231: NBEY00000000.

GenBank accession number for the whole-genome shotgun sequence of AH4331: NBEX00000000.

GenBank accession number for the whole-genome shotgun sequence of AH43324: NBEW00000000.

GenBank accession number for the whole-genome shotgun sequence of AH43348: NBEV00000000.

GenBank accession number for the whole-genome shotgun sequence of CCUG 2753OB: NBEU00000000.

GenBank accession number for the whole-genome shotgun sequence of CCUG 38008: NBET00000000.

GenBank accession number for the whole-genome shotgun sequence of CCUG 44481: NBES00000000.

GenBank accession number for the whole-genome shotgun sequence of CCUG 45735: NBER00000000.

GenBank accession number for the whole-genome shotgun sequence of CCUG 47171: NBEQ00000000.

GenBank accession number for the whole-genome shotgun sequence of CCUG 47825: NBEP00000000.

GenBank accession number for the whole-genome shotgun sequence of CCUG 47826: NBEO00000000.

GenBank accession number for the whole-genome shotgun sequence of DSM 20554: NBEM00000000.

GenBank accession number for the whole-genome shotgun sequence of DSM 20492: NBEN00000000.

GenBank accession number for the whole-genome shotgun sequence of gul1: NBEL00000000.

GenBank accession number for the whole-genome shotgun sequence of gul2: NBEK00000000.

GenBank accession number for the whole-genome shotgun sequence of JCM 1040: NBEJ00000000.

GenBank accession number for the whole-genome shotgun sequence of JCM 1042: NBEI00000000.

GenBank accession number for the whole-genome shotgun sequence of JCM 1044: NBEH00000000.

GenBank accession number for the whole-genome shotgun sequence of JCM 1045: NBEG00000000.

GenBank accession number for the whole-genome shotgun sequence of JCM 1047: NBEF00000000.

GenBank accession number for the whole-genome shotgun sequence of JCM 1230: NBEE00000000.

GenBank accession number for the whole-genome shotgun sequence of L21: NBED00000000.

GenBank accession number for the whole-genome shotgun sequence of LMG 14476: NBEC00000000.

GenBank accession number for the whole-genome shotgun sequence of LMG 14477: NBEB00000000.

GenBank accession number for the whole-genome shotgun sequence of NCIMB 702343: NBEA00000000.

GenBank accession number for the whole-genome shotgun sequence of NCIMB 8816: NBDZ00000000.

GenBank accession number for the whole-genome shotgun sequence of NCIMB 8817: NBDY00000000.

GenBank accession number for the whole-genome shotgun sequence of NCIMB 8818: NBDX00000000

Data file 1 has been deposited in figshare; DOI: dx.doi.org/10.6084/m9.figshare.4577917.v1

Data file 2 has been deposited in figshare; DOI: dx.doi.org/10.6084/m9.figshare.4577947.v1

Data file 3 has been deposited in figshare; DOI: dx.doi.org/10.6084/m9.figshare.4577950.v1

Data file 4 has been deposited in figshare; DOI: dx.doi.org/10.6084/m9.figshare.4577953.v1

Data file 5 has been deposited in figshare; DOI: dx.doi.org/10.6084/m9.figshare.4577956.v1

Data file 6 has been deposited in figshare; DOI: dx.doi.org/10.6084/m9.figshare.4577965.v1

Data file 7 has been deposited in figshare; DOI: dx.doi.org/10.6084/m9.figshare.4577971.v1

Data file 8 has been deposited in figshare; DOI: dx.doi.org/10.6084/m9.figshare.4577977.v1.

## Impact Statement

*Lactobacillus* is an important group of bacteria that is used in food preservation, food preparation and probiotics. The group is unusually diverse, with individual species showing considerable variation in their functional properties; results of numerous studies have indicated that this variation is also present across strains within species of the genus *Lactobacillus*, leading to strain-dependent health benefits. *Lactobacillus salivarius* is one of about 200 species (including subspecies) within this group and results reported in previous literature have revealed its role as a potential probiotic. Our study uses a dataset of 42 strains of *L. salivarius* and a comparative genomic approach to define genes that each strain has in common and genes that show presence–absence distributions across the strains. Bacteria are subject to selective gene loss as well as gene acquisition. Several mechanisms, collectively known as horizontal gene transfer (HGT), contribute to the process of gene acquisition. These processes lead to considerable variation in gene content within a species, which is further promoted by the presence of independently replicating sequences (replicons) called plasmids that are often present in bacterial cells and can be transferred between cells by one of several mechanisms. A description of functional diversity across *L. salivarius* and its distribution over the replicons will increase current knowledge and possible exploitation of the variation found in members of the genus *Lactobacillus*.

## Introduction

The genus *Lactobacillus* is a diverse, paraphyletic group with a combined species and subspecies count of over 200 [1]. Lactobacilli are Gram-positive, rod-shaped, non-spore-forming bacteria that inhabit a wide range of niches from soil and plants to the gastrointestinal tracts of humans and animals [2, 3]. They are the largest group within the lactic acid bacteria (LAB) and one of the most important bacterial groups involved in food microbiology and human nutrition because of their fermentative and probiotic properties [2].

Several pivotal studies have called for a reclassification of the *Lactobacillus* genus [1, 2, 4] while others have provided detailed characterisation of its diversity [1, 2, 4–7]. Sun *et al.* recently conducted an international genome sequencing initiative of the lactobacilli that revealed that the genus was more diverse than a typical taxonomic family and that confirmed that *Leuconostoc*, *Oenococcus*, *Weissella*, *Pediococcus* and *Fructobacillus* all branch from within the *Lactobacillus* phylogenetic tree [1].

Numerous studies have also focused on the comparative genomics of individual species of the genus *Lactobacillus*, highlighting considerable intraspecific genomic diversity among strains [8–19]. One species that has been repeatedly isolated from the gastro-intestinal tracts of humans and animals and that has potential probiotic properties is the facultatively heterofermentative species, *Lactobacillus salivarius* [20–22].

The genome of *L. salivarius* UCC118 was first characterised by Claesson *et al.* and shown to have a multi-replicon organisation with a single *repA*-type megaplasmid and two smaller plasmids. The megaplasmid harboured genes with an array of functions including bile salt hydrolysis, carbohydrate metabolism and genes that complete the pentose phosphate pathway. It was concluded that the megaplasmid increased the metabolic flexibility and competitiveness of the species [20]. A previous study also identified a novel bacteriocin, Abp118, encoded by the megaplasmid of UCC118 [23]. Two exopolysaccharide (EPS) production gene clusters were found on the UCC118 chromosome, which share homology and synteny with those of other strains of *L. salivarius* [18]. EPS, among other bacterial factors, has been implicated in bile tolerance in species including *Lactobacillus rhamnosus* [24].

Results of two studies indicated that other strains of *L. salivarius* share a similar multi-replicon organisation to that of UCC118, each having a homologous *repA*-type megaplasmid and a varying number of smaller plasmids from none to two [25, 26]. Several strains have more complicated architectures: JCM1046, JCM1047 and AH43348 all have a linear megaplasmid [25] as well as a *repA*-type megaplasmid while JCM1046 also has an additional circular megaplasmid [27]. The varying presence of plasmids in *L. salivarius* as well as the variation in size of the megaplasmids [25] (100–380 kb) indicates that there is considerable functional diversity across the strains. This variation is not limited to the plasmids. Raftis *et al.* used the two chromosomal EPS clusters of UCC118 as a reference in a comparative genome hybridisation (CGH) experiment that revealed considerable divergence in gene synteny and gene presence among 33 strains of *L. salivarius* [18].

The previous study by Raftis *et al.* constituted a largely non-bioinformatic analysis of strains of *L. salivarius* but nevertheless revealed interesting functional differences [18]. The present study seeks to conduct a fully bioinformatic analysis of the phylogeny and functional divergence in an expanded dataset of genomes of 42 strains of *L. salivarius*. The constraint of using a reference strain (UCC118) that CGH demands is not a limiting factor of the present study, and strain-specific as well as clade-specific genes and functions can be identified by comparative genomics that would otherwise be excluded. We focussed on the analysis of numerous functional traits and we also provide an overall whole-genome view of the relatedness of the strains and the extent of their diversity.

## Methods

### Sequencing, assembly and annotation

The genomes of a panel of 29 strains of *L. salivarius* were sequenced by Macrogen (Beotkkot-ro-Geumcheon-qu, Seoul, Republic of Korea) using the HiSeq platform and 100 bp paired-end reads. This dataset was supplemented by 13 genomes (5 complete and 8 draft) of strains of *L. salivarius* that were available in NCBI databases. *Lactobacillus hayakitensis* DSM18933^T^ was also included in the study as a related outgroup. The dataset included both genome sequences for the type strain of *L. salivarius* from two different culture collections (DSM20555^T^ and ATCC11741^T^) to test the robustness of the methods.

Reads for the 29 sequenced genomes were assembled using Velvet (v1.2.10) [28] with a kmer count of 61, and with expected coverage and coverage cut-off both set to ‘auto’, allowing Velvet to infer these values. Nucleotide coverages were all high (>100×) and assembly statistics are available in Table S1 (available in the online Supplementary Material). Mauve (v2.4.0) [29] was used to reorder and reorient draft contigs relative to the complete genome of UCC118. Additional quality checks are described in the Supplementary Methods.

Genes were predicted using three different gene prediction software: Glimmer3 (v3.02) [30], GeneMark.HMM (v1.1) [31] and MetaGene [32]. In cases where software predictions disagreed on the correct start site for a gene, the longest predicted gene sequence was chosen. Genes predicted by one software package only were still included in the dataset in order to minimise false -egative gene predictions.

The issue of multi-copy genes such as the 16S rRNA gene is not addressed in this study. Our dataset contains a majority of draft genome sequences where assembly software often fails to assemble multiple copies of identical or almost identical genes due to ambiguous placement of reads. Similar genes that posed no problem for assembly software were included in gene counts analysis.

The amino acid sequences of predicted genes were BLASTed (blastp) against the Kyoto Encyclopaedia of Genes and Genomes database (KEGG) [33], the Clusters of Orthologous Groups (COG) database [34] and the non-redundant NCBI database (www.ncbi.nlm.nih.gov) to assign functional annotation. blast thresholds for assigning the function of a reference sequence to a query gene were 40 % identity, 50 % alignment length to the query gene and a blast bit score of 60. Prediction and annotation of specific functional groups in this study are described in the Supplementary Methods.

### Core-gene and single-gene phylogeny

QuartetS [35] was used to cluster predicted genes (amino acid sequences) into orthologs. It does this by calculating the reciprocal best blast hits (RBBs) between the genes of each pair of genomes and performing two-stage clustering (single-linkage and Markov clustering) on the RBBs. blast thresholds were 40 % identity, 50 % alignment length of the query gene and a blast bit score of 50. For clustering the RBBs, an MCL inflation value of 3 and a minimum cluster size of 2 were used.

The 42 *L. salivarius* genomes and the *L. hayakitensis* DSM18933^T^ genome combined had a predicted core genome of 938 genes. For each genome, these 938 genes were concatenated and the resulting sequences were aligned across the genome set using Muscle (v3.8.31) [36]. Gap regions were removed in R (v3.2.3) [37] where each amino acid position in the alignment is a column and all columns with at least one gap are excluded. RAxML (v8.0.22) [38] was used to generate a bootstrapped tree (100 iterations) from the core gene alignment using a PROTCATCPREV model and FigTree (v1.4.0) [39] was used to visualise the tree, which was rooted on *L. hayakitensis* DSM18933^T^. The root branch was artificially shortened to provide greater visual discrimination across *L. salivarius* sub-clades so all other branches are informative relative to each other.

To supplement the core-gene phylogeny, four single-gene phylogenies were also generated based on nucleotide sequences using the above methods and a generalised time reversible CAT model. These four genes are *groEL*, *rpsB*, *parB* and *rpoA*, which were identified in each genome using reference sequences from UCC118.

### Core-genome and pan-genome curves

A binary gene matrix modified from the QuartetS output was used to generate core-, pan- and new-gene curves in R. *L. hayakitensis* DSM18933^T^ was excluded from this analysis. Unique genes that were excluded by QuartetS (due to a minimum cluster size of 2) were also added to the matrix at this point. The numbers of core, pan and new genes were calculated by starting with two genomes and sequentially adding genomes, one at a time, until all 42 genomes were included. This procedure was repeated 1000 times, each time the order of the matrix being permuted to randomise the order of addition of genomes. Median values along with the variation from each permutation were recorded and plotted using R. In order to assess the open or closed nature of a pan-genome, the log_10_ median values for the new-gene curve were also plotted, where a slope of less than 1 is interpreted as indicating an open pan-genome (α<1) [40]. The R code for permuting the binary-gene matrix and creating a pan-genome matrix for plotting the pan-genome curve is on figshare (see Data Bibliography; data file 1). Similar code was used for the core- and new-gene curves (data file 2 and data file 3, respectively).

### Whole-genome comparisons: ANI and POCP

Two whole-genome comparative metrics were used to supplement the core-gene and single-gene phylogenies. Average Nucleotide Identity (ANI) [41] and Percentage of Conserved Proteins (POCP) [42] are two widely employed methods that seek to provide accurate species and genus cut-off values, respectively. To calculate ANI values for each pair of genomes, an ANI Perl script was downloaded (https://github.com/chjp/ANI/blob/master/ANI.pl) and implemented. Qin *et al.* [42] did not provide a POCP script so an in-house script was written using the same formula and blast thresholds listed in their paper. The script used for POCP calculation is on figshare (see Data bibliography; data file 4).

### Additional methods sections

Additional descriptions of Methods can be found in Supplementary Methods. These have the sub-headings, ‘Quality assessment of genomes’, ‘Assigning contigs to replicons’ and ‘Specific functional groups’.

## Results and Discussion

### A dataset of 42 genomes is sufficient to capture the *L. salivarius* core genome but not to capture the diversity of accessory genes

The core genome of *L. salivarius* consisted of 1236 genes. Applying a leave-one-out-strategy to the 42 *L. salivarius* genomes and re-computing the core genome indicated that it varies from 1236 to 1246 with 1281 as an outlier when JCM1230 is excluded. Table S1 shows that the JCM1230 strain sequenced in this study possesses no plasmids, which explains why the core genome increased so much when the strain was excluded – the absence of a megaplasmid excludes all extrachromosomal genes from being part of the core genome. Li *et al.* [25] identified a *repA-*type megaplasmid in JCM1230 and predicted its size to be approximately 100 kb. It is difficult to explain the absence of plasmid sequences in JCM1230 in the current study: the megaplasmid might have been artificially excluded by a procedural artefact during the DNA extraction/preparation procedure or, alternatively, since 100 kb is the smallest *repA-*type megaplasmid in the Li *et al.* [25] dataset, the strain may have lost the megaplasmid *in vitro* during laboratory passage.

**Fig. 1.**
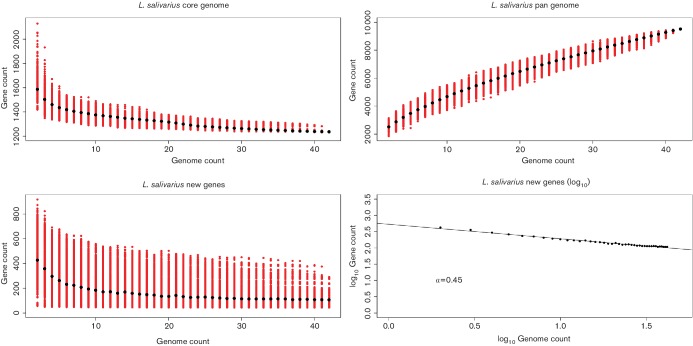
A dataset of 42 genomes is not sufficient to define the *L. salivarius* pan-genome. The four panels show, with the sequential addition of 42 *L. salivarius* genomes (*x*-axis), the decrease in core genes (a; top-left), the increase in total genes (b; top-right), the decrease in new genes (c; bottom-left) and the log of the decrease in new genes (d; bottom-right). Genes are counted as orthologous gene families (percentage identity >=40 and percentage alignment length >=50) except for genes unique to each genome. The order of addition of genomes has been permuted 1000 times. Red dots show the variation in values while black dots show the median value. An alpha value of 0.44 shows that the pan genome of *L. salivarius* is open (α<1).

Fig. 1(a) shows the core gene curve for the 42 *L. salivarius* genomes. The curve starts to plateau after the addition of only a few genomes and has substantially levelled out by genome number 42. This suggests that a dataset of 42 genomes is sufficient to define the core genome of *L. salivarius*. Hutchison *et al.* [43] recently conducted a study on the synthesis of a minimal bacterial genome that required 473 genes to survive under lab conditions. Like many other species, the core genome size of *L. salivarius*, with approximately 1200 genes, indicates that most of the core genes of a specific group of bacteria are necessary for processes outside of basic cell viability such as niche adaptation and interaction with competitors and pathogens.

The accessory genomes of the 42 strains of *L. salivarius* (excluding unique genes) consists of 3057 gene clusters ranging from 802 genes present in only two genomes to 109 genes present in 41 genomes (all but one). Fig. 1(b) shows the pan-genome curve (core and accessory, including unique genes) for the 42 genomes of strains of *L. salivarius*. The steep slope indicates that the current dataset is not large enough to define the accessory genome of *L. salivarius* and that the addition of more genomes from other strains would continue to increase the size of the accessory gene set. Fig. 1(c) shows that the new-gene curve plateaus off at a steady addition of approximately 100 genes per genome. The new-gene curve is a combination of accessory homologous genes and strain-specific genes although homologs might still exist that are not RBBs or that fall below cut-off values.

Overall, the data presented in Fig. 1 supports the model for an open pan-genome (Fig. 1d; α<1) [40] whereby an expanding dataset of *L. salivarius* genomes will continue to acquire novel genes. Variation in the presence of genes within species is brought about by two main processes, HGT and gene decay, both of which apparently began to act upon all *L. salivarius* strains after they diverged from their common ancestor, leading to the intra-specific variation observed in this dataset.

This intra-specific variation can be summarised in a very general sense using the median number of genes per replicon with the first and third quartiles representing inter-genome variation: chromosome = 1737 (1685, 1844); megaplasmid=249 (216, 283) and small plasmid=47.5 (23.5, 89.5).

### The core-gene phylogenetic tree of *L. salivarius* has similar topology to ANI whole-genome clusters and single-gene phylogenies

**Fig. 2.**
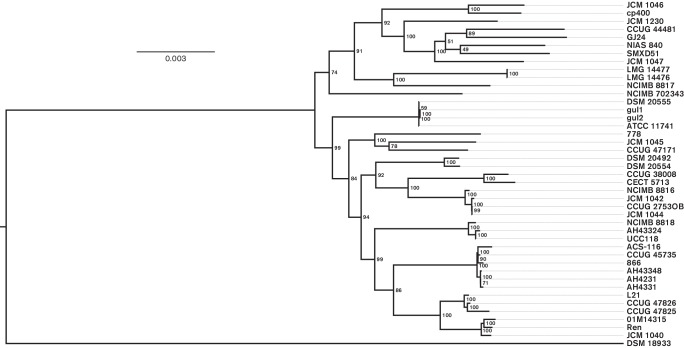
A phylogenetic tree generated from 938 core genes shows considerable variation in divergence across strains. Branch lengths (continuous black lines) represent evolutionary divergence and strain labels are lined up for ease of comparison (dashed lines). Bootstrap values are included to show robustness of tree topology. The tree is rooted on *L. hayakitensis* DSM18933^T^ and this branch is artificially reduced to provide a clearer visualisation of the other branch lengths relative to each other. Bar, average number of amino acid substitutions per site.

**Fig. 3.**
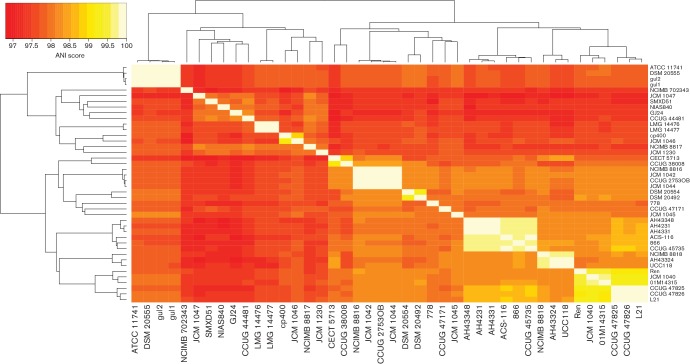
Clustering of pair-wise average nucleotide identity (ANI) scores agrees largely with the clustering of the core-gene tree in Fig. 2. The colour key (top-left) shows a gradation of colour from red to orange to yellow to white representing increasing genome-genome similarity. Euclidean distance and complete-linkage clustering were used to cluster rows and columns. *L. hayakitensis* DSM18933^T^ is excluded.

Fig. 2 shows the core-gene phylogeny of *L. salivarius*, rooted on *L. hayakitensis* DSM18933^T^. The bootstrap values are high, indicating a robust tree topology and the length of most of the branches leading to the nodes indicates that some divergence has occurred even in more closely related strains. Note that the outgroup branch (DSM18933^T^) has been shortened for this analysis (see Methods), but the scale indicating 0.003 substitutions per amino acid position can still be applied to all branches corresponding to strains of *L. salivarius*. A few sub-clades have little to no outer branch lengths, reflecting a lack of phylogenetic divergence. LMG14476 and LMG14477 have a difference of only eight SNPs in the predicted core of 938 genes even though they were isolated from different sources (Table S1). Three strains isolated from the oral cavity, gul1 and gul2 (isolated in the same study), and DSM20555^T^ (independent isolate), also show limited phylogenetic divergence (8–19 SNPs). ATCC11741^T^ is the same type strain of *L. salivarius* as DSM20555^T^ from another culture collection and they have a difference of zero SNPs in the predicted core of 938 genes, highlighting the limited accrual of variation over short periods of time during vertical gene transfer. A similar case can be observed for three strains, AH4231, AH4331 and AH43348 (17–48 SNPs), all isolated from the human ileocecal region in the same study, and between UCC118 and AH43324 (54 SNPs), also isolated from the human ileocecal region. In contrast to these sub-clades, CCUG 44481 (an animal isolate) and CCUG 38008 (a human gall isolate) have the most divergent core genome across all 42 strains of *L. salivarius* (3643 SNPs). Average Nucleotide Identity (ANI) [41] was also used to cluster *L. salivarius* strains. Fig. 3 shows a heatmap of ANI values where the clustering of strains is largely in agreement with the core-gene phylogeny of Fig. 2. *L. hayakitensis* was excluded from the heatmap so an unrooted clustering is presented. ANI was designed as a method to identify whether a particular strain belongs within a species, using a cut-off value of 95 % as the species boundary [44]. In terms of its use of homologous sequences, ANI can be compared with the core-gene phylogenetic method, although it uses nucleotide sequences and includes homologous intergenic regions. Discrepancies between the two tree topologies are likely to be due to differences in computing similarity scores from intragenic amino acid sequences and intragenic/intergenic nucleotide sequences. The lowest ANI value across the strains of *L. salivarius* is 96.8 % between JCM1047 (isolated from swine intestine) and CECT5713 (isolated from human breast milk), indicating that all strains represent members of the same species.

Single-gene phylogenies were also constructed using four marker genes, *groEL*, *rpsB*, *parB* and *rpoA*. When sub-clades had sufficient phylogenetic signal, bootstrap values were high and agreed with the tree topology of the core-gene phylogeny in Fig. 2. On average, however, the phylogenetic signal of the trees was too low to allow reliable comparisons, reflecting the limits of building single-gene trees to study the evolutionary history within a species, especially since gene sequences had to be aligned at the nucleotide level to see what little divergence there was across strains for these genes. The tree for *parB* is included as Fig. S1 since it shows the most phylogenetically informative signal of the four genes.

### Plasmids contribute considerably to *L. salivarius* genomic diversity

Li *et al.* have already shown that there is considerable size variation in *L. salivarius repA*-type megaplasmids ranging from 100 kb (JCM 1230) to 380 kb (DSM 20555^T^) [25]. This indicates that there is similar variation in functional diversity due to the high coding density of prokaryotic replicons. The number of predicted genes on the *repA*-type megaplasmids that we predicted ranged from 165 genes in NIAS840 to 408 genes in cp400. NIAS840 has a complete genome sequence while that of strain cp400 is a draft, indicating that closed genomes are not a factor for bias when predicting the number of genes on megaplasmids. The lack of plasmids in *L. hayakitensis* DSM 18933^T^ was not discussed when the description of the strain was published [45] and plasmid absence has no effect on the conclusion that the *repA*-type megaplasmid was acquired early in the evolution of *L. salivarius* [25]. The possible technical reasons for the loss of a megaplasmid in JCM 1230 have been covered in a previous section. Table S2 shows the blast results of three *repA*-type marker genes (*repA*, *repE* and *parA*) against the contigs of each genome. If contigs were assigned to replicons accurately, it is expected that blast hits for each gene would lie on predicted *repA*-type megaplasmid contigs. This is indeed the case, with all three genes having between 93% and 100 % identities over their full length aligned to a *repA*-type megaplasmid contig, usually all three genes align to the same contig. Exceptions include JCM1230, which had no blast hits due to its missing megaplasmid, AH43348, which had an extra *parA* gene on a predicted *repA*-type megaplasmid contig and *L. hayakitensis* DSM 18933^T^, which has a *repA* gene and a *parA* gene on a predicted chromosomal contig. The *repA* and *parA* genes of DSM 18933^T^ have a lower identity than the other hits (79 and 87 %, respectively) and it is possible that these genes belong to an unidentified megaplasmid, although there was no mention of extrachromosomal sequences in the original species/strain description [45].

Several strains in the dataset also possess linear megaplasmids that have little homology to the *repA*-type megaplasmid, a finding that was first documented by Li *et al.* [25]. These strains are JCM 1046, JCM 1047 and AH43348. The linear megaplasmids of JCM 1046 and JCM 1047 show high sequence similarity: two predicted contigs in the draft genome of JCM 1047 cover most of the complete linear megaplasmid of JCM 1046 (pLMP1046) with a high percentage identity. The genome of AH43348 is a draft made up of 114 contigs so the linear megaplasmid could only be predicted by sequence homology with other linear megaplasmids from the NCBI database of *Lactobacillus* plasmids (see Supplementary methods). The contigs of AH43348 had very little homology to pLMP1046; however, several contigs do cover most of a second megaplasmid present in NIAS840 aside from the contigs that align to *repA*-type megaplasmids. The second megaplasmid of NIAS840 was not described as being circular or linear [46] and it is possible that this megaplasmid is actually homologous to the linear megaplasmid of AH43348. An alternative explanation is that both AH43348 and NIAS840 have two circular megaplasmids; this would mean that the homology-based method used in this study failed to predict the linear megaplasmid of AH43348, instead assigning its genes to the chromosome. SMXD51 is predicted to have an additional large plasmid as well as a *repA*-type megaplasmid; its draft genome is made up of ten contigs, six belonging to the chromosome and the remaining four described as representing a 143 kb megaplasmid, an 85 kb large plasmid and two small plasmids (31 and 9 kb) [47]. We found that the 143 kb and the 85 kb plasmids both align over most of their sequence to different regions of the *repA*-type megaplasmid of UCC118 (pMP118), together adding up to over 94 % of its length. This indicates that these two sequences do not represent separate plasmids, but together make up the *repA*-type megaplasmid of SMXD51, a finding made more probable by the fact that the available SMXD51 genome is a draft genome.

The smaller plasmids show even greater variation. Table S1 shows that 15 strains have no small plasmids, 20 strains have a single small plasmid and 8 strains have two small plasmids. The number of predicted genes on the small plasmids ranged from 11 in a GJ24 plasmid to 144 in an AH4231 plasmid. Many of these plasmids show high-level homology to the two endogenous plasmids described by Fang *et al.* in UCC118 [26]. The small plasmid of JCM 1046 (pCTN1046) is quite distinct from those in UCC118 and shares homology with a plasmid in SMXD51, a relationship first described in Raftis *et al.* [27].

**Fig. 4.**
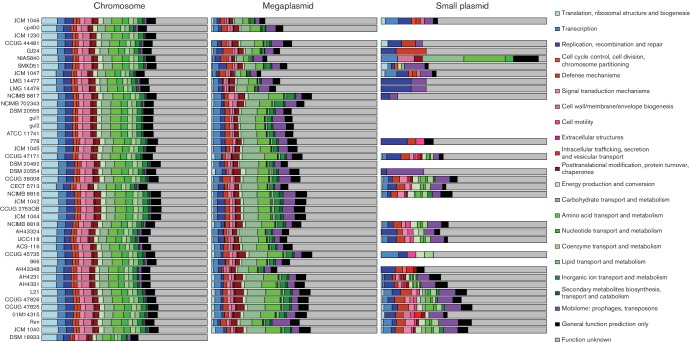
The proportion of genes assigned to each major COG category shows considerable variation across strains and plasmids. Colours and order of COG categories in each bar from left to right match the colour legend from top to bottom. The order of the strains (bars) reflects the order of the core-gene tree in Fig. 2. Genes are separated into chromosomal, megaplasmid and plasmid genes. In cases where genomes have multiple plasmids or megaplasmids, the COG counts were combined. Note that genes assigned to the linear megaplasmids of AH43348, JCM 1046 and JCM 1047 are also included in the bar plots for megaplasmids. The absence of plasmids from a particular genome is represented by the absence of a bar for that category.

Fig. 4 shows a general summary of functional diversity across the replicons for each strain using COG categories. The absence of megaplasmids in DSM 18933 and JCM 1230 is evident along with the absence of smaller plasmids in 15 strains. The proportional allocation of genes to COGs shows much more similarity across chromosomal genes than across those on megaplasmids or plasmids, reflecting the accessory nature of extrachromosomal DNA. The proportions (and raw counts) of genes involved in translation and ribosomal structure is much higher on the chromosomes, reflecting the complexity of chromosomal cellular machinery related to protein production when compared with that of the plasmids. All three replicon groups have a large number of genes with unknown function, highlighting current limits to annotation and also the need for greater experimental investigation. The mobilome gene category is much higher as a percentage in the plasmids; this makes sense due to the different selection pressures acting on plasmids, and it can be speculated that it benefits prophages and transposases to use the higher copy number and conjugative ability of plasmids to multiply.

### LPXTG-motif surface proteins are more numerous in strains harbouring multiple sortases and a putative pilus operon

**Fig. 5.**
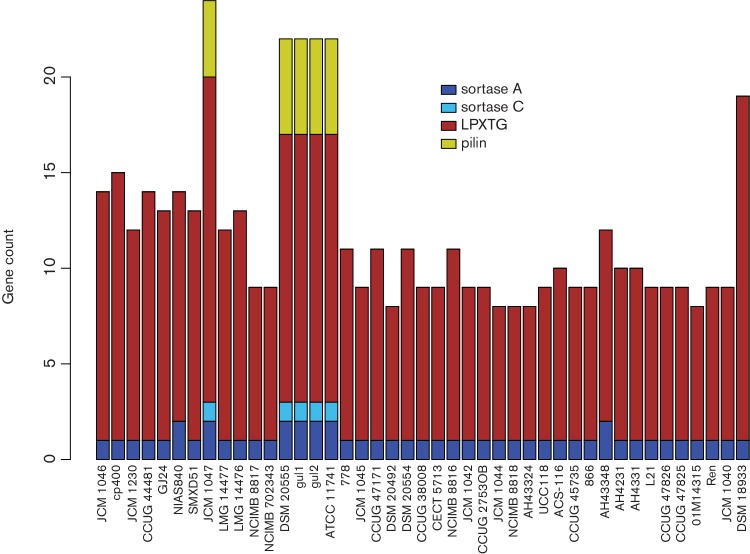
Gene counts for sortase families, LPXTG motifs and potential pilus clusters are all positively correlated. Genes are assigned to four categories, sortase A, sortase C, LPXTG and pilin, and coloured according to the legend. The order of strains (bars) from left to right reflects the order of the core-gene tree from top to bottom in Fig. 2.

Sortases are important enzymes for recognising and anchoring surface proteins containing an LPXTG motif, and sortase-anchored surface proteins are often involved in the interaction of a bacterium with its surrounding environment [48]. In *L. salivarius*, this includes host–bacterium interactions since most strains have been isolated from human or animal sources. Fig. 5 shows the gene counts for sortases, pilus genes and genes with an LPXTG motif.

All 43 genomes have at least one sortase A gene, the ‘housekeeping’ sortase, that typically acts on many protein targets and is considered to be essential for the survival of most Gram-positive bacteria. Additionally, seven genomes have an extra sortase A and five of these have a sortase C gene. All five strains with a sortase C have a putative pilus operon, confirming the results of previous studies that describe the role of sortase C in pilus construction [49]. The extra sortase A in strains with a pilus operon indicates that this gene is a more specific sortase A with some role in the formation of pili. However, two strains, NIAS840 and AH43348, also have an additional sortase A gene, but they lack a pilus operon. We described in a previous section that the non-*repA*-type megaplasmid (presumably linear on the basis of results described by Li *et al.* [25]) of AH43348 has a strong homology to the second megaplasmid in NIAS840. The extra sortase A gene in these two strains lies on this extra megaplasmid (speculatively linear) and it presumably acts on gene products with an LPXTG motif encoded by this replicon. Four of the five strains with pilus operons belong to the DSM 20555^T^ sub-clade (four genomes) where three were isolated from the oral cavity and ATCC 11741^T^ is a reference strain from the Human Microbiome Project (www.hmpdacc.org). Pili are commonly involved in adhesion and their production in this sub-clade might reflect an adaptation to the oral environment by allowing the bacterial cell to adhere to the tooth surface or underlying dentine. JCM 1047 is a swine intestinal isolate and it is not clear why it is the only other strain with a predicted pilus operon, except that the presence of pili surely has an adaptive role in the intestine as well as the oral cavity.

The range of values for gene products with an LPXTG motif is partly explained by the number of sortase genes and the presence of pilus operons, with more genes being present in strains with multiple sortases and a pilus operon. *L. hayakitensis* DSM 18933^T^ has the most genes containing an LPXTG motif (*n*=18). This indicates that there might have been selective pressure leading to a reduced number of cell-surface and secreted proteins with an LPXTG motif in *L. salivarius*.

### The gene distributions of glycosyl hydrolases and glycosyl transferases show considerable evidence of gene loss and HGT

**Fig. 6.**
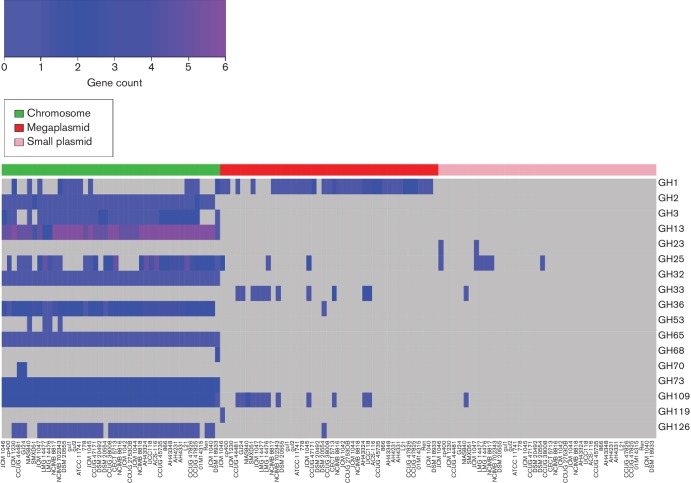
Gene counts for GH families suggest regular HGT and gene loss events. The colour key (top-left) shows a gradation of colour from blue to dark blue to purple as the gene count increases. Grey represents a gene count of zero. GH genes are separated into chromosomal, megaplasmid and plasmid genes. For each replicon group, the order of strains (columns) from left to right reflects the order of the core-gene tree from top to bottom in Fig. 2.

**Fig. 7.**
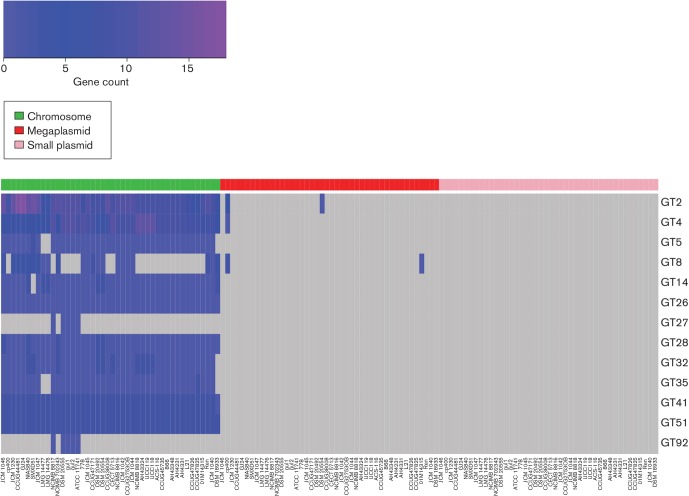
Gene counts for GT families indicate more restricted HGT than that which occurs for GHs. The colour key (top-left) shows a gradation of colour from blue to dark blue to purple as the gene count increases. Grey represents a gene count of zero. GT genes are separated into chromosomal, megaplasmid and plasmid genes. For each replicon group, the order of strains (columns) from left to right reflects the order of the core-gene tree from top to bottom in Fig. 2.

Glycosyl hydrolases (GHs) and glycosyl transferases (GTs) are two large and important groups of genes that are responsible for the hydrolysis (or modification) and synthesis, respectively, of the glycosidic bonds of carbohydrates. Figs 6 and 7 show the distribution and abundance of genes according to their GH and GT families across the 42 strains of *L. salivarius* and *L. hayakitensis* DSM 18933^T^, separated into their respective replicons.

There is no correlation between the number of GHs and the number of GTs per strain in this dataset (Spearman's ρ=−0.07; *P*=0.67), showing the independence of a strain’s ability to synthesise carbohydrates compared with its ability to break them down. This is not surprising since the selective pressures acting on genes that break down particular carbohydrates are largely determined by the availability of that substrate in the environment while carbohydrate synthesis can lead to complex interactive traits such as EPS, which vary in structure, composition and function depending on the biotic and abiotic environmental factors and the species of bacteria in question [50].

For both GHs and GTs, the majority of genes reside on the chromosome (GH 808 of 900; GT 1313 of 1322), but there is considerably more extrachromosomal diversity for GHs than GTs and no GTs are located on the smaller plasmids. These results indicate that GHs are horizontally acquired more frequently than GTs in *L. salivarius*. GT families also appear to be more stable on the chromosome compared with GHs, with 10 out of 13 GT families being present in 39 strains or more while for GHs only 7 out of 17 families are present. Greater retention of GT genes across the dataset indicates that the relevant functions of carbohydrate synthesis are under greater selective pressure across all strains, whereas GH gene retention is more variable due to the dynamic and changeable nature of carbohydrate availability in typical environments for cells of *L. salivarius*.

Numerous gene families for both GHs and GTs are present in all 43 genomes and found on the chromosomes only. For GHs, these are GH13, GH32 and GH73; for GTs, these are GT26, GT28, GT41 and GT51. All these families have numerous predicted substrates and functional properties and their absence from extrachromosomal replicons indicates that these genes are important for cell processes independent of particular niches. More interesting are the families that are present in the genomes of all 42 strains of *L. salivarius* but absent from *L. hayakitensis* DSM 18933^T^ or, alternatively, absent from all 42 strains of *L. salivarius* but present in DSM 18933^T^. These families are GH2 and GT32 (present in *L. salivarius* only), and GH68 (present in *L. hayakitensis* only). GH68 is a levansucrase and present in DSM 18933^T^ only while GH2 and GT32 are quite general and act on multiple substrates. Levansucrase enzymes, unlike sucrases, are localised almost entirely extracellularly and they contribute to 60 % of extracellular sucrase activity [51]. The presence of levansucrase in DSM 18933^T^ indicates that this strain is more adapted to the breakdown of sucrose, an ability that may compensate for the fact that this strain has the lowest number of GH genes (*n*=12) in this dataset and the lowest number of GH families (*n*=9) along with 01M14315, DSM 20492 and SMXD51.

A few other GH and GT families have very limited distributions. GH70, a dextransucrase, is limited to CCUG 44481 and GJ24, a branch pair isolated from different sources. A gene for GH119, an α-amylase, is found only on the *repA*-type megaplasmid of JCM 1046. Peptidoglycan lyase, an enzyme that can hydrolyse the cell walls of bacteria, is found on the smaller plasmids of JCM 1046 and JCM 1047, both isolates from the swine intestine. GT27 and GT92 are limited to the chromosomes of five strains: the sub-clade of four strains containing DSM 20555^T^ and the singleton, NCIMB 8817.

The distribution of genes across the strains in these two major functional groups indicates considerable gene loss and HGT with very limited association of GH and GT families with isolation source.

### Host adaptation and gene conservation in EPS gene clusters

**Fig. 8.**
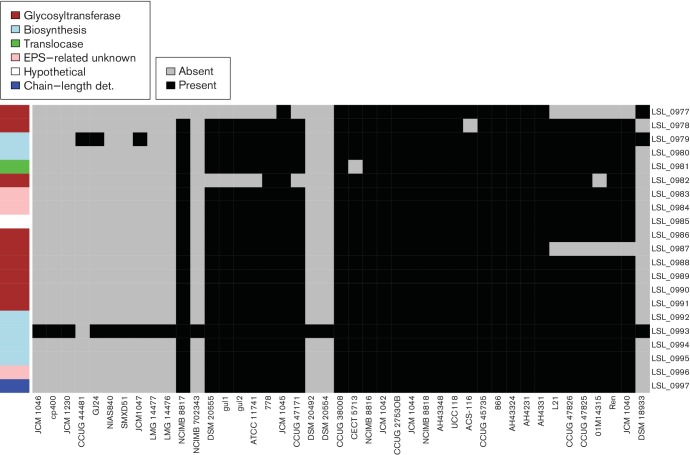
EPS cluster 1 is absent from some strains of *L. salivarius*. Grey represents gene absence and black represents gene presence. The genes (rows) are ordered according to synteny in UCC118, which is used as a reference strain. The order of strains (columns) from left to right reflects the order of the core-gene tree from top to bottom in Fig. 2. The colour legend defines genes within the cluster in terms of general function. det., Determinant.

*L. salivarius* UCC118 EPS cluster 1 is located on the chromosome and is composed of 21 genes spread across 23 kb. Of the strains of *L. salivarius* studied 29 harbour at least 18 genes from UCC118 EPS cluster 1 and the other 13 strains do not have the cluster in their genomes (Fig. 8). Interestingly, the presence of EPS cluster 1 is correlated with the core-gene tree (Fig. 2). The majority of strains in the top sub-clade from JCM 1046 to NCIMB 702343 lack EPS cluster 1. Two other strains, DSM 20492 and DSM 20554, are located in the middle of the tree and do not harbour the cluster either. DSM 18933^T^ lacks EPS cluster 1, indicating that either the common ancestor of *L. salivarius* acquired the cluster through HGT after the split from *L. hayakitenis* or, alternatively, that DSM 18933^T^ lost the cluster through gene decay.

Another interesting point is that 9 of the 13 strains lacking EPS cluster 1 were isolated from animal samples and only 3 were isolated from human samples (one strain does not have a known origin). In contrast to this, the majority of strains harbouring EPS cluster 1 have a human origin, indicating that EPS cluster 1 is not essential for the survival of *L. salivarius* as a species, but it might code for an adaptive trait to the human gastrointestinal tract.

**Fig. 9.**
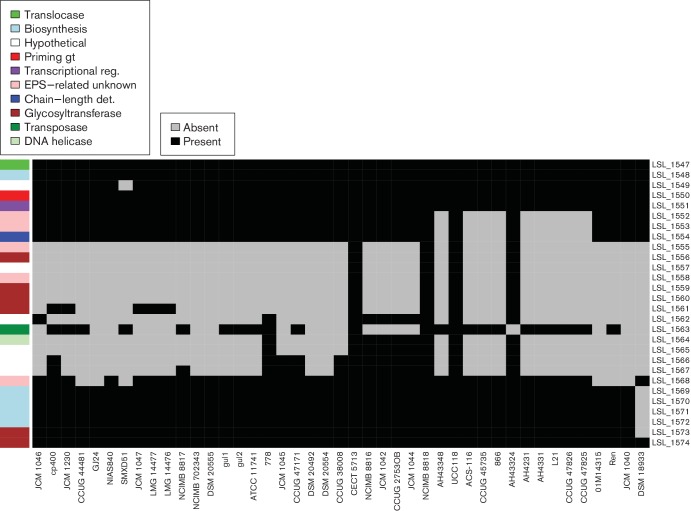
EPS cluster 2 shows variable presence of genes at the centre in *L. salivarius*. Grey represents gene absence and black represents gene presence. The genes (rows) are ordered according to synteny in UCC118, which is used as a reference strain. The order of strains (columns) from left to right reflects the order of the core-gene tree from top to bottom in Fig. 2. The colour legend defines genes within the cluster in terms of general function. gt, Glycosyl transferase; reg, regulator.

*L. salivarius* UCC118 EPS cluster 2 is also located on the chromosome and is composed of 28 genes spread across 33 kb. The two physical extremities of EPS cluster 2 are shared by all the strains (Fig. 9; from LSL_1574 to LSL_1569 and from LSL_1551 to LSL_1547). However, variations exist in the middle of EPS cluster 2 and six groups were identified as described in Fig. S2. Group 1 contained strains harbouring all the UCC118 EPS cluster 2 genes while group 6 had only the two extremities of the cluster.

The central part of the cluster varies in the *L. salivarius* strains compared with the reference strain, UCC118. This region contained the majority of glycosyltransferases and EPS biosynthesis-related proteins in UCC118 EPS cluster 2. Glycosyltransferases are involved in the addition of sugar subunits to the growing EPS chain. A difference in the glycosyltransferase composition indicates potential variation in EPS structure. These results indicate that the organisation of EPS cluster 2 is not conserved in most strains of *L. salivarius*. Indeed, only four strains belong to group 1: UCC118, AH43324, CECT 5713 and NCIMB 8818. Interestingly, potential probiotic activities have been described for CECT 5713 [52] and UCC118 [23].

EPS produced by strains of lactobacilli are suspected to play a role in the strain’s probiotic activity [53]. *L. salivarius* heteropolysaccharide production is controlled by EPS clusters and the structure of *Lactobacillus* EPS clusters has been described as highly conserved [54], although discussion in this area is still very much open, a fact that is highlighted by *L. salivarius* EPS clusters that vary considerably in both their gene synteny and in the presence of particular genes.

### Bacteriocin gene content ranges from ubiquitous to strain-specific

Flynn *et al.* identified a small, heat-stable bacteriocin, Abp118, in UCC118 that showed considerable antimicrobial activity [23]. This bacteriocin is identified as salivaricin P by Bagel3, which has close homology to Abp118 since they differ by only two amino acids [55]. Homologs of Abp118 along with their surrounding genes (Areas of Interest; AOIs) are present in 22 strains of *L. salivarius* in this study (Table S3). In all 22 cases, this bacteriocin is found on the *repA*-type circular megaplasmid and appears to have no strong association with a particular isolation source, but its distribution on the core-gene tree (Fig. 2) is associated with several sub-clades including the UCC118 branch (*n*=3), the AH43348 branch (*n*=6) and a few small sub-branches (of *n*=2) and singletons. It is interesting that some of the strains lack this bacteriocin; the size and functional variation of the *repA*-type megaplasmid highlights the fast evolutionary rate that these replicons display, perhaps losing Abp118 if bacteria co-inhabiting the same environment did not compete strongly with *L. salivarius* for limiting resources.

A number of other bacteriocins are also present in the strains of *L. salivarius* in this dataset. All 43 strains possess between one and four enterolysin genes. The N-termini of these bacteriocins have considerable sequence homology to a bacteriophage lysin and they act to degrade the bacterial cell wall in a range of genera including enterococci, pediococci, lactococci and lactobacilli [56]. LS2, an extremely heat- and pH-stable peptide with anti-listerial activity [57], is confined to the NCIMB8816 sub-clade (*n*=4) and shows homology to bacteriocins in several oral streptococci. The two-strain sub-clade consisting of CCUG 44481 and GJ24 is the only branch to harbour a plantaricin S while MR10B is present on the small plasmid of three strains, JCM 1046, JCM 1047 and DSM 20554. A cluster of three bacteriocins is present on two divergent strains, CCUG 44481 and CCUG 47171, harbouring plantaricin NC8, lactacin F and acidocin LF221B. The distribution of bacteriocins in this dataset gives an indication of HGT: LS2 is confined to a single sub-clade and was probably transferred into the megaplasmid of the ancestor of these four strains; MR10B is present on the only small plasmid in three divergent strains.

The production of bacteriocins gives a strain an obvious competitive advantage since it inhibits similar strains and species that may compete strongly for limited resources. Specific environments impose different biotic and abiotic factors and the details of microbial competition and horizontal transfer of genes (including bacteriocin genes) are dependent on a complicated interplay among these factors, potentially explaining the scattered distribution of bacteriocin genes in this dataset.

## Concluding remarks

We conducted a comparative genomic study of 42 strains of *L. salivarius* and a closely related outgroup, *L. hayakitenis* DSM 18933^T^. Results from previous comparative studies indicate that there is considerable functional and phylogenetic diversity across species of the genus *Lactobacillus* species. Smaller scale intra-specific studies focusing on single species of the genus *Lactobacillus* highlight the continuation of this trend across strains.

We demonstrate that *L. salivarius* has an open pan-genome and that all major functional groups described show considerable functional variation across strains, often displaying greater similarity within sub-clusters as opposed to niche-specific trends. Variation in gene function is greater across the megaplasmids than across the chromosomes and greater across the smaller plasmids than across the megaplasmids. The level of functional variation revealed in *L. salivarius* indicates that strain-specific properties might be applied to commercial areas of human health and nutrition such as probiotics and food preservation.

## Data bibliography

1. Harris HMB. Genbank. BioProject ID: PRJNA357984; BioSample accession number: SAMN06163248; GenBank accession number: MSCR00000000 (01M14315)
2. Harris HMB. Genbank. BioProject ID: PRJNA357984; BioSample accession number: SAMN06163249. GenBank accession number: NBEY00000000 (AH4231).
3. Harris HMB. Genbank. BioProject ID: PRJNA357984; BioSample accession number: SAMN06163250. GenBank accession number: NBEX00000000 (AH4331).
4. Harris HMB. Genbank. BioProject ID: PRJNA357984; BioSample accession number: SAMN06163251. GenBank accession number: NBEW00000000 (AH43324).
5. Harris HMB. Genbank. BioProject ID: PRJNA357984; BioSample accession number: SAMN06163252. GenBank accession number: NBEV00000000 (AH43348).
6. Harris HMB. Genbank. BioProject ID: PRJNA357984; BioSample accession number: SAMN06163253. GenBank accession number: NBEU00000000 (CCUG 2753OB).
7. Harris HMB. Genbank. BioProject ID: PRJNA357984; BioSample accession number: SAMN06163254. GenBank accession number: NBET00000000 (CCUG 38008).
8. Harris HMB. Genbank. BioProject ID: PRJNA357984; BioSample accession number: SAMN06163255. GenBank accession number: NBES00000000 (CCUG 44481).
9. Harris HMB. Genbank. BioProject ID: PRJNA357984; BioSample accession number: SAMN06163256. GenBank accession number: NBER00000000 (CCUG 45735).
10. Harris HMB. Genbank. BioProject ID: PRJNA357984; BioSample accession number: SAMN06163257. GenBank accession number: NBEQ00000000 (CCUG 47171).
11. Harris HMB. Genbank. BioProject ID: PRJNA357984; BioSample accession number: SAMN06163258. GenBank accession number: NBEP00000000 (CCUG 47825).
12. Harris HMB. Genbank. BioProject ID: PRJNA357984; BioSample accession number: SAMN06163259. GenBank accession number: NBEO00000000 (CCuG47826).
13. Harris HMB. Genbank. BioProject ID: PRJNA357984; BioSample accession number: SAMN06163260. GenBank accession number: NBEN00000000 (DSM 20492).
14. Harris HMB. Genbank. BioProject ID: PRJNA357984; BioSample accession number: SAMN06163261. GenBank accession number: NBEM00000000 (DSM 20554).
15. Harris HMB. Genbank. BioProject ID: PRJNA357984; BioSample accession number: SAMN06163262. GenBank accession number: NBEL00000000 (gul1).
16. Harris HMB. Genbank. BioProject ID: PRJNA357984; BioSample accession number: SAMN06163263. GenBank accession number: NBEK00000000 (gul2).
17. Harris HMB. Genbank. BioProject ID: PRJNA357984; BioSample accession number: SAMN06163264. GenBank accession number: NBEJ00000000 (JCM 1040).
18. Harris HMB. Genbank. BioProject ID: PRJNA357984; BioSample accession number: SAMN06163265. GenBank accession number: NBEI00000000 (JCM 1042).
19. Harris HMB. Genbank. BioProject ID: PRJNA357984; BioSample accession number: SAMN06163266. GenBank accession number: NBEH00000000 (JCM 1044).
20. Harris HMB. Genbank. BioProject ID: PRJNA357984; BioSample accession number: SAMN06163267. GenBank accession number: NBEG00000000 (JCM 1045).
21. Harris HMB. Genbank. BioProject ID: PRJNA357984; BioSample accession number: SAMN06163268. GenBank accession number: NBEF00000000 (JCM 1047).
22. Harris HMB. Genbank. BioProject ID: PRJNA357984; BioSample accession number: SAMN06163269. GenBank accession number: NBEE00000000 (JCM 1230).
23. Harris HMB. Genbank. BioProject ID: PRJNA357984; BioSample accession number: SAMN06163270. GenBank accession number: NBED00000000 (L21).
24. Harris HMB. Genbank. BioProject ID: PRJNA357984; BioSample accession number: SAMN06163271. GenBank accession number: NBEC00000000 (LMG 14476).
25. Harris HMB. Genbank. BioProject ID: PRJNA357984; BioSample accession number: SAMN06163272. GenBank accession number: NBEB00000000 (LMG 14477).
26. Harris HMB. Genbank. BioProject ID: PRJNA357984; BioSample accession number: SAMN06163273. GenBank accession number: NBEA00000000 (NCIMB 702343).
27. Harris HMB. Genbank. BioProject ID: PRJNA357984; BioSample accession number: SAMN06163274. GenBank accession number: NBDZ00000000 (NCIMB 8816).
28. Harris HMB. Genbank. BioProject ID: PRJNA357984; BioSample accession number: SAMN06163275. GenBank accession number: NBDY00000000 (NCIMB 8817).
29. Harris HMB. Genbank. BioProject ID: PRJNA357984; BioSample accession number: SAMN06163276. GenBank accession number: NBDX00000000 (NCIMB 8818).
30. Harris HMB. dx.doi.org/10.6084/m9.figshare.4577917.v1 (data file 1).
31. Harris HMB. dx.doi.org/10.6084/m9.figshare.4577947.v1 (data file 2).
32. Harris HMB. dx.doi.org/10.6084/m9.figshare.4577950.v1 (data file 3).
33. Harris HMB. dx.doi.org/10.6084/m9.figshare.4577953.v1 (data file 4).
34. Harris HMB. dx.doi.org/10.6084/m9.figshare.4577956.v1 (data file 5).
35. Harris HMB. dx.doi.org/10.6084/m9.figshare.4577965.v1 (data file 6).
36. Harris HMB. dx.doi.org/10.6084/m9.figshare.4577971.v1 (data file 7).
37. Harris HMB. dx.doi.org/10.6084/m9.figshare.4577977.v1 (data file 8).

## Supplementary Data

10780 Supplementary File 1
