# Supplementary material for: Phylogenomics and comparative genomics of Lactobacillus salivarius, a mammalian gut commensal
Source: Microb Genom. 2017 Jun 13;3(8):e000115. doi: 10.1099/mgen.0.000115 (PMC5610712; doi:10.1099/mgen.0.000115)
Supplement: Supplementary File 1 [file mgen-3-115-s001.pdf]

SUPPLEMENTARY TEXT

METHODS

Quality assessment of genomes

Three additional quality control steps were performed on all genomes. First, the genomes were BLASTed (blastn v2.2.26+) [1] against a filtered version of the RDP database (v11.1) [2] that included only complete or near-complete 16S rRNA genes (>1400 bp) annotated to species level. This was carried out to confirm that the top hit for each assembly was an *L. salivarius* sequence in the RDP database and also to make sure that no other good hit (>1000 bp; identity >= 97%) was found, which would indicate possible contamination. Second, 39 universal marker genes [3] were BLASTed (tblastn) against the contigs of each genome. The reasoning here was that all 39 marker genes should be fully assembled in a high-quality genome (which they were). Third, the contigs of each genome were assessed using Kraken (v0.10.6) [4] as a further test for possible contamination. The results of all 43 Kraken runs are shown in Table S4.

Assigning contigs to replicons

A common problem when analysing genes of interest in draft genomes is being able to tell whether a particular gene is present on the chromosome or on a plasmid. Since all of a contig must either be part of the chromosome or part of a plasmid, once a contig has been assigned to a replicon the genes present on the contig can also be assigned to the same replicon. We describe here the method used in this study to assign each contig to its most likely replicon.

A database of 92 plasmids (10 megaplasmids and 82 plasmids) from 16 *Lactobacillus* species was generated from complete genomes available on NCBI. Each draft genome was BLASTed (blastn v2.2.26+) against this database and the results were filtered in order to assign each contig to a plasmid or, failing plasmid assignment, to the chromosome. Megaplasmids (>10 kb; both circular and linear) and smaller plasmids (<10 kb) were included in the database so four categories of replicon were possible (including the chromosome).

To justify BLAST thresholds for assigning contigs to replicon categories, 4 complete genomes (UCC118, CECT5713, Ren and NIAS840) were broken up into contigs using a randomly generated chi-squared distribution with Degrees of Freedom equal to 1. This distribution was chosen because its median Spearman correlation with the distribution of lengths of contigs for each draft genome in our study was 0.96 (Q1 = 0.9; Q3 = 0.98; n = 38), ensuring that the artificial draft genomes would resemble the draft genomes in our dataset in terms of contig length distributions. The values of this distribution were then converted to proportions and randomly permuted in order to avoid a bias between contig length and genome region. Each genome was divided up based on the order of the randomly permuted proportions where each proportion is a fraction of the total number of base pairs in the genome. For each of the 4 complete genomes, each replicon was broken up into 50 contigs and contigs less than 200 bp were excluded. The FASTA header for each contig was labelled with the

replicon from which it was taken so that the specificity and sensitivity of the contig assignment method could be tested. The R (v3.2.3) code uploaded to figshare (Data Bibliography of main text; data file 5) shows the steps for generating 50 draft contigs from the complete chromosome sequence of UCC118.

The 4 artificial draft genomes were then BLASTed (blastn) against the database of 92 plasmids. This was done for each genome separately so that the complete plasmids from each genome being BLASTed could be removed from the database beforehand. This ensured that draft plasmid contigs were not just aligning to the complete version of their own plasmids. An unfiltered evaluation of the BLAST results showed that the highest % alignment length of a chromosomal contig against the plasmid database was 23.7% (490/2,070). An alignment length of 25% against the plasmid database was therefore chosen as the cut-off for assigning contigs to plasmids. BLAST hits between two sequences can have multiple high-scoring pairs (HSPs) so the sum of the non-overlapping length of all HSPs between each contig and reference plasmid was calculated. The reference plasmid sequence with the highest % alignment to the contig was chosen and all alignments of less than 25% were excluded. Depending on their top hit, these remaining contigs were assigned to one of three categories: plasmid, circular megaplasmid or linear megaplasmid. It should be noted that small contigs representing transposases or other small repetitive regions may be present on both the chromosome and the plasmid(s) so the assignment of these contigs is less reliable. The sensitivity and specificity of the BLAST results for the 4 artificial draft genomes against the plasmid database are shown in Table S5. Code for calculating the sum of the non-overlapping length of HSPs between each contig and each reference plasmid has been uploaded to figshare (Data Bibliography of main text; data file 6).

As an additional quality check, three genes identified as being specific to the *L. salivarius* circular megaplasmid(5) - *repA* (LSL_1739), *repE* (LSL_1740) and *parA* (LSL_1741) – were BLASTed (tblastn) against the contigs for each genome assembly to see if all top hits were to predicted megaplasmid contigs. Results are in Table S2.

Specific functional groups

COG categories for genes were predicted by BLASTing (blastp) amino acid sequences against a COG database (ftp://ftp.ncbi.nih.gov/pub/COG/COG2014/data) with thresholds of 40% identity, 50% alignment length of the query gene and a BLAST bit score of 60. Any gene match that fell below these thresholds was added to the COG category 'unknown function'. For each genome, genes were assigned to their respective replicons.

Peptidases were predicted by BLASTing (blastp) amino acid sequences against full sequences from the MEROPS database (https://merops.sanger.ac.uk). BLAST thresholds used were 40% identity, 50% alignment length of the query gene and a BLAST bit score of 60.

Sortase genes were predicted using hmmscan from the HMMER3 (v3.1b1) [6] toolkit with the following downloaded sortase family HMM profiles: http://nihserver.mbi.ucla.edu/Sortase/sortase_family_classification.hmm. A cut-off score of >30 was chosen to balance false positive and false negative predictions based on comparisons with the non-redundant NCBI and KEGG annotations. Genes with an LPXTG motif were predicted using hmmscan with a TIGRFAM [7] HMM profile (TIGR01167) and an e-value cut-off of 1e-05. The LOCP [8] webserver was used to locate putative pilus operons using default parameters. All three methods used amino acid sequences as input.

Glycosyl hydrolases and glycosyl transferases were predicted using hmmscan with DBcan [9] HMM profiles (http://csbl.bmb.uga.edu/dbCAN/). For each genome, GH and GT genes were assigned

to their respective replicons. A cut-off score of >30 was chosen to balance false positive and false negative predictions based on comparisons with non-redundant NCBI and KEGG annotation.

The Bagel3 [10] webserver was used to predict genetic loci for bacteriocin production and surrounding areas of interest (AOIs) using marker genes. For each genome, AOIs were sorted into their respective replicons.

CRISPRs were predicted using MinCED (v0.2.0), which was downloaded from the following link: https://github.com/ctSkennerton/minced. To predict cas genes associated with each CRISPR, hmmscan was used with cas-specific HMMs from TIGRFAM. CRISPRs that had no associated cas genes were labelled as 'undefined'. The same method used to build the core-gene phylogenetic tree was also used to build a tree from the amino acid sequences of the cas 1 (type-II and type-III) gene.

Genes involved in exopolysaccharide biosynthesis were predicted using the two EPS clusters of UCC118 as references. This was the only functional group that relied on a reference genome and it was used in order to give an overview of EPS genetic diversity in *L. salivarius* since a much larger, more detailed study is being conducted on the intra-specific diversity and functionality of EPS clusters in *L. salivarius* (Bourin *et al*; in preparation). Amino acid sequences of the UCC118 EPS genes were BLASTed (tblastn) against contigs with thresholds of 40% identity, 50% alignment length of the query gene and a BLAST bit score of 60. BLAST hits were then manually curated, taking note of UCC118 EPS genes present in multiple copies (in the case of transposases) and genes that passed the thresholds but were located in very different regions of the genome than the other predicted EPS genes.

Signal peptides were predicted using SignalP (v4.1) [11] with default parameters for gram-positive bacteria. Transmembrane domains were predicted using TMHMM (v2.0) [12] and all predictions with more than 10 expected amino acids in transmembrane helices in the first 60 amino acids were excluded from the results due to their likelihood of being signal peptides (see Instructions at http://www.cbs.dtu.dk/services/TMHMM/).

Antibiotic resistance (AR) genes were predicted using an AR reference gene set from the Comprehensive Antibiotic Resistance Database (CARD; v1.09) [13]. Within the CARD database, the FASTA file denoted 'protein homolog model' was filtered to include only complete genes and then genes were translated from nucleotide to amino acid sequences. Amino acid sequences for each genome were BLASTed against this database and filtered at 40% identity, 50% alignment length of the query gene and a BLAST score of 60.

Potential virulence factors (VF) were predicted using a version of the virulence factor database (VFDB) [14], which was downloaded from the following link: http://www.mgc.ac.cn/VFs/Down/VFDB_setA_pro.fas.gz. This database is the core dataset and contains virulence factor genes that have been experimentally verified only - the full database was not used in order to minimise the number of false positive gene predictions. Amino acid sequences for each genome were BLASTed against the database and filtered at 70% identity and 90% alignment length of the query gene. More stringent cut-off values were used for virulence factors compared with antibiotic resistance genes because using BLAST to identify homologous genes based on the VF database is known to produce false positives at lower cut-off values.

Prophages were predicted using VIRSorter (v1.0.2) [15] where predicted regions with the lowest confidence (category 3; 'not so sure') for both complete phage contigs and prophages were excluded. Predicted phage genes for the remaining categories were assigned to COG categories (the same COG database used for the general COG analysis) using blastp with thresholds of 40% identity, 50% alignment length of the query gene and a BLAST bit score of 60. Any gene that fell below these thresholds was added to the COG category 'unknown function'.

Transposases were predicted using hmmscan with TnpPred [16] HMM profiles downloaded from the following link: http://www.mobilomics.cl/. An e-value cut-off of 1e-05 was used. For each genome, predicted transposases were assigned to their respective replicons.

Bile salt hydrolase genes were predicted using a subset of the KEGG database where the EC number 3.5.1.24 was used to select bile salt hydrolase genes. Amino acid sequences were BLASTed (blastp) against this database with thresholds of 40% identity, 50% alignment length of the query (and reference) gene and a BLAST bit score of 60. These genes were then BLASTed against each other to give a pairwise BLAST score for each pair of bile salt hydrolase genes.

All statistics and data visualisation were carried out in R (v3.2.3) [17]. R packages used during this study were MADE4 [18] and SeqinR [19].

RESULTS AND DISCUSSION

The core-gene phylogenetic tree of *L. salivarius* has similar sub-clade topology to POCP clusters, but overall tree topology is dissimilar

Percentage of Conserved Proteins (POCP) [20] calculates a similarity score based on percentage of genes in common between all the amino acid sequences in two genomes. POCP was designed as a method to identify whether a particular species belongs within a genus. We were not interested in applying this threshold since all strains obviously fall within a single genus; instead, the goal was to assess the congruency of a core-gene phylogeny with a method that clustered the strains based on the presence and absence of genes. Fig. S3 shows a heatmap of POCP values, where clustering of strains is in reasonable agreement with the core-gene phylogeny of Fig. 2 in terms of sub-clades. Several strains cluster apart from their core-gene sub-clades including CECT5713 and CCUG38008. A greater difference between POCP and the core-gene tree versus ANI and the core-gene tree is expected because POCP value calculations ignore homologous regions, using similarity based on gene presence and absence distributions to cluster strains. This is a rough approximation of the combined effect of gene decay and HGT since a gene that is present in one strain and absent in another has either acquired a deleterious mutation or else has been horizontally transferred by one of several mechanisms. The reason why many of the sub-clades in Fig. S3 agree with the core-gene phylogeny is that the probability of gene decay or HGT events having occurred after two strains start to diverge from a common ancestor increases with time. Adaptation to different niches and differing selection pressures then start to disrupt the correlation between core-gene phylogeny and clustering of shared/unshared genes [21]. We found no general association of clusters from any tree generated in this study with the isolation sources of the strains (Table S1), but members of several small clusters were all isolated from the same source. This overall lack of niche-strain association may be due to the transient appearance of *L. salivarius* in niches associated with the gastro-intestinal tract (food, opportunistic infection of body sites, etc.) and it would be a mistake to assume that every strain has acquired niche-specific adaptations to its source of primary isolation.

Protease genes show no variation or considerable variation depending on MEROPS protease family

Proteases are a large group of proteins, divided into many families that are involved in the hydrolysis of peptides. Fig. S4 shows 53 protease families that display variation across the 43

genomes in this dataset. Genes for eighteen additional protease families were predicted in *L. salivarius*, but these families showed no variation across the strains, with 17 represented by a single gene per strain (A01A, C108, C14B, C19, C46, I04, I87, M02, M10A, M13, M15D, M20B, M20D, M24A, S09A, S09B, T05, T06) and one, a cysteine protease (C19) described as 'ubiquitin-specific' by the MEROPS database (Table S6), represented by two genes per strain. It can be speculated that these 18 families are subjected to purifying selection since the remaining 53 protease families vary both in gene count and in presence and absence across the strains.

Out of the 53 protease families that vary in their distributions, 33 are present in all 43 genomes, but have variable gene counts; genes for thirty of these are found on the chromosome only while the remaining three are present on multiple replicons. The gene count per protease family ranges from 0 to 24 where some families are present in all but a single genome and other families are present in one only (usually DSM18933 – the strain of *L. hayakitensis* used in this study). The protease family with the most genes in *L. salivarius* (4-24) is M23B, which is annotated as a lysostaphin in the MEROPS database (Table S6), an antibacterial enzyme that degrades peptidoglycan in the cell walls of certain bacteria, staphylococci in particular.

There are a number of protease families and protease inhibitors that are rare in the dataset of *L. salivarius* annotations, with representatives belonging to one or several genomes only. JCM1046 has gene products in two families that the other strains do not have – I75 and S26B – both relevant genes predicted to reside on the chromosome. The gene encoding I75 is on a small contig of 964 bp that has a 99% match over its full length to a phage from *E. coli*, suggesting recent acquisition of this sequence as a prophage. The only other predicted protease inhibitor, I63, is an inhibitor of pappalysin-1 and it is present in all *L. salivarius* genomes but absent from *L. hayakitensis* DSM18933. S26B is a signal peptidase that cleaves signal peptides from a secreted protein as it is being translated. DSM18933 has 2 protease families that are not present in *L. salivarius*, which suggests that they were either horizontally acquired by *L. hayakitensis* after the split from its common ancestor with *L. salivarius* or else that *L. salivarius* subsequently lost these families through gene decay, whether through genetic drift or active selection pressure. These two families are M42 and M60, a glutamyl aminopeptidase and an enhancin, respectively.

Sun *et al* conducted a genus-wide, comparative genomic study of lactobacilli and found considerable variation in cell-envelope proteases [22]. Our study shows that a more general overview of protease families reflects the high levels of variation seen in *Lactobacillus*, at the species level, in *L. salivarius*.

Prophages, CRISPRs and insertion sequences are widely distributed across *L. salivarius* but no obvious association exists between them

Two agents of HGT that affect both the bacterial chromosome and extrachromosomal replicons are bacteriophages and insertion sequences (consisting primarily of a transposase gene). Bacteriophages are ubiquitous among bacterial communities and phage-host dynamics has been shown to stabilise diversity within a community [23] as well as to drive the arms race between the evolution of bacterial defences (often in the form of CRISPR-cas systems) and the counter-evolution of phage structures that neutralise those defences [24]. Insertion sequences (IS) have been implicated in the horizontal transfer of a wide range of functions and are noted for their role in conferring niche-specific advantages to bacteria, allowing the persistence of strains or species in new environments that were previously uninhabitable [25].

Fig. S5 shows a heatmap of predicted prophage genes (COGs) and Fig. S6 shows a barplot of prophage counts. Nine strains (2 sub-clades of 4 strains each and JCM1230) lack predicted

prophages; it is unlikely that these 9 strains have no history of interacting with bacteriophages - instead, VirSorter has failed to predict relatively intact prophages in the genomes of these strains. Canchaya *et al* summarise the relationship between bacteria and prophages by writing that prophages are lost from bacterial genomes as easily as they are acquired [26]. There is no clear association of the two sub-clades with a single niche, although the human oral cavity is the isolation source of 5 of these strains and the other 4 were isolated from the mammalian intestine. It is tempting to suggest that the oral environment selects against the persistence of prophages; however, Edlund *et al* describe the oral cavity as the perfect portal for viruses to access the oral microbial community [27] and previous studies have shown that it is host to a diverse community of phages [28, 29].

The COG category in Fig. S5 with by far the most genes is 'Function unknown' (S) with a mean average gene count of 61.3 compared with the second highest - 'General function prediction only' - of 3. The size of these categories emphasises the limits of current knowledge regarding bacteriophage gene function. There is a correlation between number of predicted prophages and number of prophage genes (Spearman; rho = 0.78; $p < 0.001$), which is largely expected and highlights the size constraints on phages that infect *L. salivarius* since number of prophages, not phage type, approximately accounts for number of prophage genes. Some of the COG categories that are least abundant in predicted prophages are those involved in cell-specific functions such as cell motility (N) and secretion (U). The distribution of the remaining COG categories across the strains is indicative of the dynamic nature between bacteria and their phages, with considerable intra-species variation suggesting that the prophage complement of the ancestor of *L. salivarius* does not resemble any of the currently extant strains since their repertoire of prophages is so distinct.

Table S7 describes the distribution of CRISPRs across the 43 genomes as well as their associated cas genes. All CRISPRs are located on the chromosome, highlighting their role in protecting against extrachromosomal sequences. Almost all strains in this dataset have either the type-II or type-III CRISPR-cas system (or both), identified by the cas 9 or cas 10 gene, respectively, and 6 strains have no identified CRISPRs. The presence of either type-II or type-III CRISPR-cas systems show some clustering on the core-gene tree in Fig. 2: the DSM20555^{T} sub-clade consisting of 4 strains all have the type-III system only while the CECT5713 (6 strains) and UCC118 (4 strains) sub-clades have the type-II system only; the AH43348 sub-clade (6 strains), in contrast, has both type-II and type-III systems. The partial clustering of CRISPR-cas systems according to the core-gene tree is supported by Fig. S7, which shows a maximum-likelihood tree of the cas 1 gene for type-II CRISPR-cas, providing evidence of CRISPR-cas systems being acquired and maintained in the common ancestors of these sub-clades. The 6 strains with no CRISPRs show some clustering on the core-gene tree in Fig. 2, but JCM1045 and DSM18933 are singletons. The absence of CRISPR-cas systems does not have an obvious association with niche or the presence of prophages, suggesting that the interaction between CRISPR-cas systems, bacteriophages and the environment is far from straightforward. There are also 6 undefined CRISPRs from 4 strains that could not be described due to the absence of cas genes in close proximity. These CRISPRs are probably degraded systems that are no longer functional since all functioning CRISPR-cas systems have the cas 1 gene, which is involved in recognition and cleavage of invading DNA.

Fig. S8 shows a heatmap of gene counts for insertion sequences across the 43 strains, divided up into their respective replicons. The most striking thing about this figure is the inter-strain diversity of transposases, both within and between replicons. The gene counts for each transposase family in a specific strain on a particular replicon range from 0 to 52, highlighting the considerable variation in copy number displayed by these horizontally transferred sequences. The majority of transposases have copies on the chromosome and the plasmids, suggesting that they utilise the conjugative ability of plasmids to increase their abundance within and between species. The distributions of the IS families follow different patterns, from being widely spread over all three replicon groups (IS3) to being

limited to the chromosome and megaplasmid (IS21) to being confined to the smaller plasmid(s) (IS256). The only IS family confined to the chromosome is IS1 in a single strain - NIAS840.

The multi-replicon distribution of IS families implies that there is strong selection pressure on insertion sequences to transpose regularly from chromosomes to plasmids and vice versa, perhaps being partly responsible for the fact that transposases are currently considered to account for the most abundant gene families in both prokaryotes and eukaryotes [30]. The widespread distribution of IS3 in *L. salivarius* replicons is mirrored by its abundance (539 genes across the 43 strains); it is also the only family to consist of two sub-families - IS3 and IS150. Similarly, the other IS families with the widest distributions - ISL3, IS21 and IS200 - also have the greatest abundances after IS3, although IS21 is absent from the smaller plasmids even if it is ubiquitous on the *L. salivarius* chromosome.

Overall, IS families with a higher copy number in this dataset show a strong correlation with how many strains (and replicons per strain) harbour them (Spearman; rho = 0.95; $p < 0.001$), showing that insertion sequences do not have a tendency to just replicate within a single replicon without undergoing regular HGT. Out of the 19 IS families present in the TnpPred database (http://www.mobilomics.cl/), 14 are identified in *L. salivarius* in this study. This emphasises the ability of transposases to transfer themselves within and between species, leading to greater sequence diversity and, when they carry additional genes with them, greater functional diversity as well.

Protein secretion and membrane-anchoring gene richness are not associated with strain isolation source

Fig. S9 shows a barplot with the number of genes containing signal peptides and trans-membrane domains in the 43 strains. Proteins belonging to these two functional groups play an important role in the interaction of a bacterium with its environment since they are either secreted from the cell or function as membrane-bound structures. The number of predicted genes with signal peptides and with trans-membrane domains range from 56 to 84 and from 33 to 54, respectively. There is no association between niche and the number of either of these functional groups, which is not entirely surprising. These results highlight once more the point made earlier that certain isolation sources of *L. salivarius* strains shouldn't be interpreted as the niches that each strain has adapted to over time - some strains might be acting as opportunists that don't persist in a given environment for long such as the *Lactobacillus* species from a 2007 study (mainly *L. rhamnosus*) that were isolated from the blood, cerebrospinal fluid, peritoneal fluid and intestinal fistula of immuno-compromised children [31].

Most *L. salivarius* strains harbour genes for two bile salt hydrolases

Fig. S10 shows a heatmap of BLAST scores for all the predicted bile salt hydrolase (Bsh) genes in the 42 *L. salivarius* strains. The ability to hydrolyse bile salts is a necessary trait for any bacterium that is adapted to traversing the initial sections of the gastro-intestinal tract in order to colonise the intestine. It is also a required function for probiotics since a potential probiotic without the ability to reach its target area (usually the colon) will be ineffective. All 42 *L. salivarius* strains have at least one Bsh gene while *L. hayakitensis* DSM18933 has none, suggesting that the common ancestor of *L. salivarius* and *L. hayakitensis* did not possess a Bsh gene, although it is possible that another strain of *L. hayakitensis* does harbour one or more; if this is the case then gene decay of the Bsh gene in DSM18933 is a likely explanation. Two Bsh genes - one on the chromosome and one on the megaplasmid - seems to be the typical organisation in *L. salivarius* as described by Claesson *et al*

[32] since 36 out of 42 strains fit this description. Four strains - CECT5713, JCM1230, LMG14476 and LMG14477 - have a single Bsh gene located on the chromosome while 2 strains - cp400 and JCM1046 - have three BSH genes, both having two on the megaplasmid and one on the chromosome.

The presence of at least one Bsh in all 42 *L. salivarius* strains reinforces the point that this species is commonly isolated from the GIT of humans and animals. The variable number of Bsh genes and their presence on both the chromosome and the megaplasmid suggests that there is variability in bile resistance across the strains. This was shown in a study by Fang *et al*, but they cautioned that bile resistance is independent of the bsh1 allele type (the Bsh on the megaplasmid of most strains) and they go on to show that, upon exposure to bile and cholate, a transcriptome analysis reveals the up-regulation of numerous stress response and efflux proteins, which might mask the variable influence of Bsh allele types [33].

It should be noted that for the BLAST analysis of this category, a stricter cut-off value of 50% for coverage of both the query and reference genes was used. This was done because the number of BLAST hits to Bsh genes in the database contradicted previous literature so a closer agreement in protein length between query and reference sequences was enforced. It is possible that large discrepancies between the lengths of sequences in the database and sequences in the predicted *L. salivarius* gene repertoire led to false negative Bsh predictions. When the criteria are relaxed to include only 50% coverage of the query gene (and not the reference) an extra Bsh is predicted in some strains and these might actually be genuine Bsh genes that this study has excluded.

Summary survey of virulence factors and antibiotic resistance genes

Fig. S11 shows a barplot of the predicted number of putative antibiotic resistance genes (AR) and virulence factors (VF) across the 43 strains. VFs range from 2 to 3 genes and ARs range from 7 to 16. Virulence and antibiotic resistance are two traits that are screened for when assessing the suitability of a strain to act as a probiotic [34] and these traits are particularly dangerous in clinical settings. Table S8 and Table S9 give a more detailed summary of these results for ARs and VFs, respectively, while data file 7 and data file 8 give the corresponding amino acid sequences in FASTA format (figshare; Data Bibliography of main text).

The most commonly predicted function for AR genes in this dataset is transport, specifically a subset of efflux pumps for such antibiotics as tetracycline, elfamycin, bacitracin, clindamycin, fosfomycin, dalfopristin and others. Efflux pumps evolved long before the advent of antibiotic usage in modern medicine and probably originated as a defence against toxic substances entering the cell [35] – a strategy that has more recently been used to confer antibiotic resistance to microbes from multiple drugs, leading to a health crisis in the effective treatment of infection with antibiotics.

Virulence factor identification depends very much on context; a probiotic trait in one setting can be labelled as a virulence factor in another - for instance, when a pathogen acquires the ability to survive intestinal transit in order to colonise the human colon. The most commonly predicted functions for VF genes in our dataset are for an ATP-dependent protease and a UDP-glucose pyrophosphorylase. Overall, there is a wide variety of functions for these potential VFs, both in the VF database and in the predicted functions for *L. salivarius*. This highlights the ongoing evolutionary competition between hosts and microbes, the defensive and counter-defensive adaptive traits that arise from unrelated proteins with an overlapping strategy – to evade host mechanisms and successfully colonise the host environment.

REFERENCES

1. **Altschul SF, Gish W, Miller W, Myers EW, Lipman DJ**. Basic local alignment search tool. *Journal of molecular biology*. 1990 Oct 5;215(3):403-10. PubMed PMID: 2231712. Epub 1990/10/05. eng.
2. **Olsen GJ, Overbeek R, Larsen N, Marsh TL, McCaughey MJ, Maciukenas MA, *et al***. The Ribosomal Database Project. *Nucleic acids research*. 1992 May 11;20 Suppl:2199-200. PubMed PMID: 1598241. Pubmed Central PMCID: PMC333993. Epub 1992/05/11. eng.
3. **Wu D, Jospin G, Eisen JA**. Systematic Identification of Gene Families for Use as "Markers" for Phylogenetic and Phylogeny-Driven Ecological Studies of Bacteria and Archaea and Their Major Subgroups. *PloS one*. 2013;8(10):e77033.
4. **Wood DE, Salzberg SL**. Kraken: ultrafast metagenomic sequence classification using exact alignments. *Genome Biology*. 2014;15(3):R46.
5. **Li Y, Canchaya C, Fang F, Raftis E, Ryan KA, van Pijkeren JP, *et al***. Distribution of megaplasmids in *Lactobacillus salivarius* and other lactobacilli. *Journal of bacteriology*. 2007 Sep;189(17):6128-39. PubMed PMID: 17586640. Pubmed Central PMCID: PMC1951925. Epub 2007/06/26. eng.
6. **Finn RD, Clements J, Eddy SR**. HMMER web server: interactive sequence similarity searching. *Nucleic acids research*. 2011 Jul 1;39(Web Server issue):W29-37. PubMed PMID: 21593126.
7. **Haft DH, Selengut JD, White O**. The TIGRFAMs database of protein families. *Nucleic acids research*. 2003 Jan 1;31(1):371-3. PubMed PMID: 12520025.
8. **Plyusnin I, Holm L, Kankainen M**. LOCP—locating pilus operons in Gram-positive bacteria. *Bioinformatics* (Oxford, England). 2009 May 1, 2009;25(9):1187-8.
9. **Yin Y, Mao X, Yang J, Chen X, Mao F, Xu Y**. dbCAN: a web resource for automated carbohydrate-active enzyme annotation. *Nucleic acids research*. 2012 Jul;40(Web Server issue):W445-51. PubMed PMID: 22645317.
10. **van Heel AJ, de Jong A, Montalban-Lopez M, Kok J, Kuipers OP**. BAGEL3: Automated identification of genes encoding bacteriocins and (non-)bactericidal posttranslationally modified peptides. *Nucleic acids research*. 2013 Jul;41(Web Server issue):W448-53. PubMed PMID: 23677608. Pubmed Central PMCID: PMC3692055. Epub 2013/05/17. eng.
11. **Nielsen H, Engelbrecht J, Brunak S, von Heijne G**. Identification of prokaryotic and eukaryotic signal peptides and prediction of their cleavage sites. *Protein Engineering*. 1997 January 1, 1997;10(1):1-6.
12. **Krogh A, Larsson B, von Heijne G, Sonnhammer EL**. Predicting transmembrane protein topology with a hidden Markov model: application to complete genomes. *Journal of molecular biology*. 2001 Jan 19;305(3):567-80. PubMed PMID: 11152613. Epub 2001/01/12. eng.
13. **McArthur AG, Waglechner N, Nizam F, Yan A, Azad MA, Baylay AJ, *et al***. The Comprehensive Antibiotic Resistance Database. *Antimicrobial Agents and Chemotherapy*. 2013 July 1, 2013;57(7):3348-57.
14. **Chen L, Yang J, Yu J, Yao Z, Sun L, Shen Y, *et al***. VFDB: a reference database for bacterial virulence factors. *Nucleic acids research*. 2005 Jan 1;33(Database Issue):D325-8. PubMed PMID: 15608208.
15. **Roux S, Enault F, Hurwitz BL, Sullivan MB**. VirSorter: mining viral signal from microbial genomic data. *PeerJ*. 2015;3:e985. PubMed PMID: 26038737. Pubmed Central PMCID: PMC4451026. Epub 2015/06/04. eng.
16. **Riadi G, Medina-Moenne C, Holmes DS**. TnpPred: A Web Service for the Robust Prediction of Prokaryotic Transposases. *Comp Funct Genomics*. 2012;2012:5.
17. **R Core Team. R**: A language and environment for statistical computing. R Foundation for Statistical Computing, Vienna, Austria. URL https://www.R-project.org/. 2015.
18. **Culhane AC, Thioulouse J, Perriere G, Higgins DG**. MADE4: an R package for multivariate analysis of gene expression data. *Bioinformatics* (Oxford, England). 2005 Jun 1;21(11):2789-90. PubMed PMID: 15797915. Epub 2005/03/31. eng.

19. **Charif D, Lobry JR**. SeqinR 1.0-2: A Contributed Package to the R Project for Statistical Computing Devoted to Biological Sequences Retrieval and Analysis. In: Bastolla U, Porto M, Roman HE, Vendruscolo M, editors. Structural Approaches to Sequence Evolution: Molecules, Networks, Populations. Berlin, Heidelberg: Springer Berlin Heidelberg; 2007. p. 207-32.
20. **Qin QL, Xie BB, Zhang XY, Chen XL, Zhou BC, Zhou J, *et al***. A proposed genus boundary for the prokaryotes based on genomic insights. *Journal of bacteriology*. 2014 Jun;196(12):2210-5. PubMed PMID: 24706738. Pubmed Central PMCID: PMC4054180. Epub 2014/04/08. eng.
21. **Winker K**. Reuniting Phenotype and Genotype in Biodiversity Research. *BioScience*. 2009 September 1, 2009;59(8):657-65.
22. **Sun Z, Harris HM, McCann A, Guo C, Argimon S, Zhang W, *et al***. Expanding the biotechnology potential of lactobacilli through comparative genomics of 213 strains and associated genera. *Nature commun*. 2015;6:8322. PubMed PMID: 26415554. Pubmed Central PMCID: PMC4667430. Epub 2015/09/30. eng.
23. **Rodriguez-Valera F, Martin-Cuadrado A-B, Rodriguez-Brito B, Pasic L, Thingstad TF, Rohwer F, *et al***. Explaining microbial population genomics through phage predation. *Nat Rev Micro*. 2009 11//print;7(11):828-36.
24. **Horvath P, Barrangou R**. CRISPR/Cas, the immune system of bacteria and archaea. *Science* (New York, NY). 2010 Jan 8;327(5962):167-70. PubMed PMID: 20056882. Epub 2010/01/09. eng.
25. **Ochman H, Lawrence JG, Groisman EA**. Lateral gene transfer and the nature of bacterial innovation. *Nature*. 2000 05/18/print;405(6784):299-304.
26. **Canchaya C, Fournous G, Brussow H**. The impact of prophages on bacterial chromosomes. *Molecular microbiology*. 2004 Jul;53(1):9-18. PubMed PMID: 15225299. Epub 2004/07/01. eng.
27. **Edlund A, Santiago-Rodriguez TM, Boehm TK, Pride DT**. Bacteriophage and their potential roles in the human oral cavity. *J Oral Microbiol*. 2015;7:27423. PubMed PMID: 25861745. Pubmed Central PMCID: PMC4393417. Epub 2015/04/12. eng.
28. **Hitch G, Pratten J, Taylor PW**. Isolation of bacteriophages from the oral cavity. *Lett Appl Microbiol*. 2004;39(2):215-9. PubMed PMID: 15242464. Epub 2004/07/10. eng.
29. **Pride DT, Salzman J, Haynes M, Rohwer F, Davis-Long C, White RA, III, *et al***. Evidence of a robust resident bacteriophage population revealed through analysis of the human salivary virome. *ISME J*. 2012 05//print;6(5):915-26.
30. **Aziz RK, Breitbart M, Edwards RA**. Transposases are the most abundant, most ubiquitous genes in nature. *Nucleic acids research*. 2010 Jul;38(13):4207-17. PubMed PMID: 20215432. Pubmed Central PMCID: PMC2910039. Epub 2010/03/11. eng.
31. **Muszynski Z, Mirska I, Matuska K**. [*Lactobacillus* species as opportunistic pathogens in children]. *Przeglad epidemiologiczny*. 2007;61(1):79-84. PubMed PMID: 17702443. Epub 2007/08/19. Paleczki z rodzaju *Lactobacillus*--czynnik zakazen oportunistycznych u dzieci. pol.
32. **Claesson MJ, Li Y, Leahy S, Canchaya C, van Pijkeren JP, Cerdeno-Tarraga AM, *et al***. Multireplicon genome architecture of *Lactobacillus salivarius*. *Proc Natl Acad Sci USA*. 2006 Apr 25;103(17):6718-23. PubMed PMID: 16617113. Pubmed Central PMCID: PMC1436024. Epub 2006/04/18. eng.
33. **Fang F, Li Y, Bumann M, Raftis EJ, Casey PG, Cooney JC, *et al***. Allelic variation of bile salt hydrolase genes in *Lactobacillus salivarius* does not determine bile resistance levels. *Journal of bacteriology*. 2009 Sep;191(18):5743-57. PubMed PMID: 19592587. Pubmed Central PMCID: PMC2737978. Epub 2009/07/14. Eng.
34. **Bennedsen M, Stuer-Lauridsen B, Danielsen M, Johansen E**. Screening for Antimicrobial Resistance Genes and Virulence Factors via Genome Sequencing. *Appl Environ Microbiol*. 2011 Apr;77(8):2785-7. PubMed PMID: 21335393.
35. **Webber MA, Piddock LJV**. The importance of efflux pumps in bacterial antibiotic resistance. *J Antimicrob Chemother*. 2003 January 1, 2003;51(1):9-11.

Fig. S1

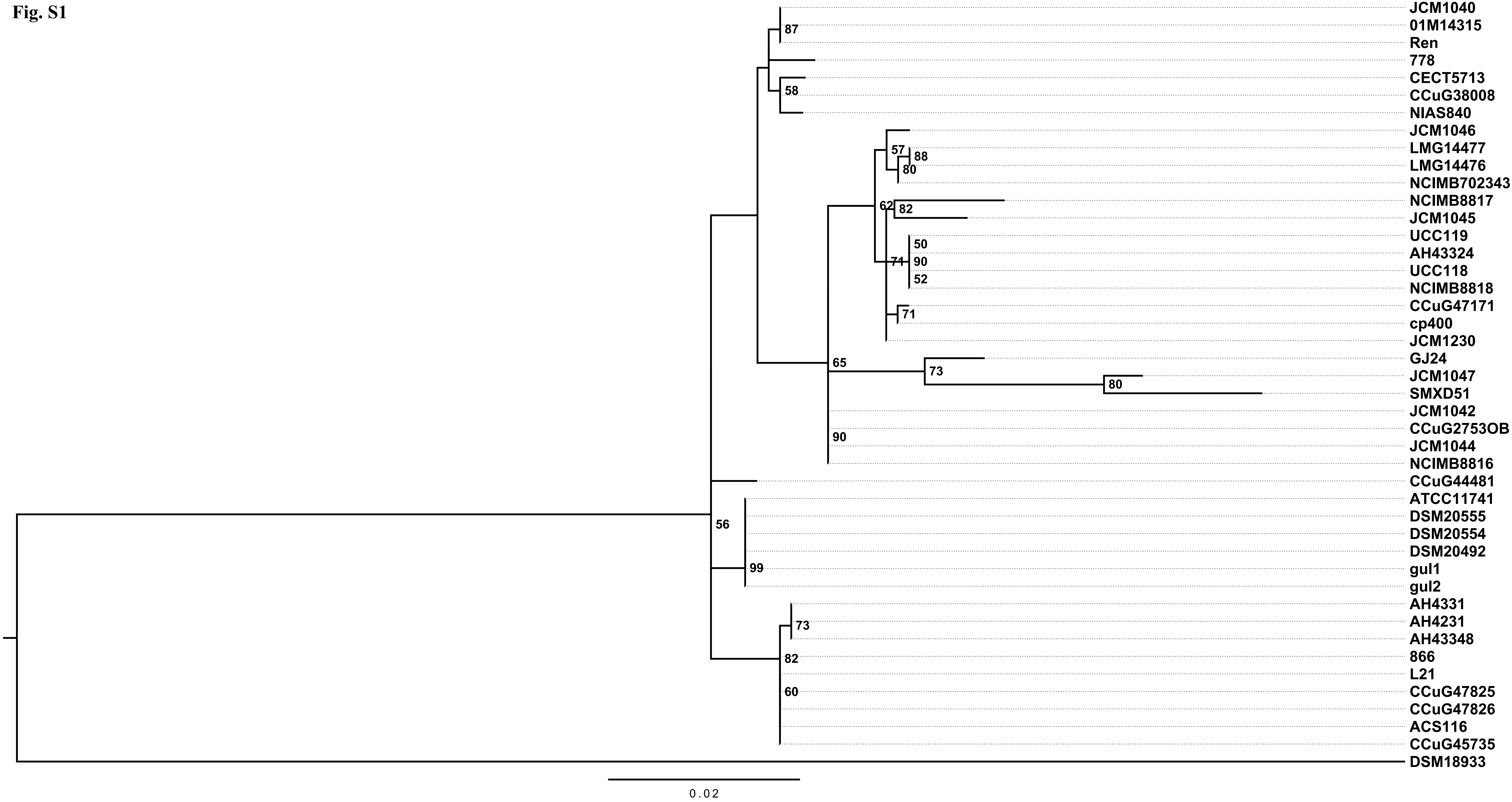
JCM1040
01M14315
Ren
778
CECT5713
CCuG38008
NIAS840
JCM1046
LMG14477
LMG14476
NCIMB702343
NCIMB8817
JCM1045
UCC119
AH43324
UCC118
NCIMB8818
CCuG47171
cp400
JCM1230
GJ24
JCM1047
SMXD51
JCM1042
CCuG2753OB
JCM1044
NCIMB8816
CCuG44481
ATCC11741
DSM20555
DSM20554
DSM20492
gul1
gul2
AH4331
AH4231
AH43348
866
L21
CCuG47825
CCuG47826
ACS116
CCuG45735
DSM18933
87
58
57
88
80
62
82
50
71
90
52
71
65
73
80
90
56
99
73
82
60
0.02

Fig. S2

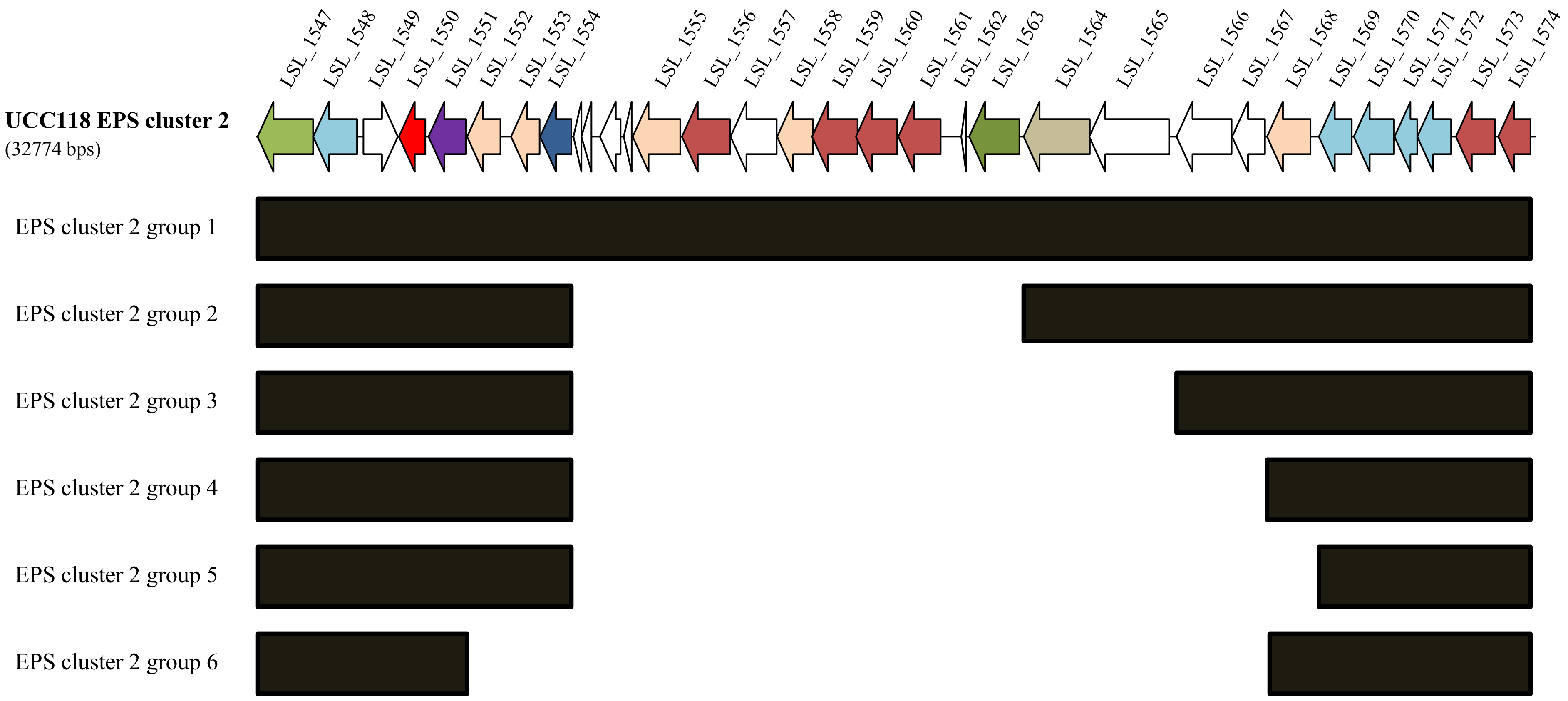

LSL_1547
LSL_1548
LSL_1549
LSL_1550
LSL_1551
LSL_1552
LSL_1553
LSL_1554
LSL_1555
LSL_1556
LSL_1557
LSL_1558
LSL_1559
LSL_1560
LSL_1561
LSL_1562
LSL_1563
LSL_1564
LSL_1565
LSL_1566
LSL_1567
LSL_1568
LSL_1569
LSL_1570
LSL_1571
LSL_1572
LSL_1573
LSL_1574
UCC118 EPS cluster 2
(32774 bps)
EPS cluster 2 group 1
EPS cluster 2 group 2
EPS cluster 2 group 3
EPS cluster 2 group 4
EPS cluster 2 group 5
EPS cluster 2 group 6

Fig. S3

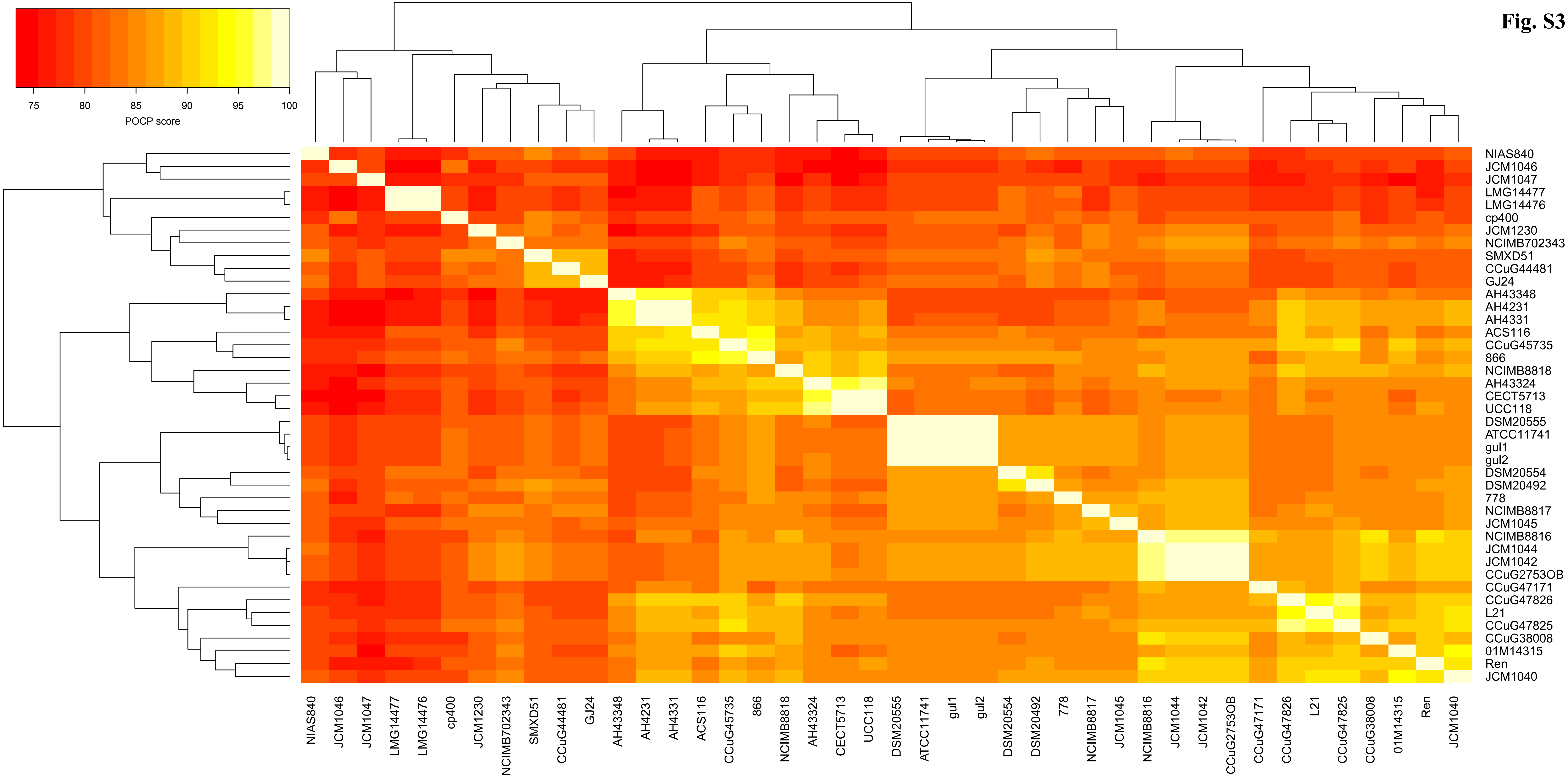

75
80
85
90
95
100
POCP score
NIAS840
JCM1046
JCM1047
LMG14477
LMG14476
cp400
JCM1230
NCIMB702343
SMXD51
CCuG44481
GJ24
AH43348
AH4231
AH4331
ACS116
CCuG45735
866
NCIMB8818
AH43324
CECT5713
UCC118
DSM20555
ATCC11741
gul1
gul2
DSM20554
DSM20492
778
NCIMB8817
JCM1045
NCIMB8816
JCM1044
JCM1042
CCuG2753OB
CCuG47171
CCuG47826
L21
CCuG47825
CCuG38008
01M14315
Ren
JCM1040

Fig. S4

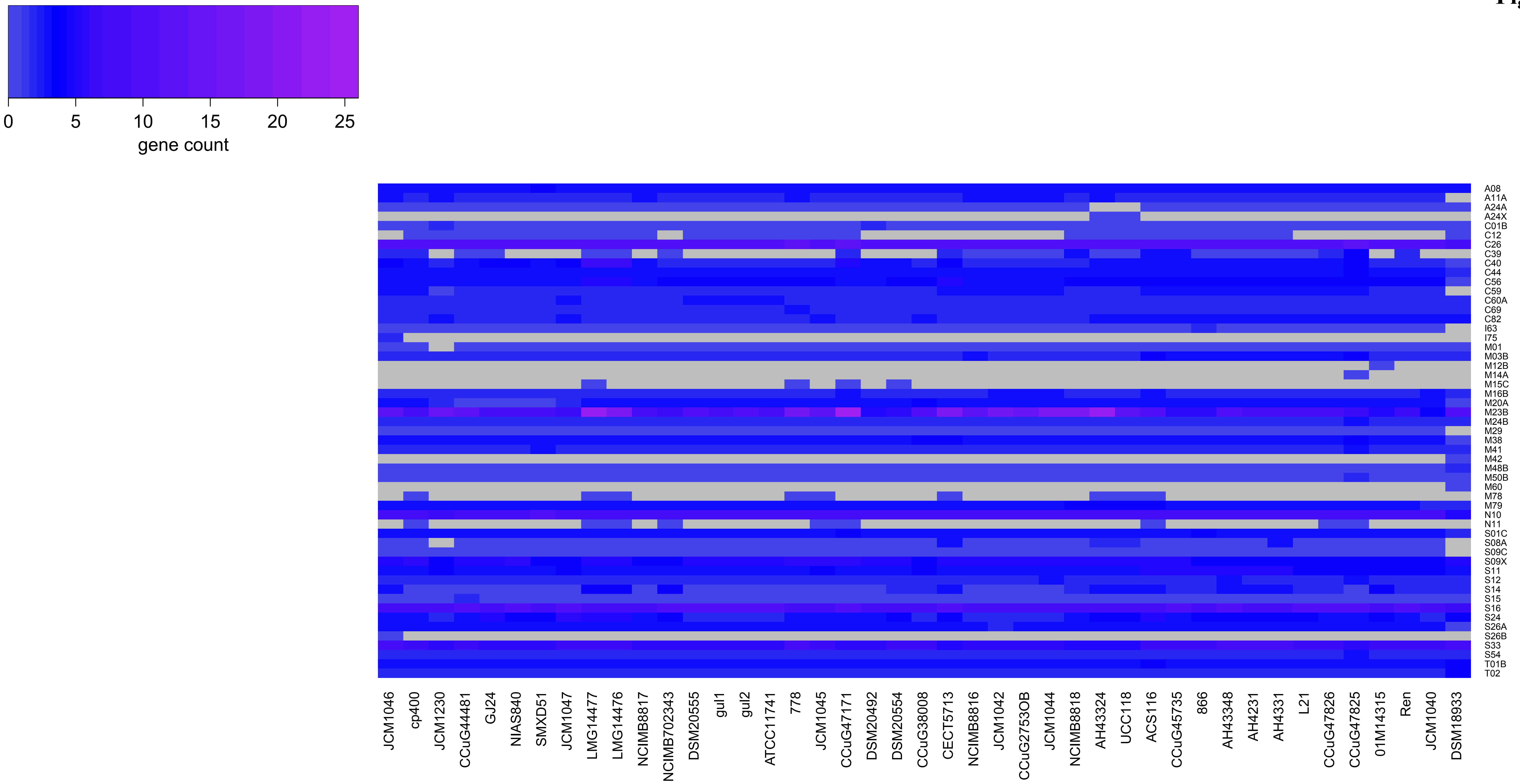

0
5
10
15
20
25
gene count
A08
A11A
A24A
A24X
C01B
C12
C26
C39
C40
C44
C56
C59
C60A
C69
C82
I63
I75
M01
M03B
M12B
M14A
M15C
M16B
M20A
M23B
M24B
M29
M38
M41
M42
M48B
M50B
M60
M78
M79
N10
N11
S01C
S08A
S09C
S09X
S11
S12
S14
S15
S16
S24
S26A
S26B
S33
S54
T01B
T02
JCM1046
cp400
JCM1230
CCuG44481
GJ24
NIAS840
SMXD51
JCM1047
LMG14477
LMG14476
NCIMB8817
NCIMB702343
DSM20555
gul1
gul2
ATCC11741
778
JCM1045
CCuG47171
DSM20492
DSM20554
CCuG38008
CECT5713
NCIMB8816
JCM1042
CCuG2753OB
JCM1044
NCIMB8818
AH43324
UCC118
ACS116
CCuG45735
866
AH43348
AH4231
AH4331
L21
CCuG47826
CCuG47825
01M14315
Ren
JCM1040
DSM18933

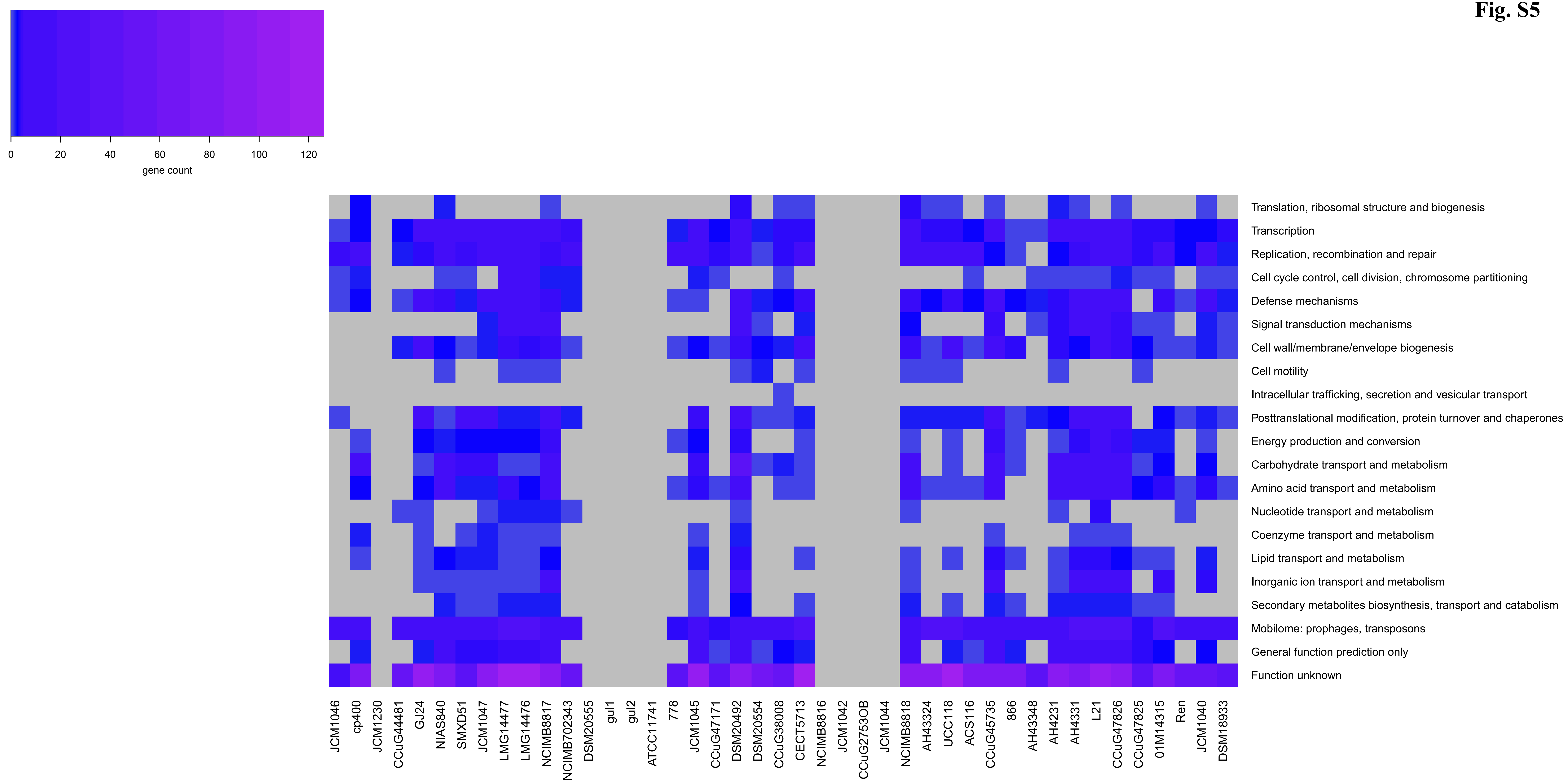
0
20
40
60
80
100
120
gene count
Translation, ribosomal structure and biogenesis
Transcription
Replication, recombination and repair
Cell cycle control, cell division, chromosome partitioning
Defense mechanisms
Signal transduction mechanisms
Cell wall/membrane/envelope biogenesis
Cell motility
Intracellular trafficking, secretion and vesicular transport
Posttranslational modification, protein turnover and chaperones
Energy production and conversion
Carbohydrate transport and metabolism
Amino acid transport and metabolism
Nucleotide transport and metabolism
Coenzyme transport and metabolism
Lipid transport and metabolism
Inorganic ion transport and metabolism
Secondary metabolites biosynthesis, transport and catabolism
Mobilome: prophages, transposons
General function prediction only
Function unknown
JCM1046
cp400
JCM1230
CCuG44481
GJ24
NIAS840
SMXD51
JCM1047
LMG14477
LMG14476
NCIMB8817
NCIMB702343
DSM20555
gul1
gul2
ATCC11741
778
JCM1045
CCuG47171
DSM20492
DSM20554
CCuG38008
CECT5713
NCIMB8816
JCM1042
CCuG27530B
JCM1044
NCIMB8818
AH43324
UCC118
ACS116
CCuG45735
866
AH43348
AH4231
AH4331
L21
CCuG47826
CCuG47825
01M14315
Ren
JCM1040
DSM18933

Fig. S6

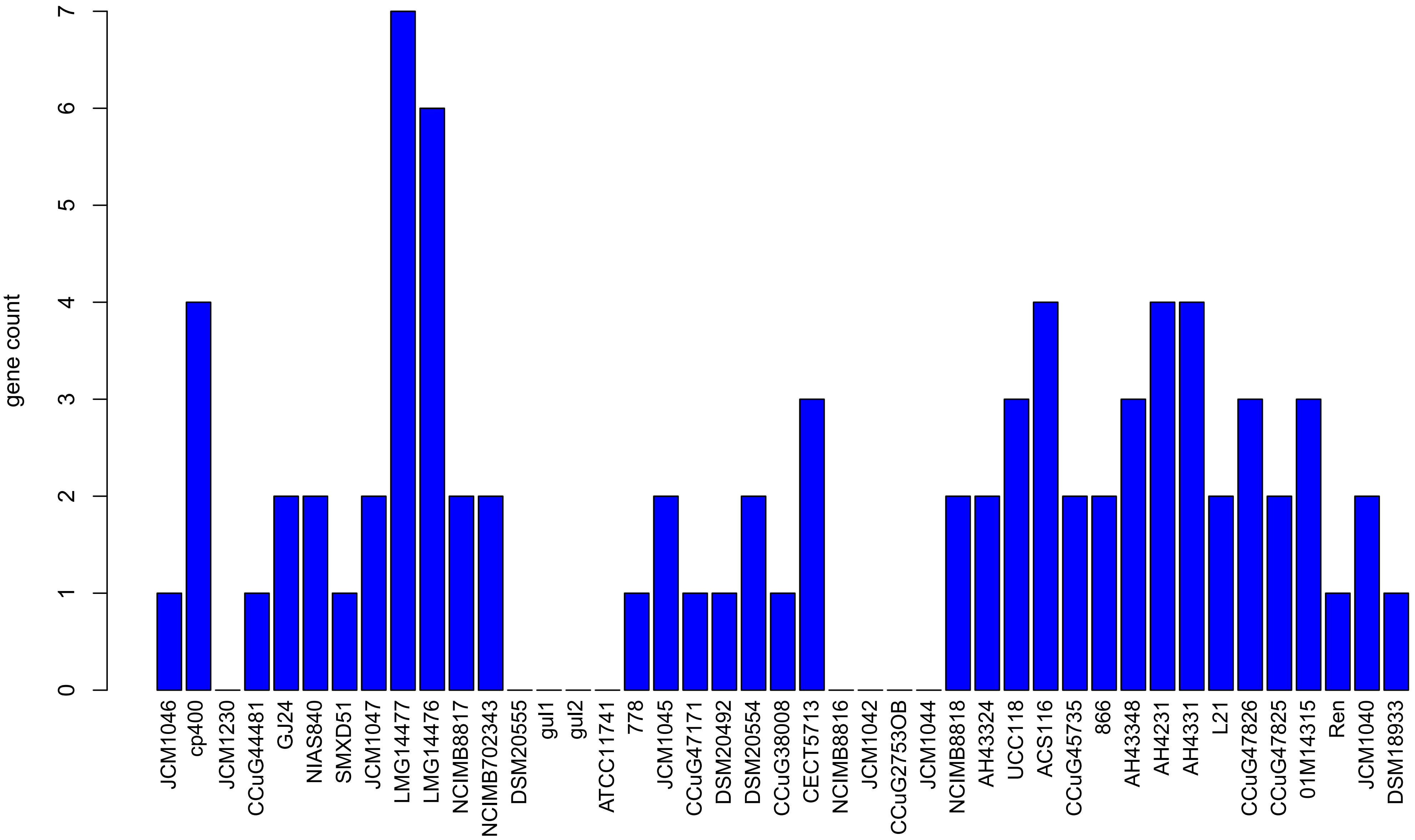

gene count
0
1
2
3
4
5
6
7
JCM1046
cp400
JCM1230
CCuG44481
GJ24
NIAS840
SMXD51
JCM1047
LMG14477
LMG14476
NCIMB8817
NCIMB702343
DSM20555
gul1
gul2
ATCC11741
778
JCM1045
CCuG47171
DSM20492
DSM20554
CCuG38008
CECT5713
NCIMB8816
JCM1042
CCuG2753OB
JCM1044
NCIMB8818
AH43324
UCC118
ACS116
CCuG45735
866
AH43348
AH4231
AH4331
L21
CCuG47826
CCuG47825
01M14315
Ren
JCM1040
DSM18933

Fig. S7

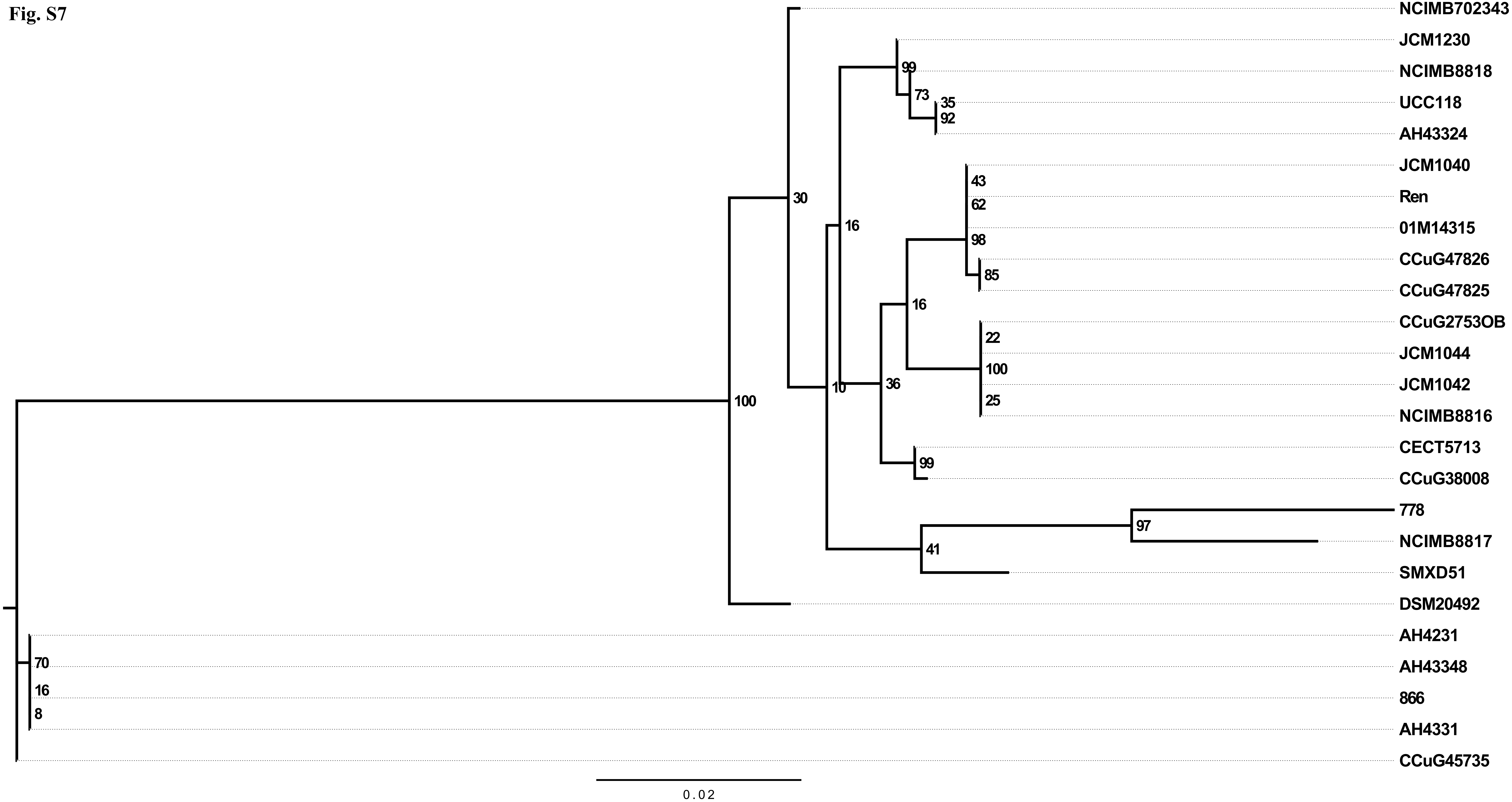
NCIMB702343
JCM1230
NCIMB8818
UCC118
AH43324
JCM1040
Ren
01M14315
CCuG47826
CCuG47825
CCuG2753OB
JCM1044
JCM1042
NCIMB8816
CECT5713
CCuG38008
778
NCIMB8817
SMXD51
DSM20492
AH4231
AH43348
866
AH4331
CCuG45735
100
30
16
10
99
73
35
92
43
62
98
85
16
22
100
25
36
99
97
41
70
16
8
0.02

Fig. S8

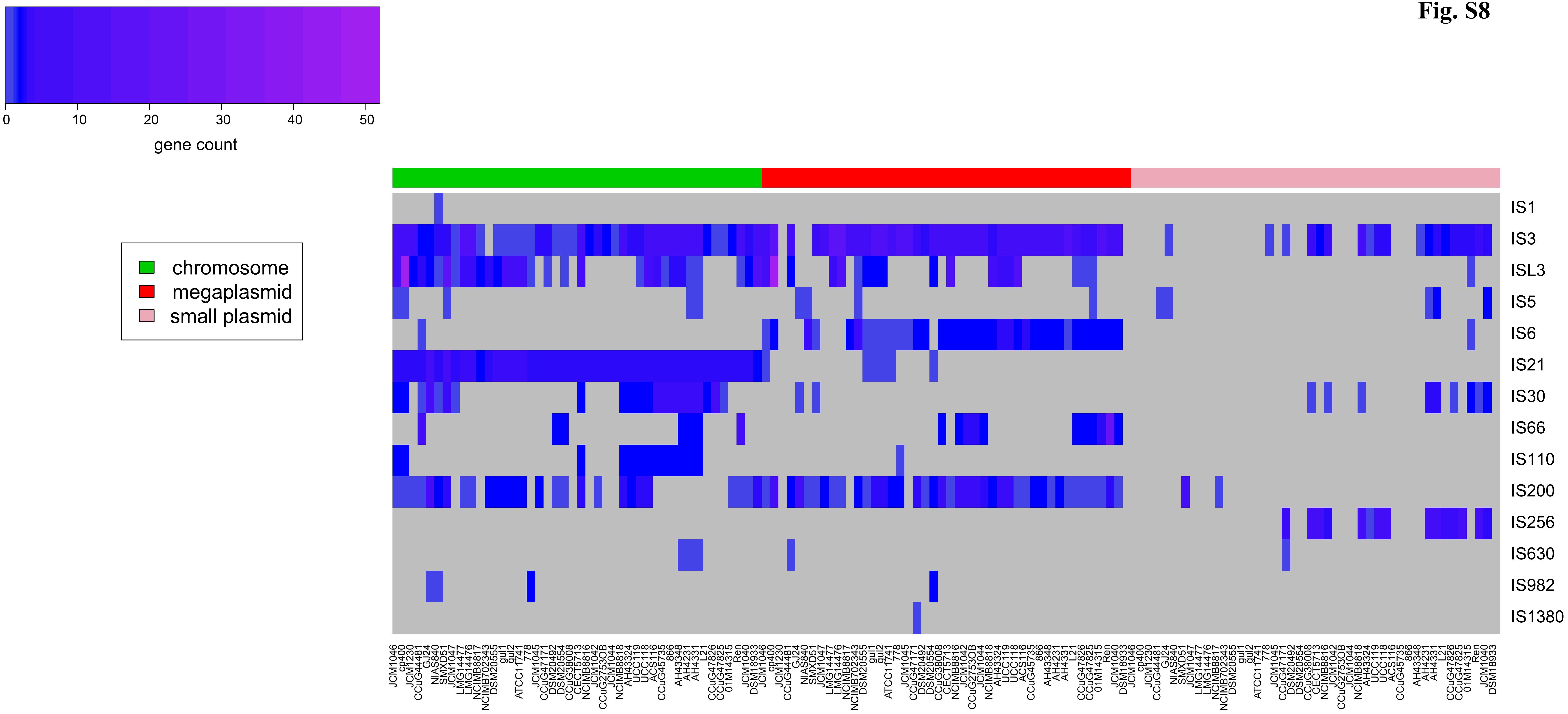
0
10
20
30
40
50
gene count
chromosome
megaplasmid
small plasmid
IS1
IS3
ISL3
IS5
IS6
IS21
IS30
IS66
IS110
IS200
IS256
IS630
IS982
IS1380

Fig. S9

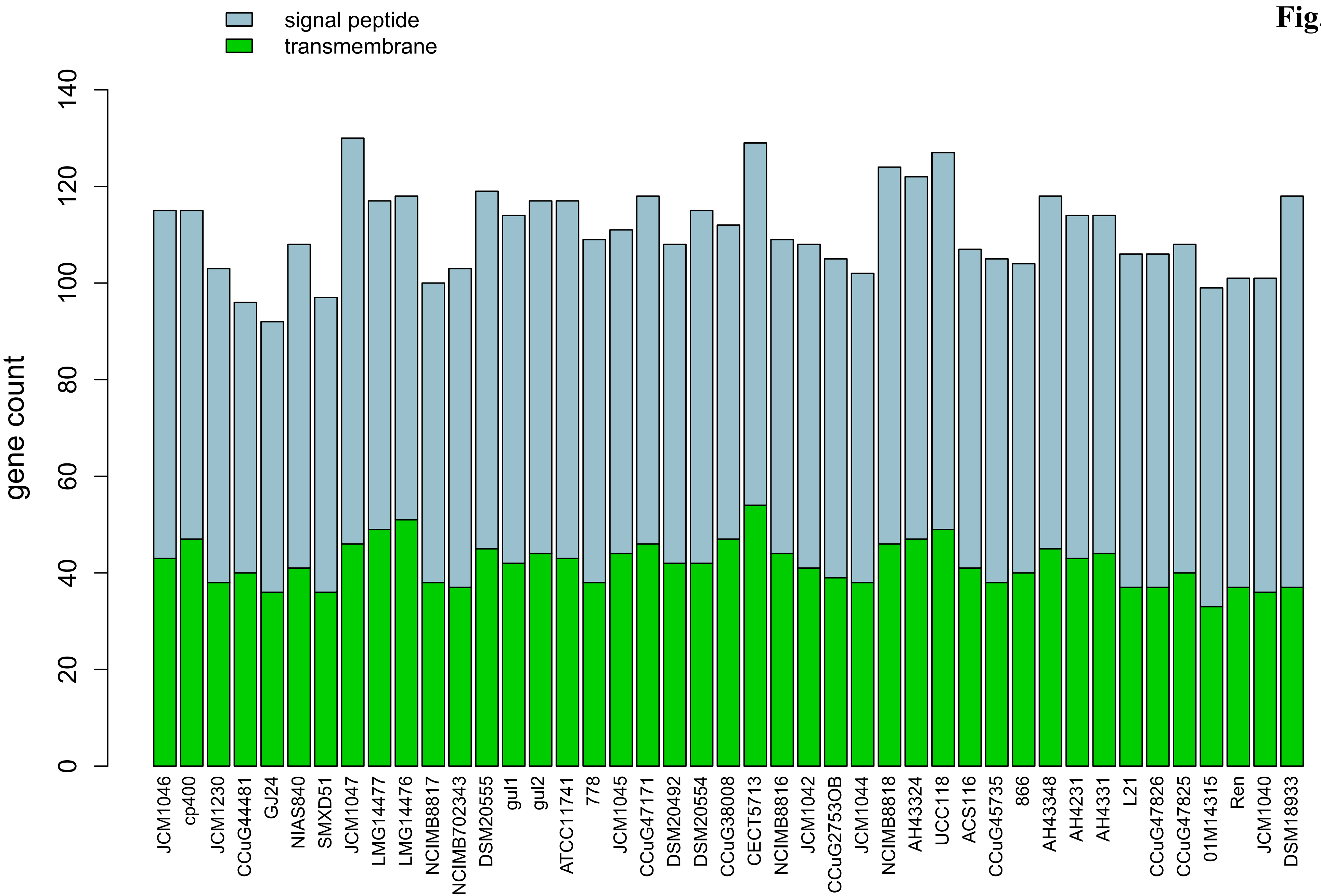
signal peptide
transmembrane
gene count
0
20
40
60
80
100
120
140
JCM1046
cp400
JCM1230
CCuG44481
GJ24
NIAS840
SMXD51
JCM1047
LMG14477
LMG14476
NCIMB8817
NCIMB702343
DSM20555
gul1
gul2
ATCC11741
778
JCM1045
CCuG47171
DSM20492
DSM20554
CCuG38008
CECT5713
NCIMB8816
JCM1042
CCuG2753OB
JCM1044
NCIMB8818
AH43324
UCC118
ACS116
CCuG45735
866
AH43348
AH4231
AH4331
L21
CCuG47826
CCuG47825
01M14315
Ren
JCM1040
DSM18933

Fig. S10

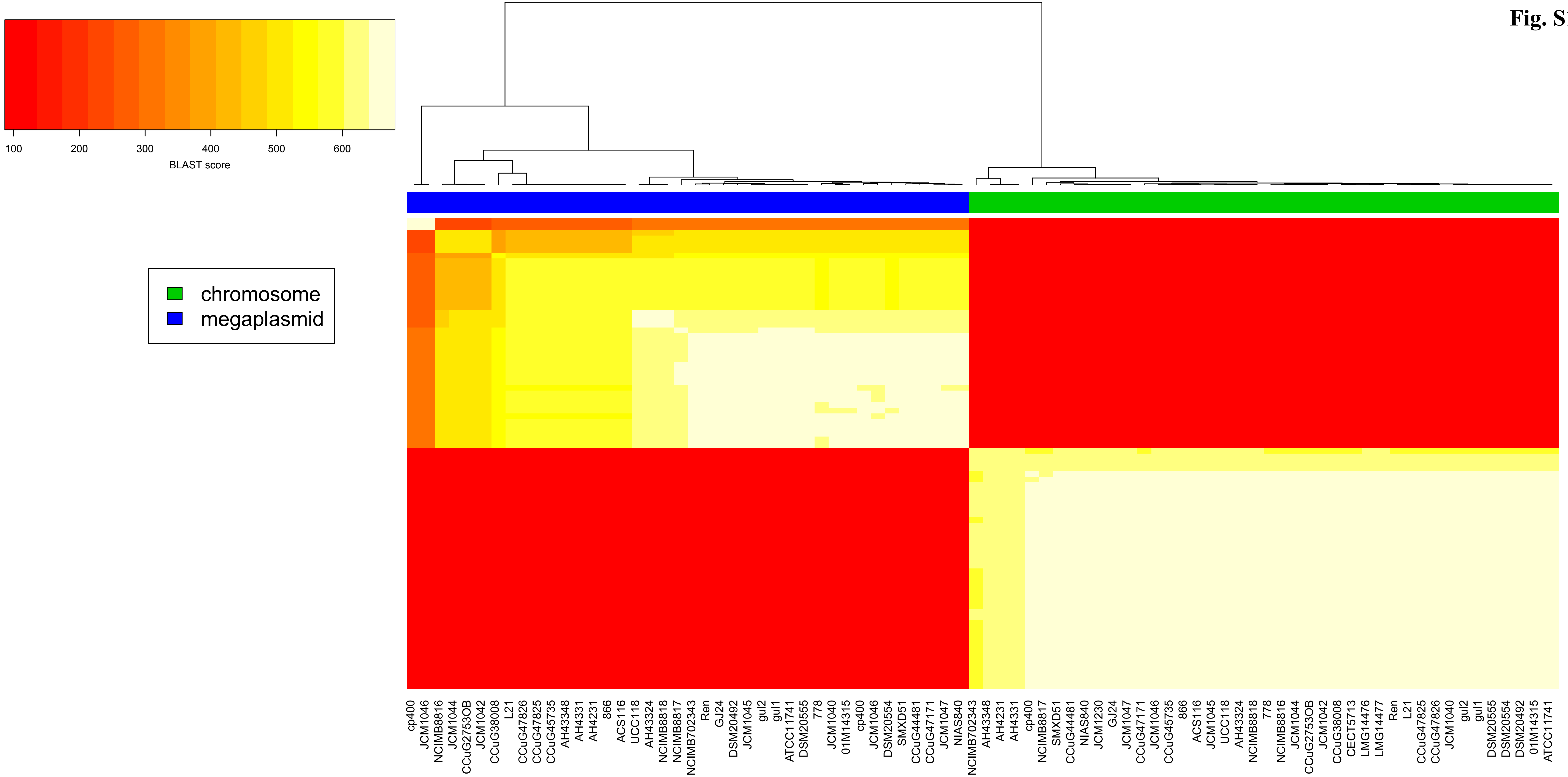
100
200
300
400
500
600
BLAST score
chromosome
megaplasmid
cp400
JCM1046
NCIMB8816
JCM1044
CCuG2753OB
JCM1042
CCuG38008
L21
CCuG47826
CCuG47825
CCuG45735
AH43348
AH4331
AH4231
866
ACS116
UCC118
AH43324
NCIMB8818
NCIMB8817
NCIMB702343
Ren
GJ24
DSM20492
JCM1045
gul2
gul1
ATCC11741
DSM20555
778
JCM1040
01M14315
cp400
JCM1046
DSM20554
SMXD51
CCuG44481
CCuG47171
JCM1047
NIAS840
NCIMB702343
AH43348
AH4231
AH4331
cp400
NCIMB8817
SMXD51
CCuG44481
NIAS840
JCM1230
GJ24
JCM1047
CCuG47171
JCM1046
CCuG45735
866
ACS116
JCM1045
UCC118
AH43324
NCIMB8818
778
NCIMB8816
JCM1044
CCuG2753OB
JCM1042
CCuG38008
CECT5713
LMG14476
LMG14477
Ren
L21
CCuG47825
CCuG47826
JCM1040
gul2
gul1
DSM20555
DSM20554
DSM20492
01M14315
ATCC11741

Fig. S11

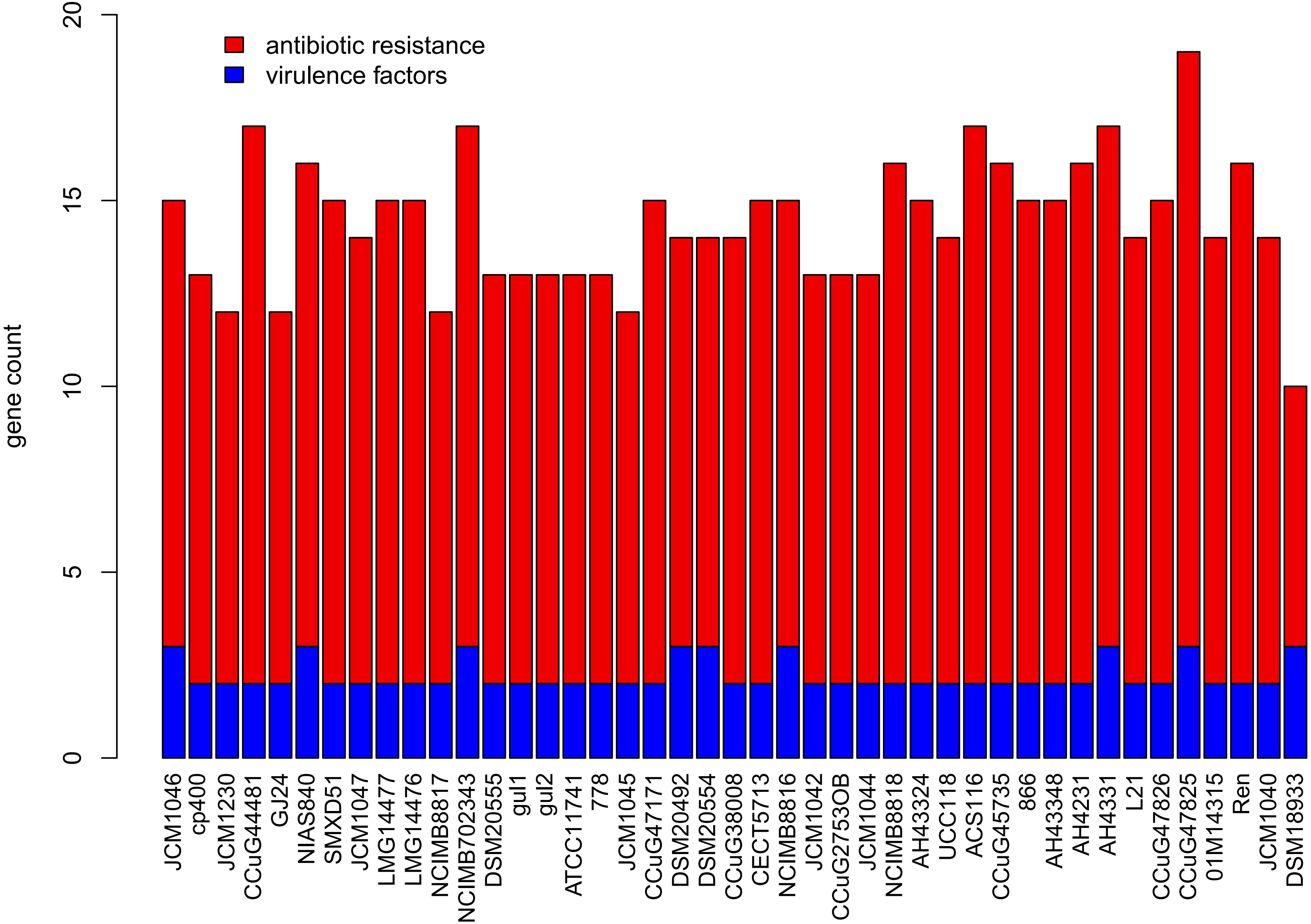
antibiotic resistance
virulence factors
gene count
0
5
10
15
20
JCM1046
cp400
JCM1230
CCuG44481
GJ24
NIAS840
SMXD51
JCM1047
LMG14477
LMG14476
NCIMB8817
NCIMB702343
DSM20555
gul1
gul2
ATCC11741
778
JCM1045
CCuG47171
DSM20492
DSM20554
CCuG38008
CECT5713
NCIMB8816
JCM1042
CCuG2753OB
JCM1044
NCIMB8818
AH43324
UCC118
ACS116
CCuG45735
866
AH43348
AH4231
AH4331
L21
CCuG47826
CCuG47825
01M14315
Ren
JCM1040
DSM18933

Table S1

	contigs	total size (bp)	max contig size (bp)	N50	GC %	gene count	circular megaplasmids	linear megaplasmids	small plasmids	isolation source	BioSample accession
ACS116	154	2044600	71067	28667	32.7	2210	1	0	0	human_vaginal_cavity	SAMN00017035
CCuG47825	120	1921003	93995	30969	32.8	2041	1	0	1	human_blood,55-year-old_female	SAMN06163258
LMG14477	243	2080357	139646	31335	32.8	2238	1	0	1	parakeet_with_sepsis	SAMN06163272
LMG14476	238	2087383	139606	38865	32.8	2243	1	0	1	cat_with_myocarditis	SAMN06163271
DSM18933	216	1699788	420945	47652	34	1844	0	0	0	faeces_of_thoroughbred_horse	SAMD00008721
cp400	89	2148103	152599	48732	32.8	2286	1	0	0	pre-weaned_piglet_faeces	SAMEA3138854
AH4331	177	2090404	196933	51266	32.9	2254	1	0	2	human_ileo-cecal_region	SAMN06163250
AH4231	186	2095546	196492	51450	32.9	2260	1	0	2	human_ileo-cecal_region	SAMN06163249
AH43324	114	2176341	196739	53856	32.8	2319	1	0	1	human_ileo-cecal_region	SAMN06163251
CCuG45735	77	1949679	199587	61326	32.7	1982	1	0	1	human_blood	SAMN06163256
CCuG44481	119	1946114	255195	63223	32.7	2013	1	0	1	bird	SAMN06163255
866	82	1973474	239143	67307	32.6	2019	1	0	0	clinical_isolate_ICU	SAMN03198074
AH43348	109	2026883	186083	73962	32.6	2177	1	1	1	human_ileo-cecal_region	SAMN06163252
01M14315	87	1933921	212516	78580	33	1965	1	0	1	human_gallbladder_pus	SAMN06163248
DSM20555	63	1982794	244105	80533	32.4	2000	1	0	0	human_saliva	SAMN02369414
L21	74	1956328	215606	95624	32.7	2015	1	0	1	human_faeces	SAMN06163270
NCIMB8816	77	1859216	465152	106164	32.8	1886	1	0	2	human_saliva	SAMN06163274
JCM1044	59	1800424	465193	115042	32.6	1799	1	0	0	human_intestine	SAMN06163266
JCM1042	60	1802147	465022	115144	32.6	1800	1	0	0	human_intestine	SAMN06163265
CCuG2753OB	48	1801346	465248	115144	32.6	1799	1	0	0	human_abdomen_abcess	SAMN06163253
NCIMB702343	83	1922903	646361	119088	32.8	1953	1	0	0	unknown	SAMN06163273
CCuG38008	91	1936298	463867	122107	32.7	1972	1	0	2	human_gall,73-year-old_male	SAMN06163254
gul-2	81	2002733	521962	126351	32.5	2027	1	0	0	root_canal	SAMN06163263
ATCC11741	54	1995868	274210	126392	32.5	2011	1	0	0	human_HMP_ref	SAMN00001483
JCM1040	66	1922028	421976	133271	32.8	1951	1	0	1	human_intestine	SAMN06163264
CCuG47171	140	2040146	353585	136925	32.9	2152	1	0	2	human_tooth_plaque	SAMN06163257
CCuG47826	71	1980366	279863	142710	32.8	2044	1	0	1	human_blood,55-year-old_female	SAMN06163259
JCM1230	82	1723361	400300	151438	32.6	1719	0	0	0	chicken_intestine	SAMN06163269
JCM1045	69	1928686	331541	153948	32.7	1964	1	0	0	human_intestine	SAMN06163267
gul-1	75	2001390	521857	162200	32.5	2026	1	0	0	root_canal	SAMN06163262
778	44	1942335	332399	164413	32.7	1947	1	0	1	clinical_isolate_ICU	SAMN03197988
DSM20554	74	1975060	334317	170258	32.6	2026	1	0	1	human_saliva	SAMN06163261
JCM1047	147	2222264	475807	178703	32.4	2345	1	1	1	swine_intestine	SAMN06163268
NCIMB8818	79	2013336	250622	180395	32.9	2103	1	0	1	St_Ivel_cheese	SAMN06163276
DSM20492	32	1889334	481851	240870	32.6	1892	1	0	0	human_saliva	SAMN06163260
NCIMB8817	56	1831814	777665	294813	32.6	1852	1	0	1	turkey_faeces	SAMN06163275
GJ24	11	1995968	754247	502388	33	2028	1	0	1	human_intestine	SAMN02470918
SMXD51	10	1967688	1019433	1019433	32.9	1992	1	0	2	chicken_cecum	SAMN02470767
NIAS840	4	2046557	1705688	1705688	33	2032	2	0	1	chicken_faeces	SAMN02470897
Ren	3	1978364	1751565	1751565	33	2019	1	0	1	human_centanarian_faeces	SAMN02584770
UCC118	4	2133977	1827111	1827111	33	2264	1	0	2	human_ileo-cecal_region	SAMN02604111
CECT5713	4	2136138	1828169	1828169	33.1	2345	1	0	2	human_breast_milk/infant_faeces	SAMN02604101
JCM1046	5	2320461	1836297	1836297	32.9	2296	2	1	1	swine_intestine	SAMN02711722

Table S2

strain	gene	contig	replicon	% identity	alignment length	e-value	BLAST score	gene length
778	repA_LSL1739	NODE_46	MP	99.21	254	8.00E-165	507	254
778	repE_LSL1740	NODE_46	MP	99.09	330	0	666	330
778	parA_LSL1741	NODE_46	MP	99.63	270	2.00E-179	550	270
866	repA_LSL1739	NODE_77	MP	99.21	254	8.00E-165	507	254
866	repE_LSL1740	NODE_77	MP	96.06	330	0	644	330
866	parA_LSL1741	NODE_77	MP	99.63	270	2.00E-179	550	270
01M14315	repA_LSL1739	contig_34	MP	99.21	254	7.00E-165	507	254
01M14315	repE_LSL1740	contig_34	MP	99.09	330	0	665	330
01M14315	parA_LSL1741	contig_34	MP	99.63	270	1.00E-179	550	270
ACS116	repA_LSL1739	contig_13	MP	98.82	254	1.00E-166	505	254
ACS116	repE_LSL1740	contig_13	MP	99.09	330	0	667	330
ACS116	parA_LSL1741	contig_139	MP	99.16	239	1.00E-169	484	270
AH4231	repA_LSL1739	contig_95	MP	99.21	254	8.00E-165	507	254
AH4231	repE_LSL1740	contig_95	MP	98.79	330	0	664	330
AH4231	parA_LSL1741	contig_95	MP	99.63	270	2.00E-179	550	270
AH4331	repA_LSL1739	contig_101	MP	99.21	254	8.00E-165	507	254
AH4331	repE_LSL1740	contig_101	MP	98.79	330	0	664	330
AH4331	parA_LSL1741	contig_101	MP	99.63	270	2.00E-179	550	270
AH43324	repA_LSL1739	contig_44	MP	99.61	254	5.00E-166	510	254
AH43324	repE_LSL1740	contig_44	MP	95.45	330	0	644	330
AH43324	parA_LSL1741	contig_44	MP	100	270	3.00E-180	551	270
AH43348	repA_LSL1739	contig_70	MP	99.21	254	9.00E-165	507	254
AH43348	repE_LSL1740	contig_70	MP	98.79	330	0	664	330
AH43348	parA_LSL1741	contig_70	MP	99.63	270	2.00E-179	550	270
AH43348	parA_LSL1741	contig_69	MPL	93.7	270	3.00E-169	520	270
ATCC11741	repA_LSL1739	contig_37	MP	99.21	254	9.00E-165	506	254
ATCC11741	repE_LSL1740	contig_37	MP	96.06	330	0	644	330
ATCC11741	parA_LSL1741	contig_37	MP	99.63	270	1.00E-179	550	270
CCuG2753OB	repA_LSL1739	contig_21	MP	99.21	254	7.00E-165	507	254
CCuG2753OB	repE_LSL1740	contig_21	MP	98.79	330	0	663	330
CCuG2753OB	parA_LSL1741	contig_21	MP	98.89	270	3.00E-178	546	270
CCuG38008	repA_LSL1739	contig_40	MP	99.21	254	5.00E-165	507	254
CCuG38008	repE_LSL1740	contig_40	MP	97.27	330	0	659	330
CCuG38008	parA_LSL1741	contig_40	MP	99.63	270	1.00E-179	550	270
CCuG44481	repA_LSL1739	contig_46	MP	99.21	254	7.00E-165	507	254
CCuG44481	repE_LSL1740	contig_46	MP	96.36	330	0	646	330
CCuG44481	parA_LSL1741	contig_39	MP	99.63	270	0	550	270
CCuG45735	repA_LSL1739	contig_51	MP	99.21	254	8.00E-165	507	254
CCuG45735	repE_LSL1740	contig_51	MP	99.39	330	0	668	330
CCuG45735	parA_LSL1741	contig_51	MP	99.63	270	2.00E-179	550	270
CCuG47171	repA_LSL1739	contig_58	MP	98.43	254	3.00E-163	503	254
CCuG47171	repE_LSL1740	contig_58	MP	96.97	330	0	650	330
CCuG47171	parA_LSL1741	contig_58	MP	100	270	4.00E-180	551	270
CCuG47825	repA_LSL1739	contig_92	MP	99.21	254	7.00E-165	507	254
CCuG47825	repE_LSL1740	contig_92	MP	99.39	330	0	668	330
CCuG47825	parA_LSL1741	contig_91	MP	99.63	270	0	550	270
CCuG47826	repA_LSL1739	contig_36	MP	99.21	254	8.00E-165	507	254
CCuG47826	repE_LSL1740	contig_36	MP	99.39	330	0	668	330
CCuG47826	parA_LSL1741	contig_36	MP	99.63	270	2.00E-179	550	270
CECT5713	repA_LSL1739	contig_4	MP	99.21	254	9.00E-165	507	254
CECT5713	repE_LSL1740	contig_4	MP	97.27	330	0	659	330
CECT5713	parA_LSL1741	contig_4	MP	99.63	270	2.00E-179	550	270
cp400	repA_LSL1739	contig_16	MP	99.21	254	7.00E-165	507	254
cp400	repE_LSL1740	contig_16	MP	96.36	330	0	646	330
cp400	parA_LSL1741	contig_16	MP	99.63	270	2.00E-179	550	270
DSM18933	repA_LSL1739	NODE_63	C	78.74	254	3.00E-132	413	254
DSM18933	parA_LSL1741	NODE_63	C	86.62	269	8.00E-157	484	270
DSM20492	repA_LSL1739	contig_14	MP	98.82	254	3.00E-164	505	254
DSM20492	repE_LSL1740	contig_14	MP	95.76	330	0	644	330
DSM20492	parA_LSL1741	contig_14	MP	99.63	270	2.00E-179	550	270

DSM20554	repA_LSL1739	contig_26	MP	99.21	254	8.00E-165	507	254
DSM20554	repE_LSL1740	contig_26	MP	92.73	330	0	626	330
DSM20554	parA_LSL1741	contig_26	MP	99.26	270	1.00E-178	547	270
DSM20555	repA_LSL1739	Scaffold29	MP	99.21	254	6.00E-165	506	254
DSM20555	repE_LSL1740	Scaffold29	MP	96.06	330	0	644	330
DSM20555	parA_LSL1741	Scaffold33	MP	99.63	270	3.00E-180	550	270
GJ24	repA_LSL1739	contig_9	MP	99.61	254	2.00E-165	509	254
GJ24	repE_LSL1740	contig_9	MP	95.15	330	0	641	330
GJ24	parA_LSL1741	contig_9	MP	99.63	270	2.00E-179	550	270
gul1	repA_LSL1739	contig_17	MP	99.21	254	9.00E-165	506	254
gul1	repE_LSL1740	contig_17	MP	96.06	330	0	644	330
gul1	parA_LSL1741	contig_17	MP	99.63	270	1.00E-179	550	270
gul2	repA_LSL1739	contig_18	MP	99.21	254	9.00E-165	506	254
gul2	repE_LSL1740	contig_18	MP	96.06	330	0	644	330
gul2	parA_LSL1741	contig_18	MP	99.63	270	1.00E-179	550	270
JCM1040	repA_LSL1739	contig_30	MP	99.21	254	7.00E-165	507	254
JCM1040	repE_LSL1740	contig_30	MP	99.7	330	0	671	330
JCM1040	parA_LSL1741	contig_30	MP	99.26	270	7.00E-179	548	270
JCM1042	repA_LSL1739	contig_27	MP	99.21	254	7.00E-165	507	254
JCM1042	repE_LSL1740	contig_27	MP	98.79	330	0	663	330
JCM1042	parA_LSL1741	contig_27	MP	98.89	270	3.00E-178	546	270
JCM1044	repA_LSL1739	contig_23	MP	99.21	254	7.00E-165	507	254
JCM1044	repE_LSL1740	contig_23	MP	98.79	330	0	663	330
JCM1044	parA_LSL1741	contig_23	MP	98.89	270	3.00E-178	546	270
JCM1045	repA_LSL1739	contig_30	MP	99.21	254	8.00E-165	507	254
JCM1045	repE_LSL1740	contig_30	MP	99.39	330	0	668	330
JCM1045	parA_LSL1741	contig_30	MP	99.63	270	2.00E-179	550	270
JCM1046	repA_LSL1739	contig_65	MP	99.21	254	8.00E-166	507	254
JCM1046	repE_LSL1740	contig_65	MP	96.67	330	0	648	330
JCM1046	parA_LSL1741	contig_65	MP	99.63	270	0	550	270
JCM1047	repA_LSL1739	contig_34	MP	98.43	254	3.00E-163	503	254
JCM1047	repE_LSL1740	contig_34	MP	95.45	330	0	642	330
JCM1047	parA_LSL1741	contig_34	MP	99.63	270	2.00E-179	550	270
L21	repA_LSL1739	contig_44	MP	99.21	254	8.00E-165	507	254
L21	repE_LSL1740	contig_44	MP	99.39	330	0	668	330
L21	parA_LSL1741	contig_44	MP	99.63	270	2.00E-179	550	270
LMG14476	repA_LSL1739	contig_42	MP	98.82	254	3.00E-164	505	254
LMG14476	repE_LSL1740	contig_42	MP	95.15	330	0	640	330
LMG14476	parA_LSL1741	contig_42	MP	99.63	270	2.00E-179	550	270
LMG14477	repA_LSL1739	contig_41	MP	98.82	254	3.00E-164	505	254
LMG14477	repE_LSL1740	contig_41	MP	95.15	330	0	640	330
LMG14477	parA_LSL1741	contig_41	MP	99.63	270	2.00E-179	550	270
NCIMB702343	repA_LSL1739	NODE_62	MP	99.61	254	2.00E-165	509	254
NCIMB702343	repE_LSL1740	NODE_62	MP	95.45	330	0	644	330
NCIMB702343	parA_LSL1741	NODE_62	MP	99.63	270	2.00E-179	550	270
NCIMB8816	repA_LSL1739	contig_35	MP	99.21	254	7.00E-165	507	254
NCIMB8816	repE_LSL1740	contig_35	MP	98.79	330	0	663	330
NCIMB8816	parA_LSL1741	contig_35	MP	98.89	270	3.00E-178	546	270
NCIMB8817	repA_LSL1739	contig_19	MP	99.21	254	7.00E-165	507	254
NCIMB8817	repE_LSL1740	contig_19	MP	96.36	330	0	645	330
NCIMB8817	parA_LSL1741	contig_19	MP	99.63	270	2.00E-179	550	270
NCIMB8818	repA_LSL1739	contig_33	MP	99.21	254	8.00E-165	507	254
NCIMB8818	repE_LSL1740	contig_33	MP	99.39	330	0	668	330
NCIMB8818	parA_LSL1741	contig_33	MP	99.63	270	2.00E-179	550	270
NIAS840	repA_LSL1739	contig_3	MP	98.43	254	3.00E-163	503	254
NIAS840	repE_LSL1740	contig_3	MP	95.45	330	0	642	330
NIAS840	parA_LSL1741	contig_3	MP	100	270	4.00E-180	551	270
Ren	repA_LSL1739	plasmid_1	MP	99.21	254	8.00E-165	507	254
Ren	repE_LSL1740	plasmid_1	MP	99.09	330	0	665	330
Ren	parA_LSL1741	plasmid_1	MP	99.63	270	2.00E-179	550	270
SMXD51	repA_LSL1739	contig_7	MP	99.44	179	8.00E-140	357	254

SMXD51	repE_LSL1740	contig_7	MP	96.36	330	0	646	330
SMXD51	parA_LSL1741	contig_7	MP	99.63	270	2.00E-179	550	270
UCC118	repA_LSL1739	contig_4	MP	100	254	2.00E-166	511	254
UCC118	repE_LSL1740	contig_4	MP	95.45	330	0	644	330
UCC118	parA_LSL1741	contig_4	MP	100	270	5.00E-180	551	270

Table S3

strain	AOI	replicon	bacteriocin	class
778	AOI 1	chromosome	enterolysin A	III
778	AOI 1	chromosome	enterolysin A	III
866	AOI 1	chromosome	enterolysin A	III
866	AOI 1	chromosome	enterolysin A	III
866	AOI 1	megaplasmid	salivaricin P	II
01M14315	AOI 1	chromosome	enterolysin A	III
01M14315	AOI 1	chromosome	enterolysin A	III
ACS116	AOI 1	chromosome	enterolysin A	III
ACS116	AOI 1	chromosome	enterolysin A	III
ACS116	AOI 1	chromosome	enterolysin A	III
ACS116	AOI 1	chromosome	enterolysin A	III
ACS116	AOI 1	megaplasmid	salivaricin P	II
AH4231	AOI 1	chromosome	enterolysin A	III
AH4231	AOI 1	chromosome	enterolysin A	III
AH4231	AOI 1	chromosome	enterolysin A	III
AH4231	AOI 1	megaplasmid	salivaricin P	II
AH4331	AOI 1	chromosome	enterolysin A	III
AH4331	AOI 1	chromosome	enterolysin A	III
AH4331	AOI 1	chromosome	enterolysin A	III
AH4331	AOI 1	megaplasmid	salivaricin P	II
AH43324	AOI 1	chromosome	enterolysin A	III
AH43324	AOI 1	chromosome	enterolysin A	III
AH43324	AOI 1	chromosome	enterolysin A	III
AH43324	AOI 1	megaplasmid	salivaricin P	II
AH43348	AOI 1	chromosome	enterolysin A	III
AH43348	AOI 1	chromosome	enterolysin A	III
AH43348	AOI 1	chromosome	enterolysin A	III
AH43348	AOI 1	megaplasmid	salivaricin P	II
ATCC11741	AOI 1	chromosome	enterolysin A	III
CCUG2753OB	AOI 1	chromosome	enterolysin A	III
CCUG2753OB	AOI 1	megaplasmid	LS2	II
CCUG38008	AOI 1	chromosome	enterolysin A	III
CCUG38008	AOI 1	chromosome	enterolysin A	III
CCUG38008	AOI 1	megaplasmid	salivaricin P	II
CCUG44481	AOI 1	chromosome	enterolysin A	III
CCUG44481	AOI 1	megaplasmid	plantaricin S	II
CCUG44481	AOI 2	megaplasmid	plantaricin NC8	II
CCUG44481	AOI 2	megaplasmid	lactacin F	II
CCUG44481	AOI 2	megaplasmid	acidocin LF221B	II
CCUG44481	AOI 2	megaplasmid	salivaricin P	II
CCUG45735	AOI 1	chromosome	enterolysin A	III
CCUG45735	AOI 1	chromosome	enterolysin A	III
CCUG45735	AOI 1	megaplasmid	salivaricin P	II
CCUG47171	AOI 1	chromosome	enterolysin A	III
CCUG47171	AOI 1	chromosome	enterolysin A	III
CCUG47171	AOI 1	megaplasmid	plantaricin NC8	II
CCUG47171	AOI 1	megaplasmid	lactacin F	II
CCUG47171	AOI 1	megaplasmid	acidocin LF221B	II
CCUG47171	AOI 1	megaplasmid	salivaricin P	II

CCUG47825	AOI 1	chromosome	enterolysin A	III
CCUG47825	AOI 1	chromosome	enterolysin A	III
CCUG47826	AOI 1	chromosome	enterolysin A	III
CCUG47826	AOI 1	chromosome	enterolysin A	III
CCUG47826	AOI 1	chromosome	enterolysin A	III
CCUG47826	AOI 1	megaplasmid	salivaricin P	II
CECT5713	AOI 2	chromosome	enterolysin A	III
CECT5713	AOI 3	chromosome	enterolysin A	III
CECT5713	AOI 4	chromosome	enterolysin A	III
CECT5713	AOI 1	megaplasmid	salivaricin P	II
cp400	AOI 1	chromosome	enterolysin A	III
cp400	AOI 1	chromosome	enterolysin A	III
cp400	AOI 1	megaplasmid	salivaricin P	II
DSM18933	AOI 1	chromosome	enterolysin A	III
DSM18933	AOI 1	chromosome	enterolysin A	III
DSM20492	AOI 1	chromosome	enterolysin A	III
DSM20554	AOI 1	chromosome	enterolysin A	III
DSM20554	AOI 1	chromosome	enterolysin A	III
DSM20554	AOI 1	small plasmid	MR10B	II
DSM20555	AOI 1	chromosome	enterolysin A	III
GJ24	AOI 1	chromosome	enterolysin A	III
GJ24	AOI 1	megaplasmid	plantaricin S	II
GJ24	AOI 2	megaplasmid	plantaricin NC8	II
GJ24	AOI 2	megaplasmid	salivaricin P	II
gul1	AOI 1	chromosome	enterolysin A	III
gul2	AOI 1	chromosome	enterolysin A	III
JCM1040	AOI 1	chromosome	enterolysin A	III
JCM1040	AOI 1	chromosome	enterolysin A	III
JCM1042	AOI 1	chromosome	enterolysin A	III
JCM1042	AOI 1	megaplasmid	LS2	II
JCM1044	AOI 1	chromosome	enterolysin A	III
JCM1044	AOI 1	megaplasmid	LS2	II
JCM1045	AOI 1	chromosome	enterolysin A	III
JCM1045	AOI 1	megaplasmid	enterolysin A	III
JCM1046	AOI 1	chromosome	enterolysin A	III
JCM1046	AOI 1	megaplasmid	salivaricin P	II
JCM1046	AOI 1	linear megaplasmid	enterolysin A	III
JCM1046	AOI 1	small plasmid	MR10B	II
JCM1047	AOI 1	chromosome	enterolysin A	III
JCM1047	AOI 1	small plasmid	MR10B	II
JCM1047	AOI 1	megaplasmid	salivaricin P	II
JCM1047	AOI 1	linear megaplasmid	enterolysin A	III
JCM1230	AOI 1	chromosome	enterolysin A	III
L21	AOI 1	chromosome	enterolysin A	III
L21	AOI 1	chromosome	enterolysin A	III
L21	AOI 1	megaplasmid	salivaricin P	II
LMG14476	AOI 1	chromosome	enterolysin A	III
LMG14476	AOI 1	chromosome	enterolysin A	III
LMG14476	AOI 1	megaplasmid	salivaricin P	II
LMG14477	AOI 1	chromosome	enterolysin A	III

LMG14477	AOI 1	chromosome	enterolysin A	III
LMG14477	AOI 1	megaplasmid	salivaricin P	II
NCIMB702343	AOI 1	chromosome	enterolysin A	III
NCIMB702343	AOI 1	chromosome	enterolysin A	III
NCIMB702343	AOI 1	megaplasmid	salivaricin P	II
NCIMB8816	AOI 1	megaplasmid	LS2	II
NCIMB8816	AOI 1	chromosome	enterolysin A	III
NCIMB8817	AOI 1	chromosome	enterolysin A	III
NCIMB8818	AOI 1	chromosome	enterolysin A	III
NCIMB8818	AOI 1	chromosome	enterolysin A	III
NCIMB8818	AOI 1	megaplasmid	salivaricin P	II
NIAS840	AOI 1	chromosome	enterolysin A	III
Ren	AOI 1	chromosome	enterolysin A	III
Ren	AOI 2	chromosome	enterolysin A	III
SMXD51	AOI 1	chromosome	enterolysin A	III
SMXD51	AOI 1	megaplasmid	LS2	II
UCC118	AOI 1	chromosome	enterolysin A	III
UCC118	AOI 2	chromosome	enterolysin A	III
UCC118	AOI 3	chromosome	enterolysin A	III
UCC118	AOI 1	megaplasmid	salivaricin P	II

Table S4

strain	classification	taxon nucleotide %	taxon nucleotides	contig count
01M14315	Lactobacillus_salivarius	97.9012	1755411	67
01M14315	unclassified	1.96655	35261	6
01M14315	Lactobacillus_fermentum	0.124593	2234	1
01M14315	Lactobacillus_casei_group	0.00764064	137	1
778	Lactobacillus_salivarius	99.3133	1921066	36
778	unclassified	0.686742	13284	1
866	Lactobacillus_salivarius	100	1963667	75
ACS116	Lactobacillus_salivarius	100	1956854	132
AH4231	Lactobacillus_salivarius	97.1495	1950134	121
AH4231	unclassified	2.45587	49298	32
AH4231	Lactobacillus_casei_group	0.212867	4273	9
AH4231	Lactobacillus_fermentum	0.181732	3648	2
AH4331	Lactobacillus_salivarius	97.1714	1947771	115
AH4331	unclassified	2.47302	49571	33
AH4331	Lactobacillus_casei_group	0.181594	3640	8
AH4331	Lactobacillus_fermentum	0.174011	3488	1
AH43324	Lactobacillus_salivarius	98.4574	1942606	87
AH43324	unclassified	1.54259	30436	1
AH43348	Lactobacillus_salivarius	100	2028360	109
ATCC11741	Lactobacillus_salivarius	99.9018	1897727	40
ATCC11741	unclassified	0.0484841	921	4
ATCC11741	Corynebacterium_aurimucosum	0.0243736	463	2
ATCC11741	Erysipelothrix_rhusiopathiae	0.0126869	241	1
ATCC11741	Rhodococcus_equi	0.0126869	241	1
CCuG2753OB	Lactobacillus_salivarius	100	1790291	42
CCuG38008	Lactobacillus_salivarius	98.1458	1895829	67
CCuG38008	unclassified	1.82684	35288	10
CCuG38008	Lactobacillus_casei_group	0.0273342	528	2
CCuG44481	Lactobacillus_salivarius	96.0994	1812482	54
CCuG44481	unclassified	2.94823	55605	5
CCuG44481	Lactobacillus_reuteri	0.738793	13934	1
CCuG44481	Clostridium_sp._SY8519	0.213621	4029	1
CCuG45735	Lactobacillus_salivarius	99.9105	1947895	69
CCuG45735	Lactobacillus_helveticus	0.0802713	1565	1
CCuG45735	unclassified	0.00918119	179	1
CCuG47171	Lactobacillus_salivarius	97.6392	1974695	82
CCuG47171	unclassified	1.87664	37954	22
CCuG47171	Lactobacillus_casei_group	0.32352	6543	7
CCuG47171	Lactobacillus_plantarum	0.160598	3248	1
CCuG47825	Lactobacillus_salivarius	99.8319	1786862	97
CCuG47825	unclassified	0.114757	2054	3
CCuG47825	Streptococcus_dysgalactiae_group	0.0323487	579	2
CCuG47825	Citrobacter_rodentium_ICC168	0.0209512	375	1
CCuG47826	Lactobacillus_salivarius	99.1878	1940444	50
CCuG47826	unclassified	0.783915	15336	5
CCuG47826	Lactobacillus_sanfranciscensis	0.0221332	433	3
CCuG47826	Lactobacillus_casei_group	0.00618504	121	1
CECT5713	Lactobacillus_salivarius	100	2136138	4
cp400	Lactobacillus_salivarius	98.7922	2016437	72
cp400	Lactobacillus_reuteri	1.20779	24652	2
DSM18933	unclassified	93.4389	6964	18
DSM18933	Lactobacillus_salivarius	6.56112	489	3
DSM20492	Lactobacillus_salivarius	100	1890069	30
DSM20554	Lactobacillus_salivarius	100	1949872	51
DSM20555	Lactobacillus_salivarius	100	1874089	44

GJ24	Lactobacillus_salivarius	99.7466	1986988	8
GJ24	unclassified	0.253359	5047	1
gul1	Lactobacillus_salivarius	100	1907028	62
gul2	Lactobacillus_salivarius	99.982	1908848	68
gul2	unclassified	0.011628	222	1
gul2	Lactobacillus_johnsonii	0.00633776	121	1
JCM1040	Lactobacillus_salivarius	98.5093	1822228	46
JCM1040	unclassified	1.48416	27454	7
JCM1040	Lactobacillus_casei_group	0.00654124	121	1
JCM1042	Lactobacillus_salivarius	100	1789991	54
JCM1044	Lactobacillus_salivarius	100	1788967	53
JCM1045	Lactobacillus_salivarius	100	1928808	64
JCM1046	Lactobacillus_salivarius	94.2248	1802918	91
JCM1046	unclassified	4.7194	90302	6
JCM1046	Lactobacillus_reuteri	1.0558	20202	3
JCM1047	Lactobacillus_salivarius	98.613	2040949	81
JCM1047	unclassified	1.38704	28707	1
JCM1230	Lactobacillus_salivarius	100	1656507	38
L21	Lactobacillus_salivarius	99.7884	1927838	60
L21	unclassified	0.195349	3774	5
L21	Lactobacillus_sanfranciscensis	0.0162532	314	2
LMG14476	Lactobacillus_salivarius	100	1887993	157
LMG14477	Lactobacillus_salivarius	99.99	1822244	144
LMG14477	unclassified	0.00998669	182	1
NCIMB702343	Lactobacillus_salivarius	99.9607	1884140	42
NCIMB702343	unclassified	0.0393128	741	2
NCIMB8816	Lactobacillus_salivarius	99.0933	1820558	61
NCIMB8816	unclassified	0.785101	14424	7
NCIMB8816	Lactobacillus_casei_group	0.121597	2234	1
NCIMB8817	Lactobacillus_salivarius	100	1814802	45
NCIMB8818	Lactobacillus_salivarius	97.8194	1970529	67
NCIMB8818	unclassified	1.99051	40098	9
NCIMB8818	Lactobacillus_casei_group	0.120231	2422	2
NCIMB8818	Streptococcus_anginosus_group	0.0698948	1408	1
NIAS840	Lactobacillus_salivarius	100	1866462	3
Ren	Lactobacillus_salivarius	100	1978364	3
SMXD51	Lactobacillus_salivarius	98.4374	1928066	8
SMXD51	unclassified	1.56264	30607	1
UCC118	Lactobacillus_salivarius	100	2133977	4

Table S5

	UCC118 +	UCC118 -	CECT5713 +	CECT5713 -	NIAS840 +	NIAS840 -	Ren +	Ren -
chromosome	100	0	100	0	100	0	100	0
megaplasmid 1	100	0	100	0	97.5	2.5	85.3	14.7
megaplasmid 2	NA	NA	NA	NA	8.3	91.7	NA	NA
small plasmid 1	100	0	100	0	0	100	90.7	9.3
small plasmid 2	100	0	100	0	NA	NA	NA	NA

Table S6

Aspartic (A) Peptidases

FAMILY	SUBFAMILY	TYPE ENZYME
A1	A1A	pepsin A (Homo sapiens)
	A1B	nepenthesin (Nepenthes gracilis)
A2	A2A	HIV-1 retropepsin (human immunodeficiency virus 1)
	A2B	Ty3 transposon peptidase (Saccharomyces cerevisiae)
	A2C	Gypsy transposon peptidase (Drosophila melanogaster)
	A2D	Osvaldo retrotransposon peptidase (Drosophila buzzatii)
A3	A3A	cauliflower mosaic virus-type peptidase (cauliflower mosaic virus)
	A3B	bacilliform virus peptidase (rice tungro bacilliform virus)
A5		thermopsin (Sulfolobus acidocaldarius)
A8		signal peptidase II (Escherichia coli)
A9		spumapepsin (human spumaretrovirus)
A11	A11A	Copia transposon peptidase (Drosophila melanogaster)
	A11B	Ty1 transposon peptidase (Saccharomyces cerevisiae)
A22	A22A	presenilin 1 (Homo sapiens)
	A22B	impas 1 peptidase (Homo sapiens)
A24	A24A	type 4 prepilin peptidase 1 (Pseudomonas aeruginosa)
	A24B	FlaK peptidase (Methanococcus maripaludis)
A25		gpr peptidase (Bacillus megaterium)
A26		omptin (Escherichia coli)
A28	A28A	DNA-damage inducible protein 1 (Saccharomyces cerevisiae)
	A28B	skin SASPase (Mus musculus)
A31		HybD peptidase (Escherichia coli)
A32		PerP peptidase (Caulobacter crescentus)
A36		sporulation factor SpoIIGA (Bacillus subtilis)

Cysteine (C) Peptidases

FAMILY	SUBFAMILY	TYPE ENZYME
C1	C1A	papain (Carica papaya)
	C1B	bleomycin hydrolase (Saccharomyces cerevisiae)
C2	C2A	calpain-2 (Homo sapiens)
C3	C3A	poliovirus-type picornain 3C (human poliovirus 1)
	C3B	enterovirus picornain 2A (human poliovirus 1)
	C3C	foot-and-mouth disease virus picornain 3C (foot-and-mouth disease virus)
	C3D	cowpea mosaic comovirus-type picornain 3C (cowpea mosaic virus)
	C3E	hepatitis A virus-type picornain 3C (hepatitis A virus)
	C3F	parechovirus picornain 3C (human parechovirus 1)
	C3G	rice tungro spherical virus-type peptidase (rice tungro spherical virus)
	C3H	grapevine fanleaf-type nepovirus picornain 3C (grapevine fanleaf virus)
C4		nuclear-inclusion-a peptidase (plum pox virus)
C5		adenain (human adenovirus type 2)
C6		potato virus Y-type helper component peptidase (potato virus Y)
C7		chestnut blight fungus virus p29 peptidase (Cryphonectria hypovirus)
C8		chestnut blight fungus virus p48 peptidase (Cryphonectria hypovirus 1)
C9		sindbis virus-type nsP2 peptidase (Sindbis virus)
C10		streptopain (Streptococcus pyogenes)
C11		clostripain (Clostridium histolyticum)
C12		ubiquitinyl hydrolase-L1 (Homo sapiens)
C13		legumain (Canavalia ensiformis)
C14	C14A	caspase-1 (Rattus norvegicus)
	C14B	metacaspase Yca1 (Saccharomyces cerevisiae)
C15		pyroglutamyl-peptidase I (Bacillus amyloliquefaciens)
C16	C16A	murine hepatitis coronavirus papain-like peptidase 1 (murine hepatitis virus)
	C16B	murine hepatitis coronavirus papain-like peptidase 2 (murine hepatitis virus)
C18		hepatitis C virus peptidase 2 (hepatitis C virus)
C19		ubiquitin-specific peptidase 14 (Homo sapiens)
C21		tymovirus peptidase (turnip yellow mosaic virus)
C23		carlavirus peptidase (apple stem pitting virus)
C24		rabbit hemorrhagic disease virus 3C-like peptidase (rabbit hemorrhagic disease virus)
C25		gingipain RgpA (Porphyromonas gingivalis)
C26		gamma-glutamyl hydrolase (Rattus norvegicus)
C27		rubella virus peptidase (Rubella virus)
C28		foot-and-mouth disease virus L-peptidase (foot-and-mouth disease virus)
C30		porcine transmissible gastroenteritis virus-type main peptidase (transmissible gastroenteritis virus)
C31		porcine reproductive and respiratory syndrome arterivirus-type cysteine peptidase alpha (lactate-dehydrogenase-elevating virus)
C32		equine arteritis virus-type cysteine peptidase (porcine reproductive and respiratory syndrome virus)
C33		equine arteritis virus Nsp2-type cysteine peptidase (equine arteritis virus)
C36		beet necrotic yellow vein furovirus-type papain-like peptidase (beet necrotic yellow vein virus)
C37		calicivirin (Southampton virus)
C39		bacteriocin-processing peptidase (Pediococcus acidilactici)
C40		dipeptidyl-peptidase VI (Lysinibacillus sphaericus)
C42		beet yellows virus-type papain-like peptidase (beet yellows virus)
C44		amidophosphoribosyltransferase precursor (Homo sapiens)
C45		acyl-coenzyme A:6-aminopenicillanic acid acyl-transferase precursor (Penicillium chrysogenum)
C46		hedgehog protein (Drosophila melanogaster)
C47		staphopain A (Staphylococcus aureus)
C48		Ulp1 peptidase (Saccharomyces cerevisiae)
C50		separase (Saccharomyces cerevisiae)
C51		D-alanyl-glycyl peptidase (Staphylococcus aureus)
C53		pestivirus Npro peptidase (classical swine fever virus)
C54		autophagin-1 (Homo sapiens)
C55		YopJ protein (Yersinia pseudotuberculosis)
C56		PfpI peptidase (Pyrococcus furiosus)
C57		vaccinia virus I7L processing peptidase (Vaccinia virus)
C58	C58A	YopT peptidase (Yersinia pestis)
	C58B	HopN1 peptidase (Pseudomonas syringae)
C59		penicillin V acylase precursor (Lysinibacillus sphaericus)
C60	C60A	sortase A (Staphylococcus aureus)
	C60B	sortase B (Staphylococcus aureus)
C62		gill-associated virus 3C-like peptidase (gill-associated virus)
C63		African swine fever virus processing peptidase (African swine fever virus)
C64		Cezanne peptidase (Homo sapiens)
C65		otubain-1 (Homo sapiens)
C66		IdeS peptidase (Streptococcus pyogenes)
C67		CylD peptidase (Homo sapiens)
C69		dipeptidase A (Lactobacillus helveticus)

C70		AvrRpt2 peptidase (Pseudomonas syringae)
C71		pseudomurein endoisopeptidase Pei (Methanobacterium phage psiM2)
C74		pestivirus NS2 peptidase (bovine viral diarrhea virus 1)
C75		AgrB peptidase (Staphylococcus aureus)
C76		viral tegument protein deubiquitinylating peptidase (human herpesvirus 1)
C78		UfSP1 peptidase (Mus musculus)
C79		ElaD peptidase (Escherichia coli)
C80		RTX self-cleaving toxin (Vibrio cholerae)
C82	C82A	L,D-transpeptidase (Enterococcus faecium)
C83		gamma-glutamylcysteine dipeptidyltranspeptidase (Nostoc sp. PCC 7120)
C84		prtH peptidase (Tannerella forsythia)
C85	C85A	OTLD1 deubiquitinylating enzyme (Homo sapiens)
	C85B	OTU1 peptidase (Saccharomyces cerevisiae)
C86		ataxin-3 (Homo sapiens)
C87		nairovirus deubiquitinylating peptidase (Crimean-Congo hemorrhagic fever virus)
C89		acid ceramidase precursor (Homo sapiens)
C93		LapG peptidase (Pseudomonas fluorescens)
C95		lysosomal 66.3 kDa protein (Mus musculus)
C96		McjB peptidase (Escherichia coli)
C97		DeSI-1 peptidase (Mus musculus)
C98		USPL1 peptidase (Homo sapiens)
C99		iflavirus processing peptidase (Ectropis obliqua picorna-like virus)
C101		OTULIN peptidase (Homo sapiens)
C102		GtgE peptidase (Salmonella enterica)
C104		PlyC phage lysin (Streptococcus phage C1)
C105		papain-like peptidase 1 alpha (simian hemorrhagic fever virus)
C107		alphamesonivirus 3C-like peptidase (Cavally virus)
C108		Prp peptidase (Staphylococcus aureus)
C110		kyphoscoliosis peptidase (Mus musculus)
C111		coagulation factor XIIIa (Homo sapiens)
C113		IgdE peptidase (Streptococcus suis)

Glutamic (G) Peptidases

FAMILY	SUBFAMILY	TYPE ENZYME
G1		scytalidoglutamic peptidase (Scytalidium lignicolum)
G2		pre-neck appendage protein (bacteriophage phi-29)

Metallo (M) Peptidases

FAMILY	SUBFAMILY	TYPE ENZYME
M1		aminopeptidase N (Homo sapiens)
M2		angiotensin-converting enzyme peptidase unit 1 (Homo sapiens)
M3	M3A	thimet oligopeptidase (Rattus norvegicus)
	M3B	oligopeptidase F (Lactococcus lactis)
M4		thermolysin (Bacillus thermoproteolyticus)
M5		mycolysin (Streptomyces cacaoi)
M6		immune inhibitor A peptidase (Bacillus thuringiensis)
M7		snapalysin (Streptomyces lividans)
M8		leishmanolysin (Leishmania major)
M9	M9A	bacterial collagenase V (Vibrio alginolyticus)
	M9B	bacterial collagenase H (Clostridium histolyticum)
M10	M10A	matrix metallopeptidase-1 (Homo sapiens)
	M10B	serralysin (Serratia marcescens)
	M10C	fragilysin (Bacteroides fragilis)
M11		gametolysin (Chlamydomonas reinhardtii)
M12	M12A	astacin (Astacus astacus)
	M12B	adamalysin (Crotalus adamanteus)
M13		neprilysin (Homo sapiens)
M14	M14A	carboxypeptidase A1 (Homo sapiens)
	M14B	carboxypeptidase E (Bos taurus)
	M14C	gamma-D-glutamyl--meso-diaminopimelate peptidase I (Lysinibacillus sphaericus)
	M14D	cytosolic carboxypeptidase 6 (Homo sapiens)
M15	M15A	zinc D-Ala-D-Ala carboxypeptidase (Streptomyces albus)
	M15B	vanY D-Ala-D-Ala carboxypeptidase (Enterococcus faecium)
	M15C	Ply118 L-Ala-D-Glu peptidase (bacteriophage A118)
	M15D	vanX D-Ala-D-Ala dipeptidase (Enterococcus faecium)
M16	M16A	pitrilysin (Escherichia coli)
	M16B	mitochondrial processing peptidase beta-subunit (Saccharomyces cerevisiae)
	M16C	eupitrilysin (Homo sapiens)
M17		leucine aminopeptidase 3 (Bos taurus)
M18		aminopeptidase I (Saccharomyces cerevisiae)
M19		membrane dipeptidase (Homo sapiens)
M20	M20A	glutamate carboxypeptidase (Pseudomonas sp.)
	M20B	peptidase T (Escherichia coli)
	M20C	Xaa-His dipeptidase (Escherichia coli)
	M20D	carboxypeptidase Ss1 (Sulfolobus solfataricus)
	M20F	carnosine dipeptidase II (Mus musculus)
M23	M23A	beta-lytic metallopeptidase (Achromobacter lyticus)
	M23B	lysostaphin (Staphylococcus simulans)
M24	M24A	methionyl aminopeptidase 1 (Escherichia coli)
	M24B	aminopeptidase P (Escherichia coli)
M26		IgA1-specific metallopeptidase (Streptococcus sanguinis)
M27		tentoxilysin (Clostridium tetani)
M28	M28A	aminopeptidase S (Streptomyces griseus)
	M28B	glutamate carboxypeptidase II (Homo sapiens)
	M28C	IAP aminopeptidase (Escherichia coli)
	M28D	aminopeptidase ES-62 (Acanthocheilonema viteae)
	M28E	aminopeptidase Ap1 (Vibrio proteolyticus)
	M28F	ywaD peptidase (Bacillus subtilis)
M29		aminopeptidase T (Thermus aquaticus)
M30		hyicolysin (Staphylococcus hyicus)
M32		carboxypeptidase Taq (Thermus aquaticus)
M34		anthrax lethal factor (Bacillus anthracis)
M35		deuterolysin (Aspergillus flavus)
M36		fungalysin (Aspergillus fumigatus)
M38		isoaspartyl dipeptidase (Escherichia coli)
M41		FtsH peptidase (Escherichia coli)
M42		glutamyl aminopeptidase (Lactococcus lactis)
M43	M43A	cytophagalysin (Cytophaga sp.)

	M43B	pappalysin-1 (Homo sapiens)
M44		pox virus metallopeptidase (Vaccinia virus)
M48	M48A	Ste24 peptidase (Saccharomyces cerevisiae)
	M48B	HtpX peptidase (Escherichia coli)
	M48C	Oma1 peptidase (Saccharomyces cerevisiae)
M49		dipeptidyl-peptidase III (Rattus norvegicus)
M50	M50A	site 2 peptidase (Homo sapiens)
	M50B	sporulation factor SpoIVFB (Bacillus subtilis)
M54		archaelysin (Methanocaldococcus jannaschii)
M55		D-aminopeptidase DppA (Bacillus subtilis)
M56		BlaR1 peptidase (Staphylococcus aureus)
M57		prtB g.p. (Myxococcus xanthus)
M60		enhancin (Lymantria dispar nucleopolyhedrovirus)
M61		glycyl aminopeptidase (Sphingomonas capsulata)
M64		IgA peptidase (Clostridium ramosum)
M66		StcE peptidase (Escherichia coli)
M67	M67A	RPN11 peptidase (Saccharomyces cerevisiae)
	M67B	JAMM-like protein (Archaeoglobus fulgidus)
	M67C	STAMBP isopeptidase (Homo sapiens)
M72		peptidyl-Asp metallopeptidase (Pseudomonas aeruginosa)
M73		camelysin (Bacillus cereus)
M74		murein endopeptidase (Escherichia coli)
M75		imelysin (Pseudomonas aeruginosa)
M76		Atp23 peptidase (Homo sapiens)
M77		tryptophanyl aminopeptidase 7-DMATS-type peptidase (Aspergillus fumigatus)
M78		ImmA peptidase (Bacillus subtilis)
M79		RCE1 peptidase (Saccharomyces cerevisiae)
M80		Wss1 peptidase (Saccharomyces cerevisiae)
M81		microcystinase MlrC (Sphingomonas sp. ACM-3962)
M82		PrsW peptidase (Bacillus subtilis)
M84		MpriBi peptidase (Bacillus intermedius)
M85		NleC peptidase (Escherichia coli)
M86		PghP gamma-polyglutamate hydrolase (Bacillus phage phiNIT1)
M87		chloride channel accessory protein 1 (Homo sapiens)
M88		IMPa peptidase (Pseudomonas aeruginosa)
M90		MtfA peptidase (Escherichia coli)
M91		NleD peptidase (Escherichia coli)
M93		BACCAC_01431 g.p. and similar (Bacteroides caccae)
M95		selecase (Methanocaldococcus jannaschii)
M96		Tiki1 peptidase (Homo sapiens)
M97		EcxAB peptidase (Escherichia coli)
M98		YghJ g.p. (Escherichia coli)
M99		Csd4 peptidase (Helicobacter pylori)
Asparagine (N) Peptide Lyases		
FAMILY	SUBFAMILY	TYPE ENZYME
N1		nodavirus peptide lyase (flock house virus)
N2		tetravirus coat protein (Nudaurelia capensis omega virus)
N4		Tsh-associated self-cleaving domain and similar (Escherichia coli)
N5		picobirnavirus self-cleaving protein (Human picobirnavirus)
N6		YscU protein (Yersinia pseudotuberculosis)
N7		reovirus type 1 coat protein (Mammalian orthoreovirus 1)
N8		poliovirus capsid VP0-type self-cleaving protein (human poliovirus 1)
N9		intein-containing V-type proton ATPase catalytic subunit A (Saccharomyces cerevisiae)
N10		intein-containing replicative DNA helicase precursor (Synechocystis sp. PCC 6803)
N11		intein-containing chloroplast ATP-dependent peptide lyase (Chlamydomonas eugametos)
Mixed (P) Peptidases		
FAMILY	SUBFAMILY	TYPE ENZYME
P1		DmpA aminopeptidase (Ochrobactrum anthropi)
P2	P2A	EGF-like module containing mucin-like hormone receptor-like 2 (Homo sapiens)
	P2B	polycystin-1 (Homo sapiens)
Serine (S) Peptidases		
FAMILY	SUBFAMILY	TYPE ENZYME
S1	S1A	chymotrypsin A (Bos taurus)
	S1B	glutamyl endopeptidase I (Staphylococcus aureus)
	S1C	DegP peptidase (Escherichia coli)
	S1D	lysyl endopeptidase (Achromobacter lyticus)
	S1E	streptogrisin A (Streptomyces griseus)
	S1F	astrovirus serine peptidase (Mamastrovirus 1)
S3		togavirin (Sindbis virus)
S6		IgA1-specific serine peptidase (Neisseria gonorrhoeae)
S7		flavivirin (yellow fever virus)
S8	S8A	subtilisin Carlsberg (Bacillus licheniformis)
	S8B	kexin (Saccharomyces cerevisiae)
S9	S9A	prolyl oligopeptidase (Sus scrofa)
	S9B	dipeptidyl-peptidase IV (Homo sapiens)
	S9C	acylaminoacyl-peptidase (Homo sapiens)
	S9D	glutamyl endopeptidase C (Arabidopsis thaliana)
S10		carboxypeptidase Y (Saccharomyces cerevisiae)
S11		D-Ala-D-Ala carboxypeptidase A (Geobacillus stearothermophilus)
S12		D-Ala-D-Ala carboxypeptidase B (Streptomyces lividans)
S13		D-Ala-D-Ala peptidase C (Escherichia coli)
S14		peptidase Clp (Escherichia coli)
S15		Xaa-Pro dipeptidyl-peptidase (Lactococcus lactis)
S16		Lon-A peptidase (Escherichia coli)
S21		cytomegalovirus assemblin (human herpesvirus 5)
S24		repressor LexA (Escherichia coli)
S26	S26A	signal peptidase I (Escherichia coli)
	S26B	signalase 21 kDa component (Saccharomyces cerevisiae)
	S26C	TraF peptidase (Escherichia coli)
S28		lysosomal Pro-Xaa carboxypeptidase (Homo sapiens)
S29		hepacivirin (hepatitis C virus)
S30		potyvirus P1 peptidase (plum pox virus)
S31		pestivirus NS3 polyprotein peptidase (bovine viral diarrhea virus 1)
S32		equine arteritis virus serine peptidase (equine arteritis virus)
S33		prolyl aminopeptidase (Neisseria gonorrhoeae)
S37		PS-10 peptidase (Streptomyces lividans)

S39	S39A	sobemovirus peptidase (cocksfoot mottle virus)
	S39B	luteovirus peptidase (potato leaf roll luteovirus)
S41	S41A	C-terminal processing peptidase-1 (Escherichia coli)
	S41B	tricorn core peptidase (Thermoplasma acidophilum)
S45		penicillin G acylase precursor (Escherichia coli)
S46		dipeptidyl-peptidase 7 (Porphyromonas gingivalis)
S48		HetR putative peptidase (Anabaena variabilis)
S49	S49A	signal peptide peptidase A (Escherichia coli)
	S49B	protein C (bacteriophage lambda)
	S49C	archaean signal peptide peptidase 1 (Pyrococcus horikoshii)
S50		infectious pancreatic necrosis birnavirus Vp4 peptidase (infectious pancreatic necrosis virus)
S51		dipeptidase E (Escherichia coli)
S53		sedolisin (Pseudomonas sp. 101)
S54		rhomboid-1 (Drosophila melanogaster)
S55		SpoIVB peptidase (Bacillus subtilis)
S59		nucleoporin 145 (Homo sapiens)
S60		lactoferrin (Homo sapiens)
S62		influenza A PA peptidase (influenza A virus)
S64		Ssy5 peptidase (Saccharomyces cerevisiae)
S65		picornain-like cysteine peptidase (Breda virus)
S66		murein tetrapeptidase LD-carboxypeptidase (Pseudomonas aeruginosa)
S68		PIDD auto-processing protein unit 1 (Homo sapiens)
S69		Tellina virus 1 VP4 peptidase (Tellina virus 1)
S71		MUC1 self-cleaving mucin (Homo sapiens)
S72		dystroglycan (Homo sapiens)
S73		gpO peptidase (Enterobacteria phage P2)
S74		Escherichia coli phage K1F endosialidase CIMCD self-cleaving protein (Enterobacteria phage K1F)
S75		White bream virus serine peptidase (White bream virus)
S77		prohead peptidase gp21 (Enterobacteria phage T4)
S78		prohead peptidase (Enterobacteria phage HK97)
S79		CARD8 self-cleaving protein (Homo sapiens)
S80		prohead peptidase gp175 (Pseudomonas phage phiKZ)
S81		destabilase (Hirudo medicinalis)
Threonine (T) Peptidases		
FAMILY	SUBFAMILY	TYPE ENZYME
T1	T1A	archaean proteasome, beta component (Thermoplasma acidophilum)
	T1B	HslV component of HslUV peptidase (Escherichia coli)
T2		glycosylasparaginase precursor (Homo sapiens)
T3		gamma-glutamyltransferase 1 (Escherichia coli)
T5		ornithine acetyltransferase precursor (Saccharomyces cerevisiae)
T7		CwpV self-cleaving threonine peptidase (Peptoclostridium difficile)
Peptidases of Unknown Catalytic Type		
FAMILY	SUBFAMILY	TYPE ENZYME
U32		collagenase (Porphyromonas gingivalis)
U40		protein P5 murein endopeptidase (bacteriophage phi-6)
U49		Lit peptidase (Escherichia coli)
U56		homomultimeric peptidase (Thermotoga maritima)
U57		yabG protein (Bacillus subtilis)
U62		microcin-processing peptidase 1 (Escherichia coli)
U69		AIDA-I self-cleaving autotransporter protein (Escherichia coli)
U72		Dop isopeptidase (Mycobacterium tuberculosis)
U73		small protease (Pseudomonas aeruginosa)

Table S7

strain	replicon	CRISPR type	CRISPR sub-type	repeat number	repeat length	repeat sequence	avg. length of spacers	cas 1	cas 3	cas 9	cas 10
778	chromosome	II	II-A	7	34	GTTTCAGAAGGATGTTAAATCAATTAGGTTAAGA	32	Y		Y	
866	chromosome	II	II-A	59	36	GTTTCAGAAGGATGTTAAATCAATTAGGTTAAGACT	30	Y		Y	
866	chromosome	III	III-A	19	36	GTTTTCGTCTCCTATATTCGGAGATATGTTCTTACT	38	Y			Y
01M14315	chromosome	II	II-A	12	36	GTTTCAGAAGTATGTTAAATCAATTAGGTTAAGACC	30	Y		Y	
01M14315	chromosome	III	III-A	13	36	GTTTTCGTCTCCTATATTCGGAGATATGTTCTTACT	39	Y			Y
ACS116	chromosome	II	II-A	17	36	GTTTCAGAAGGATGTTAAATCAATTAGGTTAAGACT	29	Y		Y	
ACS116	chromosome	III	III-A	9	35	TTTTCGTCTCCTATATTCGGAGATATGTTCTTACT	38	Y			
AH4231	chromosome	II	II-A	60	36	GTTTCAGAAGGATGTTAAATCAATTAGGTTAAGACT	30	Y		Y	
AH4231	chromosome	III	III-A	26	36	GTTTTCGTCTCCTATATTCGGAGATATGTTCTTACT	38	Y			Y
AH4331	chromosome	II	II-A	60	36	GTTTCAGAAGGATGTTAAATCAATTAGGTTAAGACT	30	Y		Y	
AH4331	chromosome	III	III-A	26	36	GTTTTCGTCTCCTATATTCGGAGATATGTTCTTACT	38	Y			Y
AH43324	chromosome	II	II-A	28	36	GTTTCAGAAGTATGTTAAATCAATAAGGTTAAGACC	30	Y		Y	
AH43348	chromosome	II	II-A	62	36	GTTTCAGAAGGATGTTAAATCAATTAGGTTAAGACT	30	Y		Y	
AH43348	chromosome	III	III-A	31	36	GTTTTCGTCTCCTATATTCGGAGATATGTTCTTACT	38	Y			Y
ATCC11741	chromosome	III	III-A	12	36	GTTTTCGTCTCCTTCATTCGGAGATATGTTCTTATT	37	Y			Y
CCuG2753OB	chromosome	II	II-A	19	36	GTTTCAGAAGTATGTTAAATCAATTAGGTTAAGACC	30	Y		Y	
CCuG38008	chromosome	II	II-A	29	36	GTTTCAGAAGGATGTTAAATCAATTAGGTTAAGACC	30	Y		Y	
CCuG44481	NA	NA	NA	0	0	NA	0				
CCuG45735	chromosome	II	II-A	40	36	GTTTCAGAAGGATGTTAAATCAATTAGGTTAAGACT	30	Y		Y	
CCuG45735	chromosome	III	III-A	19	36	GTTTTCGTCTCCTATATTCGGAGATATGTTCTTACT	38	Y			Y
CCuG47171	chromosome	III	III-A	11	36	GTTTTCGTCTCCTTTATTCGGAGATATGTTCTTACT	37	Y			Y
CCuG47825	chromosome	II	II-A	23	36	GTTTCAGAAGTATGTTAAATCAATTAGGTTAAGACC	30	Y		Y	
CCuG47825	chromosome	undefined	undefined	12	37	AGTTTTCGTCTCCTATATTCGGAGATATGTTCTTACT	40				
CCuG47826	chromosome	II	II-A	22	36	GTTTCAGAAGTATGTTAAATCAATTAGGTTAAGACC	30	Y		Y	
CCuG47826	chromosome	III	III-A	19	36	GTTTTCGTCTCCTATATTCGGAGATATGTTCTTACT	37	Y			Y
CECT5713	chromosome	II	II-A	28	36	GTTTCAGAAGTATGTTAAATCAATAAGGTTAAGACC	30	Y		Y	
cp400	chromosome	III	III-A	28	36	GTTTTCGTCTCCTATATTCGGAGATATGTTCTTACT	38	Y			Y
DSM18933	NA	NA	NA	0	0	NA	0				
DSM20492	chromosome	II	II-A	17	36	GTTTCAGAAGTATGTTAAATCAATTAGGTTAAGACC	30	Y		Y	
DSM20492	chromosome	III	III-A	22	36	GTTTTCGTCTCCTATATTCGGAGATATGTTCTTACT	38	Y			Y
DSM20554	chromosome	III	III-A	22	36	GTTTTCGTCTCCTATATTCGGAGATATGTTCTTACT	37	Y			Y
DSM20555	chromosome	III	III-A	12	36	GTTTTCGTCTCCTTCATTCGGAGATATGTTCTTATT	37	Y			Y
GJ24	NA	NA	NA	0	0	NA	0				
gul1	chromosome	III	III-A	12	36	GTTTTCGTCTCCTTCATTCGGAGATATGTTCTTATT	37	Y			Y
gul2	chromosome	III	III-A	12	36	GTTTTCGTCTCCTTCATTCGGAGATATGTTCTTATT	37	Y			Y
JCM1040	chromosome	II	II-A	15	36	GTTTCAGAAGTATGTTAAATCAATTAGGTTAAGACC	29	Y		Y	
JCM1040	chromosome	III	III-A	18	36	GTTTTCGTCTCCTATATTCGGAGATATGTTCTTACT	38	Y			Y
JCM1042	chromosome	II	II-A	19	36	GTTTCAGAAGTATGTTAAATCAATTAGGTTAAGACC	30	Y		Y	
JCM1044	chromosome	II	II-A	19	36	GTTTCAGAAGTATGTTAAATCAATTAGGTTAAGACC	30	Y		Y	
JCM1045	NA	NA	NA	0	0	NA	0				
JCM1046	chromosome	III	III-A	27	36	GTTTTCGTCTCCTATATTCGGAGATATGTTCTTACT	38	Y			Y
JCM1047	NA	NA	NA	0	0	NA	0				
JCM1230	chromosome	II	II-A	40	36	GTTTCAGAAGTATGTTAAATCAATTAGGTTAAGGCC	29	Y		Y	
L21	chromosome	II	II-A	26	36	GTTTCAGAAGTATGTTAAATCAATTAGGTTAAGACC	29	Y		Y	
L21	chromosome	III	III-A	50	36	GTTTTCGTCTCCTATATTCGGAGATATGTTCTTACT	37	Y			Y
LMG14476	NA	NA	NA	0	0	NA	0				
LMG14477	NA	NA	NA	0	0	NA	0				
NCIMB702343	chromosome	II	II-A	41	36	GTTTCAGAAGTATGTTAAATCAATTAGGTTAAGACC	30	Y		Y	
NCIMB702343	chromosome	undescribed	undescribed	11	24	GTTTCAGAAGTATGTTAAATCAAT	41				
NCIMB702343	chromosome	III	III-A	20	36	GTTTTCGTCTCCTATATTCGGAGATATGTTCTTACT	37	Y			Y
NCIMB702343	chromosome	undescribed	undescribed	6	35	GTTTCAGAAGTATGTTAAATCAATTAGGTTAAGAC	31				
NCIMB8816	chromosome	II	II-A	8	36	GTTTCAGAAGTATGTTAAATCAATTAGGTTAAGACC	30	Y		Y	
NCIMB8817	chromosome	II	II-A	50	36	GTTTCAGAAGTATGTTAAATCAATTAGGTTAAGACC	30	Y		Y	
NCIMB8818	chromosome	II	II-A	8	36	GTTTCAGAAGTATGTTAAATCAATAAGGTTAAGACC	30	Y		Y	
NIAS840	chromosome	undefined	undefined	18	31	TCAAGTTCCTTAAGTGAAAGCTTGAGTACAT	40				
NIAS840	chromosome	III	III-A	9	36	GTTTTCGTCTCCTATATTCGGAGATATGTTCTTACT	36	Y			Y
NIAS840	megaplasmid	undefined	undefined	5	37	GTCTTACACCTATGTCAATTCAACTAGGTTCAGAACC	29				
Ren	chromosome	II	II-A	11	36	GTTTCAGAAGTATGTTAAATCAATTAGGTTAAGACC	29	Y			Y
Ren	chromosome	III	III-A	18	36	GTTTTCGTCTCCTATATTCGGAGATATGTTCTTACT	37				
SMXD51	chromosome	II	II-A	25	36	GTTTCAGAAGTATGTTAAATCAATAAGGTTAAGACC	29	Y		Y	
UCC118	chromosome	II	II-A	28	36	GTTTCAGAAGTATGTTAAATCAATAAGGTTAAGACC	30	Y		Y	

Table S8

query gene	% identity	query coverage	e-value	query gene length	functional annotation
01M14315_ORF_49	45	225	1.00E-62	236	vanRM is a vanR variant found in the vanM gene cluster
01M14315_ORF_69	49	498	2.00E-170	493	LsaA is an ABC efflux pump expressed in Enterococcus faecalis
01M14315_ORF_212	48	233	2.00E-70	229	vanRF is a vanR variant found in the vanF gene cluster
01M14315_ORF_611	46	418	8.00E-123	440	Mycobacterium tuberculosis murA confers intrinsic resistance to fosfomycin
01M14315_ORF_653	69	396	0	395	Sequence variants of Streptomyces cinnamoneus elongation factor Tu that confer resistance to elfamycin antibiotics
01M14315_ORF_936	45	648	0	772	PBP1a is a penicillin-binding protein found in Streptococcus pneumoniae
01M14315_ORF_940	41	569	4.00E-147	578	AdeC is the outer membrane factor of the AdeABC multidrug efflux complex
01M14315_ORF_1128	41	233	3.00E-54	233	vanRE is a vanR variant found in the vanE gene cluster
01M14315_ORF_1278	62	61	2.00E-22	73	VanU is a transcriptional activator of the vanG operon of vancomycin resistance genes
01M14315_ORF_1354	40	222	7.00E-47	224	MacB is an ATP-binding cassette (ABC) transporter that exports macrolides with 14- or 15- membered lactones
01M14315_ORF_1416	44	222	4.00E-62	227	MacB is an ATP-binding cassette (ABC) transporter that exports macrolides with 14- or 15- membered lactones
01M14315_ORF_1628	43	219	2.00E-53	233	MacB is an ATP-binding cassette (ABC) transporter that exports macrolides with 14- or 15- membered lactones
778_ORF_53	45	225	1.00E-62	236	vanRM is a vanR variant found in the vanM gene cluster
778_ORF_72	50	500	2.00E-172	493	LsaA is an ABC efflux pump expressed in Enterococcus faecalis
778_ORF_226	48	233	1.00E-70	229	vanRF is a vanR variant found in the vanF gene cluster
778_ORF_512	46	418	1.00E-120	440	Mycobacterium tuberculosis murA confers intrinsic resistance to fosfomycin
778_ORF_610	69	396	0	395	Sequence variants of Streptomyces cinnamoneus elongation factor Tu that confer resistance to elfamycin antibiotics
778_ORF_828	40	573	2.00E-146	578	AdeC is the outer membrane factor of the AdeABC multidrug efflux complex
778_ORF_832	45	648	0	772	PBP1a is a penicillin-binding protein found in Streptococcus pneumoniae
778_ORF_1079	41	233	3.00E-54	233	vanRE is a vanR variant found in the vanE gene cluster
778_ORF_1272	40	222	8.00E-47	224	MacB is an ATP-binding cassette (ABC) transporter that exports macrolides with 14- or 15- membered lactones
778_ORF_1335	44	222	1.00E-61	227	MacB is an ATP-binding cassette (ABC) transporter that exports macrolides with 14- or 15- membered lactones
778_ORF_1549	43	221	6.00E-56	233	MacB is an ATP-binding cassette (ABC) transporter that exports macrolides with 14- or 15- membered lactones
866_ORF_51	45	225	1.00E-62	236	vanRM is a vanR variant found in the vanM gene cluster
866_ORF_106	55	95	7.00E-31	99	LsaA is an ABC efflux pump expressed in Enterococcus faecalis
866_ORF_107	49	391	5.00E-131	387	LsaA is an ABC efflux pump expressed in Enterococcus faecalis
866_ORF_271	48	233	1.00E-70	229	vanRF is a vanR variant found in the vanF gene cluster
866_ORF_642	46	418	1.00E-120	441	Mycobacterium tuberculosis murA confers intrinsic resistance to fosfomycin
866_ORF_682	69	396	0	395	Sequence variants of Streptomyces cinnamoneus elongation factor Tu that confer resistance to elfamycin antibiotics
866_ORF_913	40	583	9.00E-146	578	AdeC is the outer membrane factor of the AdeABC multidrug efflux complex
866_ORF_917	45	648	0	772	PBP1a is a penicillin-binding protein found in Streptococcus pneumoniae
866_ORF_1176	41	233	6.00E-54	233	vanRE is a vanR variant found in the vanE gene cluster
866_ORF_1362	40	219	1.00E-46	224	MacB is an ATP-binding cassette (ABC) transporter that exports macrolides with 14- or 15- membered lactones
866_ORF_1373	45	302	2.00E-80	302	bcrA is an ABC transporter found in Bacillus licheniformis that confers bacitracin resistance
866_ORF_1455	44	222	4.00E-62	227	MacB is an ATP-binding cassette (ABC) transporter that exports macrolides with 14- or 15- membered lactones
866_ORF_1663	43	219	4.00E-53	233	MacB is an ATP-binding cassette (ABC) transporter that exports macrolides with 14- or 15- membered lactones
ACS116_ORF_52	45	225	1.00E-62	236	vanRM is a vanR variant found in the vanM gene cluster
ACS116_ORF_71	55	95	7.00E-31	99	LsaA is an ABC efflux pump expressed in Enterococcus faecalis
ACS116_ORF_72	49	391	5.00E-131	387	LsaA is an ABC efflux pump expressed in Enterococcus faecalis
ACS116_ORF_228	48	233	1.00E-70	229	vanRF is a vanR variant found in the vanF gene cluster
ACS116_ORF_651	46	418	1.00E-120	441	Mycobacterium tuberculosis murA confers intrinsic resistance to fosfomycin
ACS116_ORF_722	69	396	0	395	Sequence variants of Streptomyces cinnamoneus elongation factor Tu that confer resistance to elfamycin antibiotics
ACS116_ORF_1019	40	583	9.00E-146	578	AdeC is the outer membrane factor of the AdeABC multidrug efflux complex
ACS116_ORF_1023	45	648	0	772	PBP1a is a penicillin-binding protein found in Streptococcus pneumoniae
ACS116_ORF_1271	40	187	3.00E-41	191	vanRA, also known as vanR, is a vanR variant found in the vanA gene cluster
ACS116_ORF_1293	40	223	2.00E-49	223	vanRE is a vanR variant found in the vanE gene cluster
ACS116_ORF_1328	45	286	1.00E-80	299	MprF is a integral membrane protein that modifies the negatively-charged phosphatidylglycerol on the membrane surface
ACS116_ORF_1482	40	219	1.00E-46	224	MacB is an ATP-binding cassette (ABC) transporter that exports macrolides with 14- or 15- membered lactones
ACS116_ORF_1493	45	302	2.00E-80	302	bcrA is an ABC transporter found in Bacillus licheniformis that confers bacitracin resistance
ACS116_ORF_1575	44	222	4.00E-62	227	MacB is an ATP-binding cassette (ABC) transporter that exports macrolides with 14- or 15- membered lactones
ACS116_ORF_1792	43	219	4.00E-53	233	MacB is an ATP-binding cassette (ABC) transporter that exports macrolides with 14- or 15- membered lactones
AH4231_ORF_45	45	225	1.00E-62	236	vanRM is a vanR variant found in the vanM gene cluster
AH4231_ORF_65	55	95	7.00E-31	99	LsaA is an ABC efflux pump expressed in Enterococcus faecalis
AH4231_ORF_66	49	391	5.00E-131	387	LsaA is an ABC efflux pump expressed in Enterococcus faecalis
AH4231_ORF_231	48	233	1.00E-70	229	vanRF is a vanR variant found in the vanF gene cluster
AH4231_ORF_639	46	418	1.00E-120	441	Mycobacterium tuberculosis murA confers intrinsic resistance to fosfomycin
AH4231_ORF_680	69	396	0	395	Sequence variants of Streptomyces cinnamoneus elongation factor Tu that confer resistance to elfamycin antibiotics
AH4231_ORF_1023	40	583	9.00E-146	578	AdeC is the outer membrane factor of the AdeABC multidrug efflux complex
AH4231_ORF_1027	45	648	0	772	PBP1a is a penicillin-binding protein found in Streptococcus pneumoniae
AH4231_ORF_1288	41	233	6.00E-54	233	vanRE is a vanR variant found in the vanE gene cluster
AH4231_ORF_1471	40	219	1.00E-46	224	MacB is an ATP-binding cassette (ABC) transporter that exports macrolides with 14- or 15- membered lactones
AH4231_ORF_1482	45	302	2.00E-80	302	bcrA is an ABC transporter found in Bacillus licheniformis that confers bacitracin resistance
AH4231_ORF_1564	44	222	4.00E-62	227	MacB is an ATP-binding cassette (ABC) transporter that exports macrolides with 14- or 15- membered lactones
AH4231_ORF_1777	43	219	4.00E-53	233	MacB is an ATP-binding cassette (ABC) transporter that exports macrolides with 14- or 15- membered lactones
AH4231_ORF_1862	40	227	1.00E-54	233	vanRM is a vanR variant found in the vanM gene cluster
AH4331_ORF_45	45	225	1.00E-62	236	vanRM is a vanR variant found in the vanM gene cluster
AH4331_ORF_63	55	95	7.00E-31	99	LsaA is an ABC efflux pump expressed in Enterococcus faecalis
AH4331_ORF_64	49	391	5.00E-131	387	LsaA is an ABC efflux pump expressed in Enterococcus faecalis
AH4331_ORF_230	48	233	1.00E-70	229	vanRF is a vanR variant found in the vanF gene cluster
AH4331_ORF_647	46	418	1.00E-120	441	Mycobacterium tuberculosis murA confers intrinsic resistance to fosfomycin
AH4331_ORF_687	69	396	0	395	Sequence variants of Streptomyces cinnamoneus elongation factor Tu that confer resistance to elfamycin antibiotics
AH4331_ORF_994	40	583	9.00E-146	578	AdeC is the outer membrane factor of the AdeABC multidrug efflux complex
AH4331_ORF_998	45	648	0	772	PBP1a is a penicillin-binding protein found in Streptococcus pneumoniae
AH4331_ORF_1263	41	233	6.00E-54	233	vanRE is a vanR variant found in the vanE gene cluster
AH4331_ORF_1446	40	219	1.00E-46	224	MacB is an ATP-binding cassette (ABC) transporter that exports macrolides with 14- or 15- membered lactones
AH4331_ORF_1457	45	302	2.00E-80	302	bcrA is an ABC transporter found in Bacillus licheniformis that confers bacitracin resistance
AH4331_ORF_1539	44	222	4.00E-62	227	MacB is an ATP-binding cassette (ABC) transporter that exports macrolides with 14- or 15- membered lactones
AH4331_ORF_1752	43	219	4.00E-53	233	MacB is an ATP-binding cassette (ABC) transporter that exports macrolides with 14- or 15- membered lactones
AH4331_ORF_1832	40	227	1.00E-54	233	vanRM is a vanR variant found in the vanM gene cluster
AH43324_ORF_52	45	225	1.00E-62	236	vanRM is a vanR variant found in the vanM gene cluster
AH43324_ORF_70	50	498	3.00E-171	493	LsaA is an ABC efflux pump expressed in Enterococcus faecalis
AH43324_ORF_231	48	228	2.00E-70	229	vanRF is a vanR variant found in the vanF gene cluster
AH43324_ORF_630	46	418	2.00E-121	441	Mycobacterium tuberculosis murA confers intrinsic resistance to fosfomycin
AH43324_ORF_670	69	396	0	395	Sequence variants of Streptomyces cinnamoneus elongation factor Tu that confer resistance to elfamycin antibiotics
AH43324_ORF_969	40	583	1.00E-145	578	AdeC is the outer membrane factor of the AdeABC multidrug efflux complex
AH43324_ORF_973	45	648	0	772	PBP1a is a penicillin-binding protein found in Streptococcus pneumoniae
AH43324_ORF_1227	41	233	4.00E-54	233	vanRE is a vanR variant found in the vanE gene cluster
AH43324_ORF_1280	40	400	2.00E-90	415	MprF is a integral membrane protein that modifies the negatively-charged phosphatidylglycerol on the membrane surface
AH43324_ORF_1428	40	222	7.00E-47	224	MacB is an ATP-binding cassette (ABC) transporter that exports macrolides with 14- or 15- membered lactones
AH43324_ORF_1439	45	305	1.00E-79	302	bcrA is an ABC transporter found in Bacillus licheniformis that confers bacitracin resistance
AH43324_ORF_1522	44	222	1.00E-61	227	MacB is an ATP-binding cassette (ABC) transporter that exports macrolides with 14- or 15- membered lactones
AH43324_ORF_1765	43	219	5.00E-53	233	MacB is an ATP-binding cassette (ABC) transporter that exports macrolides with 14- or 15- membered lactones
AH43348_ORF_45	45	225	1.00E-62	236	vanRM is a vanR variant found in the vanM gene cluster
AH43348_ORF_64	55	95	7.00E-31	99	LsaA is an ABC efflux pump expressed in Enterococcus faecalis
AH43348_ORF_65	49	391	5.00E-131	387	LsaA is an ABC efflux pump expressed in Enterococcus faecalis
AH43348_ORF_232	48	233	1.00E-70	229	vanRF is a vanR variant found in the vanF gene cluster
AH43348_ORF_640	46	418	1.00E-120	441	Mycobacterium tuberculosis murA confers intrinsic resistance to fosfomycin
AH43348_ORF_680	69	396	0	395	Sequence variants of Streptomyces cinnamoneus elongation factor Tu that confer resistance to elfamycin antibiotics
AH43348_ORF_992	40	583	9.00E-146	578	AdeC is the outer membrane factor of the AdeABC multidrug efflux complex
AH43348_ORF_996	45	648	0	772	PBP1a is a penicillin-binding protein found in Streptococcus pneumoniae
AH43348_ORF_1259	41	233	6.00E-54	233	vanRE is a vanR variant found in the vanE gene cluster
AH43348_ORF_1447	40	219	1.00E-46	224	MacB is an ATP-binding cassette (ABC) transporter that exports macrolides with 14- or 15- membered lactones
AH43348_ORF_1458	45	302	2.00E-80	302	bcrA is an ABC transporter found in Bacillus licheniformis that confers bacitracin resistance
AH43348_ORF_1539	44	222	4.00E-62	227	MacB is an ATP-binding cassette (ABC) transporter that exports macrolides with 14- or 15- membered lactones
AH43348_ORF_1755	43	219	4.00E-53	233	MacB is an ATP-binding cassette (ABC) transporter that exports macrolides with 14- or 15- membered lactones
ATCC11741_ORF_54	45	225	1.00E-62	236	vanRM is a vanR variant found in the vanM gene cluster
ATCC11741_ORF_71	50	498	3.00E-172	493	LsaA is an ABC efflux pump expressed in Enterococcus faecalis
ATCC11741_ORF_216	48	233	1.00E-70	229	vanRF is a vanR variant found in the vanF gene cluster

ATCC11741_ORF_559	46	418	2.00E-121	441	Mycobacterium tuberculosis murA confers intrinsic resistance to fosfomycin
ATCC11741_ORF_600	69	396	0	395	Sequence variants of Streptomyces cinnamoneus elongation factor Tu that confer resistance to elfamycin antibiotics
ATCC11741_ORF_824	41	573	9.00E-147	578	AdeC is the outer membrane factor of the AdeABC multidrug efflux complex
ATCC11741_ORF_828	45	648	0	772	PBP1a is a penicillin-binding protein found in Streptococcus pneumoniae
ATCC11741_ORF_1079	41	233	4.00E-54	233	vanRE is a vanR variant found in the vanE gene cluster
ATCC11741_ORF_1264	40	222	2.00E-46	224	MacB is an ATP-binding cassette (ABC) transporter that exports macrolides with 14- or 15- membered lactones
ATCC11741_ORF_1327	44	222	2.00E-61	227	MacB is an ATP-binding cassette (ABC) transporter that exports macrolides with 14- or 15- membered lactones
ATCC11741_ORF_1544	43	221	1.00E-56	233	MacB is an ATP-binding cassette (ABC) transporter that exports macrolides with 14- or 15- membered lactones
CCuG2753OB_ORF_53	45	225	1.00E-62	236	vanRM is a vanR variant found in the vanM gene cluster
CCuG2753OB_ORF_73	49	500	1.00E-171	493	LsaA is an ABC efflux pump expressed in Enterococcus faecalis
CCuG2753OB_ORF_328	48	233	1.00E-70	229	vanRF is a vanR variant found in the vanF gene cluster
CCuG2753OB_ORF_506	46	418	2.00E-121	441	Mycobacterium tuberculosis murA confers intrinsic resistance to fosfomycin
CCuG2753OB_ORF_546	69	396	0	395	Sequence variants of Streptomyces cinnamoneus elongation factor Tu that confer resistance to elfamycin antibiotics
CCuG2753OB_ORF_777	40	583	4.00E-146	578	AdeC is the outer membrane factor of the AdeABC multidrug efflux complex
CCuG2753OB_ORF_782	45	648	0	772	PBP1a is a penicillin-binding protein found in Streptococcus pneumoniae
CCuG2753OB_ORF_1031	41	233	3.00E-54	233	vanRE is a vanR variant found in the vanE gene cluster
CCuG2753OB_ORF_1212	40	222	7.00E-47	224	MacB is an ATP-binding cassette (ABC) transporter that exports macrolides with 14- or 15- membered lactones
CCuG2753OB_ORF_1275	44	222	1.00E-61	227	MacB is an ATP-binding cassette (ABC) transporter that exports macrolides with 14- or 15- membered lactones
CCuG2753OB_ORF_1501	43	221	2.00E-56	244	MacB is an ATP-binding cassette (ABC) transporter that exports macrolides with 14- or 15- membered lactones
CCuG38008_ORF_55	45	225	1.00E-62	236	vanRM is a vanR variant found in the vanM gene cluster
CCuG38008_ORF_70	49	500	2.00E-171	493	LsaA is an ABC efflux pump expressed in Enterococcus faecalis
CCuG38008_ORF_221	48	233	1.00E-70	229	vanRF is a vanR variant found in the vanF gene cluster
CCuG38008_ORF_546	46	418	2.00E-121	441	Mycobacterium tuberculosis murA confers intrinsic resistance to fosfomycin
CCuG38008_ORF_586	69	396	0	395	Sequence variants of Streptomyces cinnamoneus elongation factor Tu that confer resistance to elfamycin antibiotics
CCuG38008_ORF_885	40	583	9.00E-147	578	AdeC is the outer membrane factor of the AdeABC multidrug efflux complex
CCuG38008_ORF_889	45	648	0	772	PBP1a is a penicillin-binding protein found in Streptococcus pneumoniae
CCuG38008_ORF_1140	41	233	3.00E-54	233	vanRE is a vanR variant found in the vanE gene cluster
CCuG38008_ORF_1286	60	63	1.00E-22	67	VanU is a transcriptional activator of the vanG operon of vancomycin resistance genes
CCuG38008_ORF_1389	44	222	2.00E-61	227	MacB is an ATP-binding cassette (ABC) transporter that exports macrolides with 14- or 15- membered lactones
CCuG38008_ORF_1606	43	221	2.00E-56	244	MacB is an ATP-binding cassette (ABC) transporter that exports macrolides with 14- or 15- membered lactones
CCuG38008_ORF_1669	40	227	2.00E-54	233	vanRM is a vanR variant found in the vanM gene cluster
CCuG44481_ORF_44	45	225	1.00E-62	236	vanRM is a vanR variant found in the vanM gene cluster
CCuG44481_ORF_96	50	498	2.00E-172	490	LsaA is an ABC efflux pump expressed in Enterococcus faecalis
CCuG44481_ORF_246	48	233	1.00E-70	229	vanRF is a vanR variant found in the vanF gene cluster
CCuG44481_ORF_661	46	418	2.00E-121	441	Mycobacterium tuberculosis murA confers intrinsic resistance to fosfomycin
CCuG44481_ORF_702	69	396	0	395	Sequence variants of Streptomyces cinnamoneus elongation factor Tu that confer resistance to elfamycin antibiotics
CCuG44481_ORF_911	40	583	7.00E-146	578	AdeC is the outer membrane factor of the AdeABC multidrug efflux complex
CCuG44481_ORF_915	45	648	0	772	PBP1a is a penicillin-binding protein found in Streptococcus pneumoniae
CCuG44481_ORF_1148	41	233	1.00E-53	233	vanRE is a vanR variant found in the vanE gene cluster
CCuG44481_ORF_1338	40	222	7.00E-47	224	MacB is an ATP-binding cassette (ABC) transporter that exports macrolides with 14- or 15- membered lactones
CCuG44481_ORF_1401	44	222	1.00E-61	227	MacB is an ATP-binding cassette (ABC) transporter that exports macrolides with 14- or 15- membered lactones
CCuG44481_ORF_1700	58	67	8.00E-24	84	VanU is a transcriptional activator of the vanG operon of vancomycin resistance genes
CCuG44481_ORF_1782	99	458	0	458	TetL is a tetracycline efflux protein found in many species of Gram-negative and Gram-positive bacteria
CCuG44481_ORF_1931	90	641	0	646	TetM is a ribosomal protection protein that confers tetracycline resistance
CCuG44481_ORF_1937	99	215	4.00E-156	215	cat is used to describe many variants of the chloramphenicol acetyltransferase gene in a range of organisms
CCuG44481_ORF_1974	51	306	8.00E-109	326	bcrA is an ABC transporter found in Bacillus licheniformis that confers bacitracin resistance
CCuG45735_ORF_51	45	225	1.00E-62	236	vanRM is a vanR variant found in the vanM gene cluster
CCuG45735_ORF_70	55	95	7.00E-31	99	LsaA is an ABC efflux pump expressed in Enterococcus faecalis
CCuG45735_ORF_71	49	391	5.00E-131	387	LsaA is an ABC efflux pump expressed in Enterococcus faecalis
CCuG45735_ORF_227	48	233	1.00E-70	229	vanRF is a vanR variant found in the vanF gene cluster
CCuG45735_ORF_644	46	418	1.00E-120	441	Mycobacterium tuberculosis murA confers intrinsic resistance to fosfomycin
CCuG45735_ORF_685	69	396	0	395	Sequence variants of Streptomyces cinnamoneus elongation factor Tu that confer resistance to elfamycin antibiotics
CCuG45735_ORF_918	40	583	9.00E-146	578	AdeC is the outer membrane factor of the AdeABC multidrug efflux complex
CCuG45735_ORF_922	45	648	0	772	PBP1a is a penicillin-binding protein found in Streptococcus pneumoniae
CCuG45735_ORF_1183	41	233	6.00E-54	233	vanRE is a vanR variant found in the vanE gene cluster
CCuG45735_ORF_1365	40	219	1.00E-46	224	MacB is an ATP-binding cassette (ABC) transporter that exports macrolides with 14- or 15- membered lactones
CCuG45735_ORF_1376	45	302	2.00E-80	302	bcrA is an ABC transporter found in Bacillus licheniformis that confers bacitracin resistance
CCuG45735_ORF_1461	44	222	4.00E-62	227	MacB is an ATP-binding cassette (ABC) transporter that exports macrolides with 14- or 15- membered lactones
CCuG45735_ORF_1681	43	219	4.00E-53	233	MacB is an ATP-binding cassette (ABC) transporter that exports macrolides with 14- or 15- membered lactones
CCuG45735_ORF_1865	58	67	8.00E-24	84	VanU is a transcriptional activator of the vanG operon of vancomycin resistance genes
CCuG47171_ORF_51	45	225	1.00E-62	236	vanRM is a vanR variant found in the vanM gene cluster
CCuG47171_ORF_73	49	498	2.00E-171	493	LsaA is an ABC efflux pump expressed in Enterococcus faecalis
CCuG47171_ORF_217	48	233	1.00E-70	229	vanRF is a vanR variant found in the vanF gene cluster
CCuG47171_ORF_594	46	418	2.00E-121	441	Mycobacterium tuberculosis murA confers intrinsic resistance to fosfomycin
CCuG47171_ORF_634	69	396	0	395	Sequence variants of Streptomyces cinnamoneus elongation factor Tu that confer resistance to elfamycin antibiotics
CCuG47171_ORF_889	40	583	2.00E-145	578	AdeC is the outer membrane factor of the AdeABC multidrug efflux complex
CCuG47171_ORF_893	45	648	0	772	PBP1a is a penicillin-binding protein found in Streptococcus pneumoniae
CCuG47171_ORF_1141	41	233	3.00E-54	233	vanRE is a vanR variant found in the vanE gene cluster
CCuG47171_ORF_1371	40	222	7.00E-47	224	MacB is an ATP-binding cassette (ABC) transporter that exports macrolides with 14- or 15- membered lactones
CCuG47171_ORF_1434	44	222	4.00E-62	227	MacB is an ATP-binding cassette (ABC) transporter that exports macrolides with 14- or 15- membered lactones
CCuG47171_ORF_1663	44	204	3.00E-51	219	MacB is an ATP-binding cassette (ABC) transporter that exports macrolides with 14- or 15- membered lactones
CCuG47171_ORF_1918	58	67	8.00E-24	84	VanU is a transcriptional activator of the vanG operon of vancomycin resistance genes
CCuG47171_ORF_2091	41	231	2.00E-53	229	MacB is an ATP-binding cassette (ABC) transporter that exports macrolides with 14- or 15- membered lactones
CCuG47825_ORF_286	41	136	4.00E-26	154	MacB is an ATP-binding cassette (ABC) transporter that exports macrolides with 14- or 15- membered lactones
CCuG47825_ORF_287	49	81	1.00E-20	102	MacB is an ATP-binding cassette (ABC) transporter that exports macrolides with 14- or 15- membered lactones
CCuG47825_ORF_505	43	90	4.00E-21	94	MacB is an ATP-binding cassette (ABC) transporter that exports macrolides with 14- or 15- membered lactones
CCuG47825_ORF_506	47	122	3.00E-30	128	MacB is an ATP-binding cassette (ABC) transporter that exports macrolides with 14- or 15- membered lactones
CCuG47825_ORF_570	40	222	7.00E-47	224	MacB is an ATP-binding cassette (ABC) transporter that exports macrolides with 14- or 15- membered lactones
CCuG47825_ORF_773	46	418	1.00E-122	438	Mycobacterium tuberculosis murA confers intrinsic resistance to fosfomycin
CCuG47825_ORF_817	69	396	0	395	Sequence variants of Streptomyces cinnamoneus elongation factor Tu that confer resistance to elfamycin antibiotics
CCuG47825_ORF_1076	41	493	7.00E-129	494	AdeC is the outer membrane factor of the AdeABC multidrug efflux complex
CCuG47825_ORF_1080	45	648	0	772	PBP1a is a penicillin-binding protein found in Streptococcus pneumoniae
CCuG47825_ORF_1351	45	225	1.00E-62	248	vanRM is a vanR variant found in the vanM gene cluster
CCuG47825_ORF_1369	50	498	7.00E-174	493	LsaA is an ABC efflux pump expressed in Enterococcus faecalis
CCuG47825_ORF_1449	41	233	3.00E-54	233	vanRE is a vanR variant found in the vanE gene cluster
CCuG47825_ORF_1451	44	77	0.00E+00	93	vanSL is a vanS variant found in the vanL gene cluster
CCuG47825_ORF_1688	40	164	3.00E-32	172	A type III ABC transporter, identified on the novobiocin biosynthetic gene cluster
CCuG47825_ORF_1696	48	233	1.00E-70	229	vanRF is a vanR variant found in the vanF gene cluster
CCuG47825_ORF_1830	58	67	8.00E-24	84	VanU is a transcriptional activator of the vanG operon of vancomycin resistance genes
CCuG47826_ORF_51	45	225	1.00E-62	236	vanRM is a vanR variant found in the vanM gene cluster
CCuG47826_ORF_68	50	498	7.00E-174	493	LsaA is an ABC efflux pump expressed in Enterococcus faecalis
CCuG47826_ORF_234	48	233	1.00E-70	229	vanRF is a vanR variant found in the vanF gene cluster
CCuG47826_ORF_242	40	164	3.00E-32	172	A type III ABC transporter, identified on the novobiocin biosynthetic gene cluster
CCuG47826_ORF_556	46	418	8.00E-123	440	Mycobacterium tuberculosis murA confers intrinsic resistance to fosfomycin
CCuG47826_ORF_596	69	396	0	395	Sequence variants of Streptomyces cinnamoneus elongation factor Tu that confer resistance to elfamycin antibiotics
CCuG47826_ORF_907	41	569	3.00E-147	578	AdeC is the outer membrane factor of the AdeABC multidrug efflux complex
CCuG47826_ORF_911	45	648	0	772	PBP1a is a penicillin-binding protein found in Streptococcus pneumoniae
CCuG47826_ORF_1164	41	233	3.00E-54	233	vanRE is a vanR variant found in the vanE gene cluster
CCuG47826_ORF_1452	40	222	7.00E-47	224	MacB is an ATP-binding cassette (ABC) transporter that exports macrolides with 14- or 15- membered lactones
CCuG47826_ORF_1514	43	222	3.00E-60	227	MacB is an ATP-binding cassette (ABC) transporter that exports macrolides with 14- or 15- membered lactones
CCuG47826_ORF_1724	43	219	5.00E-53	233	MacB is an ATP-binding cassette (ABC) transporter that exports macrolides with 14- or 15- membered lactones
CCuG47826_ORF_1842	58	67	8.00E-24	84	VanU is a transcriptional activator of the vanG operon of vancomycin resistance genes
CECT5713_ORF_44	45	225	1.00E-62	236	vanRM is a vanR variant found in the vanM gene cluster
CECT5713_ORF_64	50	500	6.00E-172	493	LsaA is an ABC efflux pump expressed in Enterococcus faecalis
CECT5713_ORF_249	48	231	4.00E-70	229	vanRF is a vanR variant found in the vanF gene cluster
CECT5713_ORF_699	46	418	2.00E-121	441	Mycobacterium tuberculosis murA confers intrinsic resistance to fosfomycin
CECT5713_ORF_742	69	396	0	395	Sequence variants of Streptomyces cinnamoneus elongation factor Tu that confer resistance to elfamycin antibiotics
CECT5713_ORF_1052	41	127	3.00E-28	136	AdeC is the outer membrane factor of the AdeABC multidrug efflux complex
CECT5713_ORF_1053	45	350	3.00E-102	412	AdeC is the outer membrane factor of the AdeABC multidrug efflux complex

CECT5713_ORF_1057	45	648	0	772	PBP1a is a penicillin-binding protein found in Streptococcus pneumoniae
CECT5713_ORF_1328	41	233	3.00E-54	233	vanRE is a vanR variant found in the vanE gene cluster
CECT5713_ORF_1549	40	222	7.00E-47	224	MacB is an ATP-binding cassette (ABC) transporter that exports macrolides with 14- or 15- membered lactones
CECT5713_ORF_1563	45	305	1.00E-79	302	bcrA is an ABC transporter found in Bacillus licheniformis that confers bacitracin resistance
CECT5713_ORF_1647	44	222	2.00E-61	227	MacB is an ATP-binding cassette (ABC) transporter that exports macrolides with 14- or 15- membered lactones
CECT5713_ORF_1903	43	219	5.00E-53	233	MacB is an ATP-binding cassette (ABC) transporter that exports macrolides with 14- or 15- membered lactones
cp400_ORF_61	45	225	1.00E-62	236	vanRM is a vanR variant found in the vanM gene cluster
cp400_ORF_244	48	233	1.00E-70	229	vanRF is a vanR variant found in the vanF gene cluster
cp400_ORF_747	46	418	2.00E-121	441	Mycobacterium tuberculosis murA confers intrinsic resistance to fosfomycin
cp400_ORF_789	69	396	0	395	Sequence variants of Streptomyces cinnamoneus elongation factor Tu that confer resistance to elfamycin antibiotics
cp400_ORF_799	40	583	2.00E-147	578	AdeC is the outer membrane factor of the AdeABC multidrug efflux complex
cp400_ORF_803	45	648	0	772	PBP1a is a penicillin-binding protein found in Streptococcus pneumoniae
cp400_ORF_1294	41	233	3.00E-54	233	vanRE is a vanR variant found in the vanE gene cluster
cp400_ORF_1551	40	222	1.00E-45	224	MacB is an ATP-binding cassette (ABC) transporter that exports macrolides with 14- or 15- membered lactones
cp400_ORF_1615	44	222	4.00E-62	227	MacB is an ATP-binding cassette (ABC) transporter that exports macrolides with 14- or 15- membered lactones
cp400_ORF_2079	60	60	2.00E-20	71	VanU is a transcriptional activator of the vanG operon of vancomycin resistance genes
cp400_ORF_2106	50	498	8.00E-168	493	LsaA is an ABC efflux pump expressed in Enterococcus faecalis
DSM18933_ORF_149	42	107	3.00E-23	110	EmrE is a small multidrug transporter that functions as a homodimer
DSM18933_ORF_286	47	226	1.00E-67	233	MacB is an ATP-binding cassette (ABC) transporter that exports macrolides with 14- or 15- membered lactones
DSM18933_ORF_502	44	225	1.00E-59	236	vanRF is a vanR variant found in the vanF gene cluster
DSM18933_ORF_616	49	228	3.00E-70	229	vanRF is a vanR variant found in the vanF gene cluster
DSM18933_ORF_1243	44	417	7.00E-115	422	Mycobacterium tuberculosis murA confers intrinsic resistance to fosfomycin
DSM18933_ORF_1422	46	645	0	766	PBP1a is a penicillin-binding protein found in Streptococcus pneumoniae
DSM18933_ORF_1449	68	396	0	395	Sequence variants of Streptomyces cinnamoneus elongation factor Tu that confer resistance to elfamycin antibiotics
DSM20492_ORF_47	45	225	1.00E-62	236	vanRM is a vanR variant found in the vanM gene cluster
DSM20492_ORF_69	50	500	5.00E-172	493	LsaA is an ABC efflux pump expressed in Enterococcus faecalis
DSM20492_ORF_218	48	233	1.00E-70	229	vanRF is a vanR variant found in the vanF gene cluster
DSM20492_ORF_577	46	418	3.00E-121	440	Mycobacterium tuberculosis murA confers intrinsic resistance to fosfomycin
DSM20492_ORF_617	69	396	0	395	Sequence variants of Streptomyces cinnamoneus elongation factor Tu that confer resistance to elfamycin antibiotics
DSM20492_ORF_862	40	583	9.00E-148	578	AdeC is the outer membrane factor of the AdeABC multidrug efflux complex
DSM20492_ORF_866	45	648	0	772	PBP1a is a penicillin-binding protein found in Streptococcus pneumoniae
DSM20492_ORF_1098	41	233	3.00E-54	233	vanRE is a vanR variant found in the vanE gene cluster
DSM20492_ORF_1287	40	222	7.00E-47	224	MacB is an ATP-binding cassette (ABC) transporter that exports macrolides with 14- or 15- membered lactones
DSM20492_ORF_1350	44	222	4.00E-62	227	MacB is an ATP-binding cassette (ABC) transporter that exports macrolides with 14- or 15- membered lactones
DSM20492_ORF_1554	43	221	1.00E-56	233	MacB is an ATP-binding cassette (ABC) transporter that exports macrolides with 14- or 15- membered lactones
DSM20554_ORF_46	45	225	1.00E-62	236	vanRM is a vanR variant found in the vanM gene cluster
DSM20554_ORF_68	50	500	5.00E-172	493	LsaA is an ABC efflux pump expressed in Enterococcus faecalis
DSM20554_ORF_208	48	233	1.00E-70	229	vanRF is a vanR variant found in the vanF gene cluster
DSM20554_ORF_565	46	418	3.00E-121	440	Mycobacterium tuberculosis murA confers intrinsic resistance to fosfomycin
DSM20554_ORF_681	69	396	0	395	Sequence variants of Streptomyces cinnamoneus elongation factor Tu that confer resistance to elfamycin antibiotics
DSM20554_ORF_910	40	583	9.00E-148	578	AdeC is the outer membrane factor of the AdeABC multidrug efflux complex
DSM20554_ORF_914	45	648	0	772	PBP1a is a penicillin-binding protein found in Streptococcus pneumoniae
DSM20554_ORF_1106	41	233	3.00E-54	233	vanRE is a vanR variant found in the vanE gene cluster
DSM20554_ORF_1298	40	222	7.00E-47	224	MacB is an ATP-binding cassette (ABC) transporter that exports macrolides with 14- or 15- membered lactones
DSM20554_ORF_1361	44	222	4.00E-62	227	MacB is an ATP-binding cassette (ABC) transporter that exports macrolides with 14- or 15- membered lactones
DSM20554_ORF_1581	43	221	1.00E-56	233	MacB is an ATP-binding cassette (ABC) transporter that exports macrolides with 14- or 15- membered lactones
DSM20555_ORF_55	45	225	1.00E-62	236	vanRM is a vanR variant found in the vanM gene cluster
DSM20555_ORF_72	50	498	3.00E-172	493	LsaA is an ABC efflux pump expressed in Enterococcus faecalis
DSM20555_ORF_212	48	233	1.00E-70	229	vanRF is a vanR variant found in the vanF gene cluster
DSM20555_ORF_554	46	418	2.00E-121	441	Mycobacterium tuberculosis murA confers intrinsic resistance to fosfomycin
DSM20555_ORF_594	69	396	0	395	Sequence variants of Streptomyces cinnamoneus elongation factor Tu that confer resistance to elfamycin antibiotics
DSM20555_ORF_815	41	573	9.00E-147	578	AdeC is the outer membrane factor of the AdeABC multidrug efflux complex
DSM20555_ORF_819	45	648	0	772	PBP1a is a penicillin-binding protein found in Streptococcus pneumoniae
DSM20555_ORF_1069	41	233	4.00E-54	233	vanRE is a vanR variant found in the vanE gene cluster
DSM20555_ORF_1257	40	222	2.00E-46	224	MacB is an ATP-binding cassette (ABC) transporter that exports macrolides with 14- or 15- membered lactones
DSM20555_ORF_1319	44	222	2.00E-61	227	MacB is an ATP-binding cassette (ABC) transporter that exports macrolides with 14- or 15- membered lactones
DSM20555_ORF_1535	43	221	1.00E-56	233	MacB is an ATP-binding cassette (ABC) transporter that exports macrolides with 14- or 15- membered lactones
GJ24_ORF_21	50	498	2.00E-171	493	LsaA is an ABC efflux pump expressed in Enterococcus faecalis
GJ24_ORF_180	48	233	1.00E-70	229	vanRF is a vanR variant found in the vanF gene cluster
GJ24_ORF_620	46	418	1.00E-120	441	Mycobacterium tuberculosis murA confers intrinsic resistance to fosfomycin
GJ24_ORF_661	69	396	0	395	Sequence variants of Streptomyces cinnamoneus elongation factor Tu that confer resistance to elfamycin antibiotics
GJ24_ORF_870	40	583	6.00E-146	578	AdeC is the outer membrane factor of the AdeABC multidrug efflux complex
GJ24_ORF_874	45	648	0	772	PBP1a is a penicillin-binding protein found in Streptococcus pneumoniae
GJ24_ORF_1110	41	233	3.00E-54	233	vanRE is a vanR variant found in the vanE gene cluster
GJ24_ORF_1344	40	222	2.00E-46	224	MacB is an ATP-binding cassette (ABC) transporter that exports macrolides with 14- or 15- membered lactones
GJ24_ORF_1407	44	222	1.00E-61	227	MacB is an ATP-binding cassette (ABC) transporter that exports macrolides with 14- or 15- membered lactones
GJ24_ORF_1736	45	225	1.00E-62	236	vanRM is a vanR variant found in the vanM gene cluster
gul1_ORF_54	45	225	1.00E-62	236	vanRM is a vanR variant found in the vanM gene cluster
gul1_ORF_71	50	498	3.00E-172	493	LsaA is an ABC efflux pump expressed in Enterococcus faecalis
gul1_ORF_215	48	233	1.00E-70	229	vanRF is a vanR variant found in the vanF gene cluster
gul1_ORF_519	46	418	2.00E-121	441	Mycobacterium tuberculosis murA confers intrinsic resistance to fosfomycin
gul1_ORF_560	69	396	0	395	Sequence variants of Streptomyces cinnamoneus elongation factor Tu that confer resistance to elfamycin antibiotics
gul1_ORF_783	41	573	9.00E-147	578	AdeC is the outer membrane factor of the AdeABC multidrug efflux complex
gul1_ORF_787	45	648	0	772	PBP1a is a penicillin-binding protein found in Streptococcus pneumoniae
gul1_ORF_1039	41	233	4.00E-54	233	vanRE is a vanR variant found in the vanE gene cluster
gul1_ORF_1226	40	222	2.00E-46	224	MacB is an ATP-binding cassette (ABC) transporter that exports macrolides with 14- or 15- membered lactones
gul1_ORF_1288	44	222	2.00E-61	227	MacB is an ATP-binding cassette (ABC) transporter that exports macrolides with 14- or 15- membered lactones
gul1_ORF_1505	43	221	1.00E-56	233	MacB is an ATP-binding cassette (ABC) transporter that exports macrolides with 14- or 15- membered lactones
gul2_ORF_55	45	225	1.00E-62	236	vanRM is a vanR variant found in the vanM gene cluster
gul2_ORF_72	50	498	3.00E-172	493	LsaA is an ABC efflux pump expressed in Enterococcus faecalis
gul2_ORF_217	48	233	1.00E-70	229	vanRF is a vanR variant found in the vanF gene cluster
gul2_ORF_559	46	418	2.00E-121	441	Mycobacterium tuberculosis murA confers intrinsic resistance to fosfomycin
gul2_ORF_600	69	396	0	395	Sequence variants of Streptomyces cinnamoneus elongation factor Tu that confer resistance to elfamycin antibiotics
gul2_ORF_825	41	573	9.00E-147	578	AdeC is the outer membrane factor of the AdeABC multidrug efflux complex
gul2_ORF_829	45	648	0	772	PBP1a is a penicillin-binding protein found in Streptococcus pneumoniae
gul2_ORF_1081	41	233	4.00E-54	233	vanRE is a vanR variant found in the vanE gene cluster
gul2_ORF_1268	40	222	2.00E-46	224	MacB is an ATP-binding cassette (ABC) transporter that exports macrolides with 14- or 15- membered lactones
gul2_ORF_1330	44	222	2.00E-61	227	MacB is an ATP-binding cassette (ABC) transporter that exports macrolides with 14- or 15- membered lactones
gul2_ORF_1547	43	221	1.00E-56	233	MacB is an ATP-binding cassette (ABC) transporter that exports macrolides with 14- or 15- membered lactones
JCM1040_ORF_49	45	225	1.00E-62	236	vanRM is a vanR variant found in the vanM gene cluster
JCM1040_ORF_70	50	498	4.00E-171	493	LsaA is an ABC efflux pump expressed in Enterococcus faecalis
JCM1040_ORF_215	48	233	1.00E-70	229	vanRF is a vanR variant found in the vanF gene cluster
JCM1040_ORF_599	46	418	8.00E-123	440	Mycobacterium tuberculosis murA confers intrinsic resistance to fosfomycin
JCM1040_ORF_639	69	396	0	395	Sequence variants of Streptomyces cinnamoneus elongation factor Tu that confer resistance to elfamycin antibiotics
JCM1040_ORF_876	41	569	4.00E-147	578	AdeC is the outer membrane factor of the AdeABC multidrug efflux complex
JCM1040_ORF_880	45	648	0	772	PBP1a is a penicillin-binding protein found in Streptococcus pneumoniae
JCM1040_ORF_1123	41	233	3.00E-54	233	vanRE is a vanR variant found in the vanE gene cluster
JCM1040_ORF_1300	40	222	7.00E-47	224	MacB is an ATP-binding cassette (ABC) transporter that exports macrolides with 14- or 15- membered lactones
JCM1040_ORF_1362	44	222	4.00E-62	227	MacB is an ATP-binding cassette (ABC) transporter that exports macrolides with 14- or 15- membered lactones
JCM1040_ORF_1589	43	219	2.00E-53	233	MacB is an ATP-binding cassette (ABC) transporter that exports macrolides with 14- or 15- membered lactones
JCM1040_ORF_1656	40	227	1.00E-54	233	vanRM is a vanR variant found in the vanM gene cluster
JCM1042_ORF_53	45	225	1.00E-62	236	vanRM is a vanR variant found in the vanM gene cluster
JCM1042_ORF_73	49	500	1.00E-171	493	LsaA is an ABC efflux pump expressed in Enterococcus faecalis
JCM1042_ORF_218	48	233	1.00E-70	229	vanRF is a vanR variant found in the vanF gene cluster
JCM1042_ORF_549	46	418	2.00E-121	441	Mycobacterium tuberculosis murA confers intrinsic resistance to fosfomycin
JCM1042_ORF_590	69	396	0	395	Sequence variants of Streptomyces cinnamoneus elongation factor Tu that confer resistance to elfamycin antibiotics
JCM1042_ORF_819	40	583	4.00E-146	578	AdeC is the outer membrane factor of the AdeABC multidrug efflux complex
JCM1042_ORF_824	45	648	0	772	PBP1a is a penicillin-binding protein found in Streptococcus pneumoniae
JCM1042_ORF_1071	41	233	3.00E-54	233	vanRE is a vanR variant found in the vanE gene cluster

JCM1042_ORF_1252	40	222	7.00E-47	224	MacB is an ATP-binding cassette (ABC) transporter that exports macrolides with 14- or 15- membered lactones
JCM1042_ORF_1315	44	222	1.00E-61	227	MacB is an ATP-binding cassette (ABC) transporter that exports macrolides with 14- or 15- membered lactones
JCM1042_ORF_1545	43	221	2.00E-56	244	MacB is an ATP-binding cassette (ABC) transporter that exports macrolides with 14- or 15- membered lactones
JCM1044_ORF_54	45	225	1.00E-62	236	vanRM is a vanR variant found in the vanM gene cluster
JCM1044_ORF_71	49	500	1.00E-171	493	LsaA is an ABC efflux pump expressed in Enterococcus faecalis
JCM1044_ORF_216	48	233	1.00E-70	229	vanRF is a vanR variant found in the vanF gene cluster
JCM1044_ORF_504	46	418	2.00E-121	441	Mycobacterium tuberculosis murA confers intrinsic resistance to fosfomycin
JCM1044_ORF_544	69	396	0	395	Sequence variants of Streptomyces cinnamoneus elongation factor Tu that confer resistance to elfamycin antibiotics
JCM1044_ORF_778	40	583	4.00E-146	578	AdeC is the outer membrane factor of the AdeABC multidrug efflux complex
JCM1044_ORF_783	45	648	0	772	PBP1a is a penicillin-binding protein found in Streptococcus pneumoniae
JCM1044_ORF_1031	41	233	3.00E-54	233	vanRE is a vanR variant found in the vanE gene cluster
JCM1044_ORF_1210	40	222	7.00E-47	224	MacB is an ATP-binding cassette (ABC) transporter that exports macrolides with 14- or 15- membered lactones
JCM1044_ORF_1273	44	222	1.00E-61	227	MacB is an ATP-binding cassette (ABC) transporter that exports macrolides with 14- or 15- membered lactones
JCM1044_ORF_1498	43	221	2.00E-56	244	MacB is an ATP-binding cassette (ABC) transporter that exports macrolides with 14- or 15- membered lactones
JCM1045_ORF_56	45	225	1.00E-62	236	vanRM is a vanR variant found in the vanM gene cluster
JCM1045_ORF_74	50	498	3.00E-172	493	LsaA is an ABC efflux pump expressed in Enterococcus faecalis
JCM1045_ORF_215	48	233	1.00E-70	229	vanRF is a vanR variant found in the vanF gene cluster
JCM1045_ORF_544	46	418	4.00E-121	440	Mycobacterium tuberculosis murA confers intrinsic resistance to fosfomycin
JCM1045_ORF_649	69	396	0	395	Sequence variants of Streptomyces cinnamoneus elongation factor Tu that confer resistance to elfamycin antibiotics
JCM1045_ORF_886	41	583	2.00E-148	578	AdeC is the outer membrane factor of the AdeABC multidrug efflux complex
JCM1045_ORF_890	45	648	0	772	PBP1a is a penicillin-binding protein found in Streptococcus pneumoniae
JCM1045_ORF_1145	41	233	4.00E-54	233	vanRE is a vanR variant found in the vanE gene cluster
JCM1045_ORF_1343	40	222	5.00E-47	224	MacB is an ATP-binding cassette (ABC) transporter that exports macrolides with 14- or 15- membered lactones
JCM1045_ORF_1411	44	222	1.00E-61	227	MacB is an ATP-binding cassette (ABC) transporter that exports macrolides with 14- or 15- membered lactones
JCM1046_ORF_54	45	225	1.00E-62	236	vanRM is a vanR variant found in the vanM gene cluster
JCM1046_ORF_75	50	498	8.00E-168	493	LsaA is an ABC efflux pump expressed in Enterococcus faecalis
JCM1046_ORF_220	48	233	1.00E-70	229	vanRF is a vanR variant found in the vanF gene cluster
JCM1046_ORF_569	46	418	2.00E-121	441	Mycobacterium tuberculosis murA confers intrinsic resistance to fosfomycin
JCM1046_ORF_609	69	396	0	395	Sequence variants of Streptomyces cinnamoneus elongation factor Tu that confer resistance to elfamycin antibiotics
JCM1046_ORF_874	40	583	2.00E-147	578	AdeC is the outer membrane factor of the AdeABC multidrug efflux complex
JCM1046_ORF_878	45	648	0	772	PBP1a is a penicillin-binding protein found in Streptococcus pneumoniae
JCM1046_ORF_1099	41	233	3.00E-54	233	vanRE is a vanR variant found in the vanE gene cluster
JCM1046_ORF_1251	62	61	2.00E-22	73	VanU is a transcriptional activator of the vanG operon of vancomycin resistance genes
JCM1046_ORF_1320	40	222	1.00E-45	224	MacB is an ATP-binding cassette (ABC) transporter that exports macrolides with 14- or 15- membered lactones
JCM1046_ORF_1382	44	222	1.00E-61	227	MacB is an ATP-binding cassette (ABC) transporter that exports macrolides with 14- or 15- membered lactones
JCM1046_ORF_1829	90	639	0	644	TetM is a ribosomal protection protein that confers tetracycline resistance
JCM1047_ORF_54	45	225	1.00E-62	236	vanRM is a vanR variant found in the vanM gene cluster
JCM1047_ORF_91	49	496	8.00E-172	492	LsaA is an ABC efflux pump expressed in Enterococcus faecalis
JCM1047_ORF_241	48	233	1.00E-70	229	vanRF is a vanR variant found in the vanF gene cluster
JCM1047_ORF_664	46	418	7.00E-122	441	Mycobacterium tuberculosis murA confers intrinsic resistance to fosfomycin
JCM1047_ORF_720	69	396	0	395	Sequence variants of Streptomyces cinnamoneus elongation factor Tu that confer resistance to elfamycin antibiotics
JCM1047_ORF_1061	40	579	2.00E-144	578	AdeC is the outer membrane factor of the AdeABC multidrug efflux complex
JCM1047_ORF_1065	45	648	0	766	PBP1a is a penicillin-binding protein found in Streptococcus pneumoniae
JCM1047_ORF_1301	41	233	1.00E-53	233	vanRE is a vanR variant found in the vanE gene cluster
JCM1047_ORF_1485	40	222	1.00E-46	224	MacB is an ATP-binding cassette (ABC) transporter that exports macrolides with 14- or 15- membered lactones
JCM1047_ORF_1547	44	222	1.00E-61	227	MacB is an ATP-binding cassette (ABC) transporter that exports macrolides with 14- or 15- membered lactones
JCM1047_ORF_2131	58	67	8.00E-24	84	VanU is a transcriptional activator of the vanG operon of vancomycin resistance genes
JCM1047_ORF_2228	90	641	0	646	TetM is a ribosomal protection protein that confers tetracycline resistance
JCM1230_ORF_72	45	225	1.00E-62	236	vanRM is a vanR variant found in the vanM gene cluster
JCM1230_ORF_93	50	498	4.00E-172	493	LsaA is an ABC efflux pump expressed in Enterococcus faecalis
JCM1230_ORF_258	48	233	1.00E-70	229	vanRF is a vanR variant found in the vanF gene cluster
JCM1230_ORF_548	46	418	2.00E-121	441	Mycobacterium tuberculosis murA confers intrinsic resistance to fosfomycin
JCM1230_ORF_588	69	396	0	395	Sequence variants of Streptomyces cinnamoneus elongation factor Tu that confer resistance to elfamycin antibiotics
JCM1230_ORF_798	40	583	7.00E-145	578	AdeC is the outer membrane factor of the AdeABC multidrug efflux complex
JCM1230_ORF_802	45	648	0	772	PBP1a is a penicillin-binding protein found in Streptococcus pneumoniae
JCM1230_ORF_1046	41	233	3.00E-54	233	vanRE is a vanR variant found in the vanE gene cluster
JCM1230_ORF_1288	40	222	1.00E-46	224	MacB is an ATP-binding cassette (ABC) transporter that exports macrolides with 14- or 15- membered lactones
JCM1230_ORF_1350	44	222	1.00E-61	227	MacB is an ATP-binding cassette (ABC) transporter that exports macrolides with 14- or 15- membered lactones
L21_ORF_50	45	225	1.00E-62	236	vanRM is a vanR variant found in the vanM gene cluster
L21_ORF_67	50	498	7.00E-174	493	LsaA is an ABC efflux pump expressed in Enterococcus faecalis
L21_ORF_222	48	233	1.00E-70	229	vanRF is a vanR variant found in the vanF gene cluster
L21_ORF_688	46	418	8.00E-123	440	Mycobacterium tuberculosis murA confers intrinsic resistance to fosfomycin
L21_ORF_728	69	396	0	395	Sequence variants of Streptomyces cinnamoneus elongation factor Tu that confer resistance to elfamycin antibiotics
L21_ORF_969	41	573	4.00E-147	578	AdeC is the outer membrane factor of the AdeABC multidrug efflux complex
L21_ORF_973	45	648	0	772	PBP1a is a penicillin-binding protein found in Streptococcus pneumoniae
L21_ORF_1225	41	233	3.00E-54	233	vanRE is a vanR variant found in the vanE gene cluster
L21_ORF_1405	40	222	7.00E-47	224	MacB is an ATP-binding cassette (ABC) transporter that exports macrolides with 14- or 15- membered lactones
L21_ORF_1467	44	222	2.00E-61	227	MacB is an ATP-binding cassette (ABC) transporter that exports macrolides with 14- or 15- membered lactones
L21_ORF_1675	43	219	5.00E-53	233	MacB is an ATP-binding cassette (ABC) transporter that exports macrolides with 14- or 15- membered lactones
L21_ORF_1986	58	67	8.00E-24	84	VanU is a transcriptional activator of the vanG operon of vancomycin resistance genes
LMG14476_ORF_69	45	225	1.00E-62	236	vanRM is a vanR variant found in the vanM gene cluster
LMG14476_ORF_90	50	498	8.00E-173	493	LsaA is an ABC efflux pump expressed in Enterococcus faecalis
LMG14476_ORF_231	48	231	2.00E-70	229	vanRF is a vanR variant found in the vanF gene cluster
LMG14476_ORF_268	40	263	1.00E-59	327	vanHA, also known as vanH, is a vanH variant in the vanA gene cluster
LMG14476_ORF_549	46	418	2.00E-121	441	Mycobacterium tuberculosis murA confers intrinsic resistance to fosfomycin
LMG14476_ORF_589	69	396	0	395	Sequence variants of Streptomyces cinnamoneus elongation factor Tu that confer resistance to elfamycin antibiotics
LMG14476_ORF_968	40	583	7.00E-145	578	AdeC is the outer membrane factor of the AdeABC multidrug efflux complex
LMG14476_ORF_972	45	648	0	772	PBP1a is a penicillin-binding protein found in Streptococcus pneumoniae
LMG14476_ORF_1201	41	233	3.00E-54	233	vanRE is a vanR variant found in the vanE gene cluster
LMG14476_ORF_1347	60	61	3.00E-23	68	VanU is a transcriptional activator of the vanG operon of vancomycin resistance genes
LMG14476_ORF_1417	40	222	6.00E-47	224	MacB is an ATP-binding cassette (ABC) transporter that exports macrolides with 14- or 15- membered lactones
LMG14476_ORF_1480	44	222	2.00E-61	227	MacB is an ATP-binding cassette (ABC) transporter that exports macrolides with 14- or 15- membered lactones
LMG14476_ORF_1711	43	221	4.00E-56	244	MacB is an ATP-binding cassette (ABC) transporter that exports macrolides with 14- or 15- membered lactones
LMG14477_ORF_52	45	225	1.00E-62	236	vanRM is a vanR variant found in the vanM gene cluster
LMG14477_ORF_73	50	498	8.00E-173	493	LsaA is an ABC efflux pump expressed in Enterococcus faecalis
LMG14477_ORF_211	48	231	2.00E-70	229	vanRF is a vanR variant found in the vanF gene cluster
LMG14477_ORF_248	40	263	1.00E-59	327	vanHA, also known as vanH, is a vanH variant in the vanA gene cluster
LMG14477_ORF_543	46	418	2.00E-121	441	Mycobacterium tuberculosis murA confers intrinsic resistance to fosfomycin
LMG14477_ORF_583	69	396	0	395	Sequence variants of Streptomyces cinnamoneus elongation factor Tu that confer resistance to elfamycin antibiotics
LMG14477_ORF_952	40	583	7.00E-145	578	AdeC is the outer membrane factor of the AdeABC multidrug efflux complex
LMG14477_ORF_956	45	648	0	772	PBP1a is a penicillin-binding protein found in Streptococcus pneumoniae
LMG14477_ORF_1185	41	233	3.00E-54	233	vanRE is a vanR variant found in the vanE gene cluster
LMG14477_ORF_1330	60	61	3.00E-23	68	VanU is a transcriptional activator of the vanG operon of vancomycin resistance genes
LMG14477_ORF_1401	40	222	6.00E-47	224	MacB is an ATP-binding cassette (ABC) transporter that exports macrolides with 14- or 15- membered lactones
LMG14477_ORF_1464	44	222	2.00E-61	227	MacB is an ATP-binding cassette (ABC) transporter that exports macrolides with 14- or 15- membered lactones
LMG14477_ORF_1697	43	221	4.00E-56	244	MacB is an ATP-binding cassette (ABC) transporter that exports macrolides with 14- or 15- membered lactones
NCIMB702343_ORF_53	45	225	1.00E-62	236	vanRM is a vanR variant found in the vanM gene cluster
NCIMB702343_ORF_79	48	223	3.00E-63	226	LsaA is an ABC efflux pump expressed in Enterococcus faecalis
NCIMB702343_ORF_80	52	183	4.00E-62	183	LsaA is an ABC efflux pump expressed in Enterococcus faecalis
NCIMB702343_ORF_236	48	233	1.00E-70	229	vanRF is a vanR variant found in the vanF gene cluster
NCIMB702343_ORF_578	69	396	0	395	Sequence variants of Streptomyces cinnamoneus elongation factor Tu that confer resistance to elfamycin antibiotics
NCIMB702343_ORF_619	46	418	2.00E-122	440	Mycobacterium tuberculosis murA confers intrinsic resistance to fosfomycin
NCIMB702343_ORF_850	40	583	2.00E-145	578	AdeC is the outer membrane factor of the AdeABC multidrug efflux complex
NCIMB702343_ORF_854	45	648	0	772	PBP1a is a penicillin-binding protein found in Streptococcus pneumoniae
NCIMB702343_ORF_1087	41	233	3.00E-54	233	vanRE is a vanR variant found in the vanE gene cluster
NCIMB702343_ORF_1243	62	62	7.00E-23	77	VanU is a transcriptional activator of the vanG operon of vancomycin resistance genes
NCIMB702343_ORF_1315	40	222	7.00E-47	224	MacB is an ATP-binding cassette (ABC) transporter that exports macrolides with 14- or 15- membered lactones
NCIMB702343_ORF_1381	44	222	4.00E-62	227	MacB is an ATP-binding cassette (ABC) transporter that exports macrolides with 14- or 15- membered lactones
NCIMB702343_ORF_1604	43	221	2.00E-56	244	MacB is an ATP-binding cassette (ABC) transporter that exports macrolides with 14- or 15- membered lactones

NCIMB702343_ORF_1779	58	67	8.00E-24	84	VanU is a transcriptional activator of the vanG operon of vancomycin resistance genes
NCIMB8816_ORF_55	45	225	1.00E-62	236	vanRM is a vanR variant found in the vanM gene cluster
NCIMB8816_ORF_76	49	500	1.00E-171	493	LsaA is an ABC efflux pump expressed in Enterococcus faecalis
NCIMB8816_ORF_221	48	233	1.00E-70	229	vanRF is a vanR variant found in the vanF gene cluster
NCIMB8816_ORF_507	46	418	2.00E-121	441	Mycobacterium tuberculosis murA confers intrinsic resistance to fosfomycin
NCIMB8816_ORF_547	69	396	0	395	Sequence variants of Streptomyces cinnamoneus elongation factor Tu that confer resistance to elfamycin antibiotics
NCIMB8816_ORF_782	40	583	4.00E-146	578	AdeC is the outer membrane factor of the AdeABC multidrug efflux complex
NCIMB8816_ORF_787	45	648	0	772	PBP1a is a penicillin-binding protein found in Streptococcus pneumoniae
NCIMB8816_ORF_1032	41	233	3.00E-54	233	vanRE is a vanR variant found in the vanE gene cluster
NCIMB8816_ORF_1253	40	222	7.00E-47	224	MacB is an ATP-binding cassette (ABC) transporter that exports macrolides with 14- or 15- membered lactones
NCIMB8816_ORF_1317	44	222	1.00E-61	227	MacB is an ATP-binding cassette (ABC) transporter that exports macrolides with 14- or 15- membered lactones
NCIMB8816_ORF_1545	43	221	2.00E-56	244	MacB is an ATP-binding cassette (ABC) transporter that exports macrolides with 14- or 15- membered lactones
NCIMB8816_ORF_1605	40	227	1.00E-54	233	vanRM is a vanR variant found in the vanM gene cluster
NCIMB8817_ORF_60	45	225	1.00E-62	236	vanRM is a vanR variant found in the vanM gene cluster
NCIMB8817_ORF_79	50	498	7.00E-171	493	LsaA is an ABC efflux pump expressed in Enterococcus faecalis
NCIMB8817_ORF_221	48	233	1.00E-70	229	vanRF is a vanR variant found in the vanF gene cluster
NCIMB8817_ORF_610	46	418	2.00E-121	441	Mycobacterium tuberculosis murA confers intrinsic resistance to fosfomycin
NCIMB8817_ORF_651	69	396	0	395	Sequence variants of Streptomyces cinnamoneus elongation factor Tu that confer resistance to elfamycin antibiotics
NCIMB8817_ORF_880	41	579	2.00E-148	578	AdeC is the outer membrane factor of the AdeABC multidrug efflux complex
NCIMB8817_ORF_884	45	648	0	772	PBP1a is a penicillin-binding protein found in Streptococcus pneumoniae
NCIMB8817_ORF_1133	41	233	3.00E-54	233	vanRE is a vanR variant found in the vanE gene cluster
NCIMB8817_ORF_1309	40	222	1.00E-46	224	MacB is an ATP-binding cassette (ABC) transporter that exports macrolides with 14- or 15- membered lactones
NCIMB8817_ORF_1371	44	222	9.00E-62	227	MacB is an ATP-binding cassette (ABC) transporter that exports macrolides with 14- or 15- membered lactones
NCIMB8818_ORF_51	45	225	1.00E-62	236	vanRM is a vanR variant found in the vanM gene cluster
NCIMB8818_ORF_71	50	498	2.00E-171	493	LsaA is an ABC efflux pump expressed in Enterococcus faecalis
NCIMB8818_ORF_226	49	231	3.00E-71	229	vanRF is a vanR variant found in the vanF gene cluster
NCIMB8818_ORF_452	43	219	5.00E-53	233	MacB is an ATP-binding cassette (ABC) transporter that exports macrolides with 14- or 15- membered lactones
NCIMB8818_ORF_740	46	418	2.00E-121	441	Mycobacterium tuberculosis murA confers intrinsic resistance to fosfomycin
NCIMB8818_ORF_780	69	396	0	395	Sequence variants of Streptomyces cinnamoneus elongation factor Tu that confer resistance to elfamycin antibiotics
NCIMB8818_ORF_1075	40	583	4.00E-146	578	AdeC is the outer membrane factor of the AdeABC multidrug efflux complex
NCIMB8818_ORF_1079	45	648	0	772	PBP1a is a penicillin-binding protein found in Streptococcus pneumoniae
NCIMB8818_ORF_1330	41	233	4.00E-54	233	vanRE is a vanR variant found in the vanE gene cluster
NCIMB8818_ORF_1484	62	61	6.00E-22	73	VanU is a transcriptional activator of the vanG operon of vancomycin resistance genes
NCIMB8818_ORF_1530	40	222	7.00E-47	224	MacB is an ATP-binding cassette (ABC) transporter that exports macrolides with 14- or 15- membered lactones
NCIMB8818_ORF_1541	45	305	1.00E-79	302	bcrA is an ABC transporter found in Bacillus licheniformis that confers bacitracin resistance
NCIMB8818_ORF_1650	44	222	1.00E-61	227	MacB is an ATP-binding cassette (ABC) transporter that exports macrolides with 14- or 15- membered lactones
NCIMB8818_ORF_1908	45	77	0.00E+00	76	A type III ABC transporter, identified on the novobiocin biosynthetic gene cluster
NIAS840_ORF_88	48	233	1.00E-70	229	vanRF is a vanR variant found in the vanF gene cluster
NIAS840_ORF_241	49	498	3.00E-171	493	LsaA is an ABC efflux pump expressed in Enterococcus faecalis
NIAS840_ORF_403	40	222	1.00E-46	224	MacB is an ATP-binding cassette (ABC) transporter that exports macrolides with 14- or 15- membered lactones
NIAS840_ORF_465	44	198	1.00E-52	207	MacB is an ATP-binding cassette (ABC) transporter that exports macrolides with 14- or 15- membered lactones
NIAS840_ORF_467	98	164	9.00E-119	164	lnuC is a transposon-mediated nucleotidyltransferase found in Streptococcus agalactiae
NIAS840_ORF_661	99	216	1.00E-158	216	vatH is a plasmid-mediated acetyltransferase found in Enterococcus faecium
NIAS840_ORF_662	99	525	0	525	vgaD is an efflux protein expressed in Enterococcus faecium that confers resistance to streptogramin A antibiotics
NIAS840_ORF_793	45	225	1.00E-62	236	vanRM is a vanR variant found in the vanM gene cluster
NIAS840_ORF_1075	46	418	2.00E-121	441	Mycobacterium tuberculosis murA confers intrinsic resistance to fosfomycin
NIAS840_ORF_1168	69	396	0	395	Sequence variants of Streptomyces cinnamoneus elongation factor Tu that confer resistance to elfamycin antibiotics
NIAS840_ORF_1378	41	583	1.00E-148	578	AdeC is the outer membrane factor of the AdeABC multidrug efflux complex
NIAS840_ORF_1382	45	648	0	772	PBP1a is a penicillin-binding protein found in Streptococcus pneumoniae
NIAS840_ORF_1588	41	233	3.00E-54	233	vanRE is a vanR variant found in the vanE gene cluster
Ren_ORF_41	45	225	1.00E-62	236	vanRM is a vanR variant found in the vanM gene cluster
Ren_ORF_63	50	498	4.00E-171	493	LsaA is an ABC efflux pump expressed in Enterococcus faecalis
Ren_ORF_230	48	233	2.00E-70	229	vanRF is a vanR variant found in the vanF gene cluster
Ren_ORF_651	46	418	8.00E-123	440	Mycobacterium tuberculosis murA confers intrinsic resistance to fosfomycin
Ren_ORF_691	69	396	0	395	Sequence variants of Streptomyces cinnamoneus elongation factor Tu that confer resistance to elfamycin antibiotics
Ren_ORF_929	41	569	4.00E-147	578	AdeC is the outer membrane factor of the AdeABC multidrug efflux complex
Ren_ORF_933	45	648	0	772	PBP1a is a penicillin-binding protein found in Streptococcus pneumoniae
Ren_ORF_1188	41	233	3.00E-54	233	vanRE is a vanR variant found in the vanE gene cluster
Ren_ORF_1386	40	222	3.00E-47	224	MacB is an ATP-binding cassette (ABC) transporter that exports macrolides with 14- or 15- membered lactones
Ren_ORF_1448	44	222	4.00E-62	227	MacB is an ATP-binding cassette (ABC) transporter that exports macrolides with 14- or 15- membered lactones
Ren_ORF_1680	49	81	7.00E-21	102	MacB is an ATP-binding cassette (ABC) transporter that exports macrolides with 14- or 15- membered lactones
Ren_ORF_1681	46	110	1.00E-26	131	MacB is an ATP-binding cassette (ABC) transporter that exports macrolides with 14- or 15- membered lactones
Ren_ORF_1761	58	67	8.00E-24	84	VanU is a transcriptional activator of the vanG operon of vancomycin resistance genes
Ren_ORF_1999	40	227	1.00E-54	233	vanRM is a vanR variant found in the vanM gene cluster
SMXD51_ORF_100	45	225	1.00E-62	236	vanRM is a vanR variant found in the vanM gene cluster
SMXD51_ORF_205	50	498	1.00E-171	493	LsaA is an ABC efflux pump expressed in Enterococcus faecalis
SMXD51_ORF_366	48	233	1.00E-70	229	vanRF is a vanR variant found in the vanF gene cluster
SMXD51_ORF_691	44	222	1.00E-61	227	MacB is an ATP-binding cassette (ABC) transporter that exports macrolides with 14- or 15- membered lactones
SMXD51_ORF_758	40	222	2.00E-46	224	MacB is an ATP-binding cassette (ABC) transporter that exports macrolides with 14- or 15- membered lactones
SMXD51_ORF_1013	46	418	2.00E-121	441	Mycobacterium tuberculosis murA confers intrinsic resistance to fosfomycin
SMXD51_ORF_1055	69	396	0	395	Sequence variants of Streptomyces cinnamoneus elongation factor Tu that confer resistance to elfamycin antibiotics
SMXD51_ORF_1276	40	583	6.00E-146	578	AdeC is the outer membrane factor of the AdeABC multidrug efflux complex
SMXD51_ORF_1280	45	648	0	772	PBP1a is a penicillin-binding protein found in Streptococcus pneumoniae
SMXD51_ORF_1513	41	233	3.00E-54	233	vanRE is a vanR variant found in the vanE gene cluster
SMXD51_ORF_1702	97	242	5.00E-173	258	ErmC is a methyltransferase that catalyzes the methylation of A2058 of the 23S ribosomal RNA in two steps
SMXD51_ORF_1720	89	639	0	644	TetM is a ribosomal protection protein that confers tetracycline resistance
SMXD51_ORF_1722	98	436	0	442	TetL is a tetracycline efflux protein found in many species of Gram-negative and Gram-positive bacteria
UCC118_ORF_41	45	225	1.00E-62	236	vanRM is a vanR variant found in the vanM gene cluster
UCC118_ORF_61	50	498	3.00E-171	493	LsaA is an ABC efflux pump expressed in Enterococcus faecalis
UCC118_ORF_240	48	228	2.00E-70	229	vanRF is a vanR variant found in the vanF gene cluster
UCC118_ORF_676	46	418	2.00E-121	441	Mycobacterium tuberculosis murA confers intrinsic resistance to fosfomycin
UCC118_ORF_717	69	396	0	395	Sequence variants of Streptomyces cinnamoneus elongation factor Tu that confer resistance to elfamycin antibiotics
UCC118_ORF_1015	40	583	1.00E-145	578	AdeC is the outer membrane factor of the AdeABC multidrug efflux complex
UCC118_ORF_1019	45	648	0	772	PBP1a is a penicillin-binding protein found in Streptococcus pneumoniae
UCC118_ORF_1271	41	233	4.00E-54	233	vanRE is a vanR variant found in the vanE gene cluster
UCC118_ORF_1489	40	222	7.00E-47	224	MacB is an ATP-binding cassette (ABC) transporter that exports macrolides with 14- or 15- membered lactones
UCC118_ORF_1501	45	305	1.00E-79	302	bcrA is an ABC transporter found in Bacillus licheniformis that confers bacitracin resistance
UCC118_ORF_1583	44	222	1.00E-61	227	MacB is an ATP-binding cassette (ABC) transporter that exports macrolides with 14- or 15- membered lactones
UCC118_ORF_1825	43	219	5.00E-53	233	MacB is an ATP-binding cassette (ABC) transporter that exports macrolides with 14- or 15- membered lactones
UCC119_ORF_52	45	225	1.00E-62	236	vanRM is a vanR variant found in the vanM gene cluster
UCC119_ORF_216	48	228	2.00E-70	229	vanRF is a vanR variant found in the vanF gene cluster
UCC119_ORF_408	50	498	3.00E-171	493	LsaA is an ABC efflux pump expressed in Enterococcus faecalis
UCC119_ORF_594	46	418	2.00E-121	441	Mycobacterium tuberculosis murA confers intrinsic resistance to fosfomycin
UCC119_ORF_634	69	396	0	395	Sequence variants of Streptomyces cinnamoneus elongation factor Tu that confer resistance to elfamycin antibiotics
UCC119_ORF_933	40	583	1.00E-145	578	AdeC is the outer membrane factor of the AdeABC multidrug efflux complex
UCC119_ORF_937	45	648	0	772	PBP1a is a penicillin-binding protein found in Streptococcus pneumoniae
UCC119_ORF_1189	41	233	4.00E-54	233	vanRE is a vanR variant found in the vanE gene cluster
UCC119_ORF_1436	40	222	7.00E-47	224	MacB is an ATP-binding cassette (ABC) transporter that exports macrolides with 14- or 15- membered lactones
UCC119_ORF_1447	45	305	1.00E-79	302	bcrA is an ABC transporter found in Bacillus licheniformis that confers bacitracin resistance
UCC119_ORF_1528	44	222	1.00E-61	227	MacB is an ATP-binding cassette (ABC) transporter that exports macrolides with 14- or 15- membered lactones
UCC119_ORF_1774	43	219	5.00E-53	233	MacB is an ATP-binding cassette (ABC) transporter that exports macrolides with 14- or 15- membered lactones

Table S9

gene	functional annotation	% identity	query coverage	e-value	query length
01M14315_ORF_1114	ATP-dependent_Clp_protease_proteolytic_subunit	72.59	197	1.00E-105	197
01M14315_ORF_1197	UDP-glucose_pyrophosphorylase	72.7	293	4.00E-151	290
778_ORF_1065	ATP-dependent_Clp_protease_proteolytic_subunit	72.59	197	1.00E-105	197
778_ORF_1147	UDP-glucose_pyrophosphorylase	72.7	293	2.00E-151	290
866_ORF_1162	ATP-dependent_Clp_protease_proteolytic_subunit	72.59	197	1.00E-105	197
866_ORF_1246	UDP-glucose_pyrophosphorylase	72.7	293	2.00E-151	290
ACS116_ORF_1278	ATP-dependent_Clp_protease_proteolytic_subunit	72.59	197	1.00E-105	197
ACS116_ORF_1363	UDP-glucose_pyrophosphorylase	72.7	293	2.00E-151	290
AH4231_ORF_1274	ATP-dependent_Clp_protease_proteolytic_subunit	72.59	197	1.00E-105	197
AH4231_ORF_1357	UDP-glucose_pyrophosphorylase	72.7	293	2.00E-151	290
AH4331_ORF_1249	ATP-dependent_Clp_protease_proteolytic_subunit	72.59	197	1.00E-105	197
AH4331_ORF_1331	UDP-glucose_pyrophosphorylase	72.7	293	2.00E-151	290
AH4331_ORF_2194	cell_wall_surface_anchor_family_protein,_Plasminogen-_and_Fibronectin-binding_protein_B	70.3	101	9.00E-43	105
AH43324_ORF_1245	ATP-dependent_Clp_protease_proteolytic_subunit	72.59	197	1.00E-105	197
AH43324_ORF_1326	UDP-glucose_pyrophosphorylase	72.7	293	2.00E-151	290
AH43348_ORF_1213	ATP-dependent_Clp_protease_proteolytic_subunit	72.59	197	1.00E-105	197
AH43348_ORF_1315	UDP-glucose_pyrophosphorylase	72.7	293	2.00E-151	290
ATCC11741_ORF_1065	ATP-dependent_Clp_protease_proteolytic_subunit	72.59	197	1.00E-105	197
ATCC11741_ORF_1147	UDP-glucose_pyrophosphorylase	72.7	293	2.00E-151	290
CCuG2753OB_ORF_1016	ATP-dependent_Clp_protease_proteolytic_subunit	72.59	197	1.00E-105	197
CCuG2753OB_ORF_1100	UDP-glucose_pyrophosphorylase	72.7	293	2.00E-151	290
CCuG38008_ORF_1126	ATP-dependent_Clp_protease_proteolytic_subunit	72.59	197	1.00E-105	197
CCuG38008_ORF_1209	UDP-glucose_pyrophosphorylase	72.7	293	2.00E-151	290
CCuG44481_ORF_1134	ATP-dependent_Clp_protease_proteolytic_subunit	72.59	197	1.00E-105	197
CCuG44481_ORF_1217	UDP-glucose_pyrophosphorylase	72.7	293	2.00E-151	290
CCuG45735_ORF_1169	ATP-dependent_Clp_protease_proteolytic_subunit	72.59	197	1.00E-105	197
CCuG45735_ORF_1251	UDP-glucose_pyrophosphorylase	72.7	293	2.00E-151	290
CCuG47171_ORF_1127	ATP-dependent_Clp_protease_proteolytic_subunit	72.59	197	1.00E-105	197
CCuG47171_ORF_1233	UDP-glucose_pyrophosphorylase	72.7	293	2.00E-151	290
CCuG47825_ORF_396	polysaccharide_biosynthesis_protein_CpsF	73.63	91	5.00E-46	93
CCuG47825_ORF_1465	ATP-dependent_Clp_protease_proteolytic_subunit	72.59	197	1.00E-105	197
CCuG47825_ORF_2004	UDP-glucose_pyrophosphorylase	72.35	293	7.00E-151	290
CCuG47826_ORF_1150	ATP-dependent_Clp_protease_proteolytic_subunit	72.59	197	1.00E-105	197
CCuG47826_ORF_1339	UDP-glucose_pyrophosphorylase	72.35	293	7.00E-151	290
CECT5713_ORF_1313	ATP-dependent_Clp_protease_proteolytic_subunit	72.59	197	1.00E-105	197
CECT5713_ORF_1425	UDP-glucose_pyrophosphorylase	72.7	293	2.00E-151	290
cp400_ORF_1280	ATP-dependent_Clp_protease_proteolytic_subunit	72.59	197	1.00E-105	197
cp400_ORF_1413	UDP-glucose_pyrophosphorylase	73.04	293	4.00E-152	290
DSM18933_ORF_62	UDP-glucose_pyrophosphorylase	73.04	293	7.00E-153	300
DSM18933_ORF_219	polysaccharide_biosynthesis_protein_CpsF	74.5	149	7.00E-86	149
DSM18933_ORF_1644	ATP-dependent_Clp_protease_proteolytic_subunit	73.58	193	1.00E-104	196
DSM20492_ORF_1084	ATP-dependent_Clp_protease_proteolytic_subunit	72.59	197	1.00E-105	197
DSM20492_ORF_1168	UDP-glucose_pyrophosphorylase	72.7	293	2.00E-151	290
DSM20492_ORF_1456	polysaccharide_biosynthesis_protein_CpsF	75	132	5.00E-73	132
DSM20554_ORF_1092	ATP-dependent_Clp_protease_proteolytic_subunit	72.59	197	1.00E-105	197
DSM20554_ORF_1174	UDP-glucose_pyrophosphorylase	72.7	293	2.00E-151	290
DSM20554_ORF_1467	polysaccharide_biosynthesis_protein_CpsF	75	132	5.00E-73	132
DSM20555_ORF_1055	ATP-dependent_Clp_protease_proteolytic_subunit	72.59	197	1.00E-105	197
DSM20555_ORF_1138	UDP-glucose_pyrophosphorylase	72.7	293	2.00E-151	290
GJ24_ORF_1096	ATP-dependent_Clp_protease_proteolytic_subunit	72.59	197	1.00E-105	197
GJ24_ORF_1189	UDP-glucose_pyrophosphorylase	72.7	293	2.00E-151	290
gul1_ORF_1025	ATP-dependent_Clp_protease_proteolytic_subunit	72.59	197	1.00E-105	197
gul1_ORF_1107	UDP-glucose_pyrophosphorylase	72.7	293	2.00E-151	290
gul2_ORF_1067	ATP-dependent_Clp_protease_proteolytic_subunit	72.59	197	1.00E-105	197
gul2_ORF_1150	UDP-glucose_pyrophosphorylase	72.7	293	2.00E-151	290
JCM1040_ORF_1109	ATP-dependent_Clp_protease_proteolytic_subunit	72.59	197	1.00E-105	197
JCM1040_ORF_1192	UDP-glucose_pyrophosphorylase	72.7	293	2.00E-151	290
JCM1042_ORF_1056	ATP-dependent_Clp_protease_proteolytic_subunit	72.59	197	1.00E-105	197
JCM1042_ORF_1141	UDP-glucose_pyrophosphorylase	72.7	293	2.00E-151	290
JCM1044_ORF_1016	ATP-dependent_Clp_protease_proteolytic_subunit	72.59	197	1.00E-105	197
JCM1044_ORF_1100	UDP-glucose_pyrophosphorylase	72.7	293	2.00E-151	290
JCM1045_ORF_1131	ATP-dependent_Clp_protease_proteolytic_subunit	72.59	197	1.00E-105	197
JCM1045_ORF_1214	UDP-glucose_pyrophosphorylase	72.7	293	2.00E-151	290
JCM1046_ORF_1085	ATP-dependent_Clp_protease_proteolytic_subunit	72.59	197	1.00E-105	197
JCM1046_ORF_1167	UDP-glucose_pyrophosphorylase	73.04	293	4.00E-152	290
JCM1046_ORF_1500	polysaccharide_biosynthesis_protein_CpsF	75.17	149	9.00E-84	152
JCM1047_ORF_1287	ATP-dependent_Clp_protease_proteolytic_subunit	72.59	197	1.00E-105	197
JCM1047_ORF_1370	UDP-glucose_pyrophosphorylase	72.7	293	2.00E-151	290
JCM1230_ORF_1032	ATP-dependent_Clp_protease_proteolytic_subunit	72.59	197	1.00E-105	197
JCM1230_ORF_1160	UDP-glucose_pyrophosphorylase	72.7	293	2.00E-151	290
L21_ORF_1211	ATP-dependent_Clp_protease_proteolytic_subunit	72.59	197	1.00E-105	197
L21_ORF_1294	UDP-glucose_pyrophosphorylase	72.7	293	2.00E-151	290
LMG14476_ORF_1187	ATP-dependent_Clp_protease_proteolytic_subunit	72.59	197	1.00E-105	197
LMG14476_ORF_1272	UDP-glucose_pyrophosphorylase	72.7	293	2.00E-151	290
LMG14477_ORF_1171	ATP-dependent_Clp_protease_proteolytic_subunit	72.59	197	1.00E-105	197
LMG14477_ORF_1256	UDP-glucose_pyrophosphorylase	72.7	293	2.00E-151	290
NCIMB702343_ORF_1073	ATP-dependent_Clp_protease_proteolytic_subunit	72.59	197	1.00E-105	197
NCIMB702343_ORF_1158	UDP-glucose_pyrophosphorylase	72.7	293	2.00E-151	290
NCIMB702343_ORF_1488	polysaccharide_biosynthesis_protein_CpsF	77.14	140	7.00E-79	141
NCIMB8816_ORF_1018	ATP-dependent_Clp_protease_proteolytic_subunit	72.59	197	1.00E-105	197
NCIMB8816_ORF_1141	UDP-glucose_pyrophosphorylase	72.7	293	2.00E-151	290
NCIMB8816_ORF_1869	cell_wall_surface_anchor_family_protein,_Plasminogen-_and_Fibronectin-binding_protein_B	70	110	4.00E-47	110
NCIMB8817_ORF_1119	ATP-dependent_Clp_protease_proteolytic_subunit	72.59	197	1.00E-105	197
NCIMB8817_ORF_1201	UDP-glucose_pyrophosphorylase	72.7	293	2.00E-151	290
NCIMB8818_ORF_1316	ATP-dependent_Clp_protease_proteolytic_subunit	72.59	197	1.00E-105	197
NCIMB8818_ORF_1399	UDP-glucose_pyrophosphorylase	72.7	293	2.00E-151	290
NIAS840_ORF_276	UDP-glucose_pyrophosphorylase	72.7	293	2.00E-151	290
NIAS840_ORF_577	polysaccharide_biosynthesis_protein_CpsF	75	132	5.00E-73	132

NIAS840_ORF_1603	ATP-dependent_Clp_protease_proteolytic_subunit	72.59	197	1.00E-105	197
Ren_ORF_1171	ATP-dependent_Clp_protease_proteolytic_subunit	72.59	197	1.00E-105	197
Ren_ORF_1266	UDP-glucose_pyrophosphorylase	72.35	293	4.00E-150	290
SMXD51_ORF_1498	ATP-dependent_Clp_protease_proteolytic_subunit	72.59	197	1.00E-105	197
SMXD51_ORF_1595	UDP-glucose_pyrophosphorylase	72.7	293	2.00E-151	290
UCC118_ORF_1257	ATP-dependent_Clp_protease_proteolytic_subunit	72.59	197	1.00E-105	197
UCC118_ORF_1367	UDP-glucose_pyrophosphorylase	72.7	293	2.00E-151	290
